# Glutathione *S*-transferase activity facilitates rice tolerance to the barnyard grass root exudate DIMBOA

**DOI:** 10.1186/s12870-024-04802-5

**Published:** 2024-02-17

**Authors:** Huabin Zhang, Dan Mu, Yushan Li, Xilin Li, Xue Yan, Ke Li, Yanyang Jiao, Jiayu Li, Hongmei Lin, Wenxiong Lin, Changxun Fang

**Affiliations:** 1 Fujian Provincial Key Laboratory of Agroecological Processing and Safety Monitoring, College of JunCao Science and Ecology, Fujian Agriculture and Forestry University, Fuzhou, 350002 China; 2 Key Laboratory of Ministry of Education for Genetics, Breeding and Multiple Utilization of Crops, Fujian Agriculture and Forestry University, Fuzhou, 350002 China; 3 Key Laboratory of Crop Ecology and Molecular Physiology, Fujian Agriculture and Forestry University, Fujian Province University, Fuzhou, 350002 China; 4 Institute of Oceanography, College of Geography and Oceanography, Minjiang University, Fuzhou, 350108 China

**Keywords:** Barnyard grass, DIMBOA, Glutathione metabolism, GST, Proteomics, Tolerance

## Abstract

**Background:**

In paddy fields, the noxious weed barnyard grass secretes 2,4-dihydroxy-7-methoxy-2*H*-1,4-benzoxazin-3(4*H*)-one (DIMBOA) to interfere with rice growth. Rice is unable to synthesize DIMBOA. Rice cultivars with high or low levels of allelopathy may respond differently to DIMBOA.

**Results:**

In this study, we found that low concentrations of DIMBOA (≤ 0.06 mM) promoted seedling growth in allelopathic rice PI312777, while DIMBOA (≤ 0.08 mM) had no significant influence on the nonallelopathic rice Lemont. DIMBOA treatment caused changes in the expression of a large number of glutathione *S-*transferase (GST) proteins, which resulting in enrichment of the glutathione metabolic pathway. This pathway facilitates plant detoxification of heterologous substances. The basal levels of GST activity in Lemont were significantly higher than those in PI312777, while GST activity in PI312777 was slightly induced by increasing DIMBOA concentrations. Overexpression of *GST* genes (*Os09g0367700* and *Os01g0949800*) in these two cultivars enhanced rice resistance to DIMBOA.

**Conclusions:**

Taken together, our results indicated that different rice accessions with different levels of allelopathy have variable tolerance to DIMBOA. Lemont had higher GST activity, which helped it tolerate DIMBOA, while PI312777 had lower GST activity that was more inducible. The enhancement of *GST* expression facilitates rice tolerance to DIMBOA toxins from barnyard grass root exudates.

**Supplementary Information:**

The online version contains supplementary material available at 10.1186/s12870-024-04802-5.

## Background

Weeds are a serious biotic stressor that reduces rice yields, and barnyard grass is a noxious weed in rice fields that causes a significant loss of yield. Studies have documented that barnyard grass invasion leads to losses ranging from 1.5 to 55.2% of rice yield annually [1], which has led to an increase in herbicide application, resulting in enhanced herbicide resistance in weeds and potential herbicide residues remaining in the environment [2].

In the natural system of rice and weed, there exist rice germplasm materials that can suppress weed growth through allelopathy, which arises from their high capacity for secondary metabolite synthesis and secretion. These compounds include phenolic acids, diterpenoids, and flavonoids, which are recognized as allelochemicals, and these rice accessions are referred to as allelopathic rice [3]. The presence of weeds activates higher transcriptional levels of *phenylalanine ammonia-lyase* (*OsPAL*), *cinnamate-4-hydroxylase* (*OsC4H*), *cinnamyl*-*alcohol dehydrogenase *(*OsCAD*), *momilactone A synthase* (*OsMAS*), and *ent-kaurene synthase-like 4* (*OsKSL4*), which enhances the biosynthesis of allelochemicals. Sufficient concentrations of these compounds from root exudates are then released into the rhizosphere, resulting in allelopathic inhibition of the surrounding weeds [4–8].

Fang et al. (2015) indicated that phenolic acid allelochemicals promoted the proliferation of *Myxococcus sp.* in the rhizosphere, and the combination of ferulic acid and *Myxococcus xanthus* led to strong growth inhibition of barnyard grass, which was due to the increase in apurinic/apyrimidinic (AP) sites and the decrease in IAA contents in barnyard grass roots. The increase in DNA damage and reduction in hormone contents suppressed the growth of barnyard grass [9]. Studies have also focused on the allelopathic potential of diterpenoids (momilactone A and momilactone B) and flavonoids against this weed and their impact on soil microorganisms [5, 7]. These characteristics of allelopathic rice cultivars enable their biointerference with weed growth and are considered to be natural survival strategies of rice. In contrast, the weed (barnyard grass) has also evolved unique characteristics to restrict rice growth. Genome sequencing and assembly along with gene annotation of barnyard grass (*Echinochloa crus-galli* L.) showed that a gene cluster for DIMBOA synthesis was found in the genome of *E. crus-galli*, but the gene cluster was absent from the rice genome. The presence of the DIMBOA gene cluster in *E. crus-galli* enables the biosynthesis of these compounds in barnyard grass, and a bioassay of the effects of DIMBOA on rice showed that 0.08 mM DIMBOA significantly suppresses rice growth [10].

DIMBOA, 2,4-dihydroxy-7-methoxy-2*H*-1,4-benzoxazin-3(4*H*)-one, is a compound that widely exists in gramineous crops and is a secondary metabolite with antibacterial activity and pest resistance [11]. In wheat and maize, DIMBOA is a vital allelochemical [12], and the concentrations of DIMBOA in wheat can be induced by co-cultured weeds, i.e., *Abutilon theophrasti* Medicus, *Alopecurus Xaponicas* Steud, and *Avena fatua* L.; thus, DIMBOA is regarded as an important and dominant allelochemical against weeds of wheat [13]. The absence of the DIMBOA synthesis gene cluster in rice has resulted in the loss of DIMBOA synthesis; in contrast, the DIMBOA from coexisting barnyard grass is a particularly toxic compound that suppresses rice growth and acts as a biointerference molecule.

Pathways involved in the detoxification of xenobiotics in plants can be classified into three phases. These phases require the involvement of Cytochrome P450s (CYP450s), Glutathione S-transferases (GSTs), and ATP-binding cassette (ABC) transporters, also known as the multixenobiotic resistance (MXR) system, in phases I, II, and III respectively [14]. CYP450s catalyze a range of oxidation and reduction reactions to degrade toxic xenobiotics, transforming them into more polar and water-soluble compounds [15, 16]. Meanwhile, GST enzymes enhance the conjugation of xenobiotics with glutathione (GSH), facilitating their further detoxification within plant cells. Through this conjugation process, polar groups are added to the xenobiotics, aiding in their detoxification [15]. In addition to these steps, an essential aspect of detoxification is the excretion of xenobiotics and/or their metabolites by the MXR system. This system helps transport these compounds out of the plant, further contributing to the detoxification process [14].

Since different allelopathic rice accessions have different levels of allelopathy against the weed, whether there are different levels of tolerance or resistance to DIMBOA toxicity remains unknown. This study compared the different levels of DIMBOA tolerance between the allelopathic rice accession PI312777 and the nonallelopathic rice accession Lemont. PI312777 was developed in the Philippines from indica parents (T65*2/TN 1), both of which originated from Taiwan, China [17, 18]. This particular variety has shown strong suppression against barnyard grass; Lemont, on the other hand, was developed through a cross made in 1974 between ‘Lebonnet’ and the F1 resulting from crossing CI 9881 with PI 331,581 [19]. The proteomic expression profile of these two rice accessions was elucidated, and the function of key proteins and their main pathway was illuminated.

## Results

### Effects of DIMBOA on the growth of PI312777 and Lemont

**Fig. 1:**
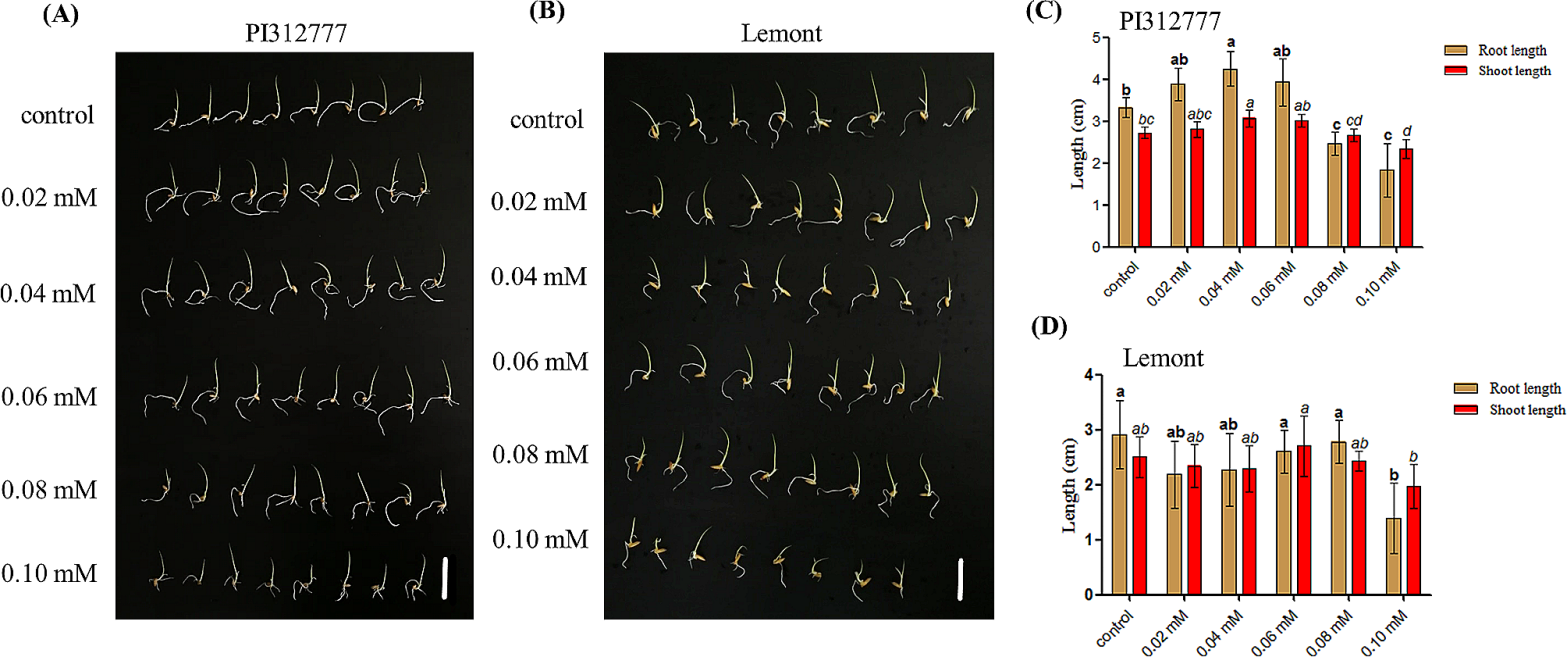
Growth effect of different DIMBOA concentrations on the allelopathic rice accession PI312777 and nonallelopathic rice Lemont. Germinated seeds of PI312777 (**A, C**) and Lemont (**B, D**) were soaked in 0.02 mM, 0.04 mM, 0.06 mM, 0.08 mM, and 0.10 mM DIMBOA, while germinated seeds cultured in sterilized ddH_2_O were used as control groups. After 4 days, rice root and shoot lengths from the control and treatment groups were measured. Error bars indicate standard deviation. Significant differences (*p* < 0.05) in rice roots and shoots between different treatments are indicated by different lowercase letters according to analysis of variance (ANOVA). Lowercase letters with bold correspond to the root length comparison, and those letters with italics correspond to the shoot length comparison. Bar, 3 cm

The root and stem lengths of PI312777 and Lemont treated with 0.02 mM, 0.04 mM, 0.06 mM, 0.08 mM and 0.10 mM DIMBOA and those of their control groups without DIMBOA were determined. The results showed that PI312777 and Lemont have different levels of tolerance to DIMBOA toxicity. The growth of roots and stems was promoted in PI312777 under 0.02 mM, 0.04 mM and 0.06 mM DIMBOA treatment and peaked at 0.04 mM DIMBOA treatment with a significant promoting effect, whereas 0.08 mM and 0.10 mM DIMBOA significantly inhibited the root and stem length of PI312777. In contrast, DIMBOA at concentrations up to 0.08 mM did not significantly affect the growth of roots and stems of Lemont, while 0.10 mM DIMBOA significantly inhibited root growth but did not significantly affect stem growth in Lemont (Fig. 1). The different levels of tolerance of the allelopathic rice PI312777 and nonallelopathic rice Lemont to DIMBOA toxicity suggest different strategies of high and low allelopathic potential in response to the biointerference of barnyard grass.

### Differentially expressed proteins (DEPs) in PI312777 and Lemont

**Fig. 2:**
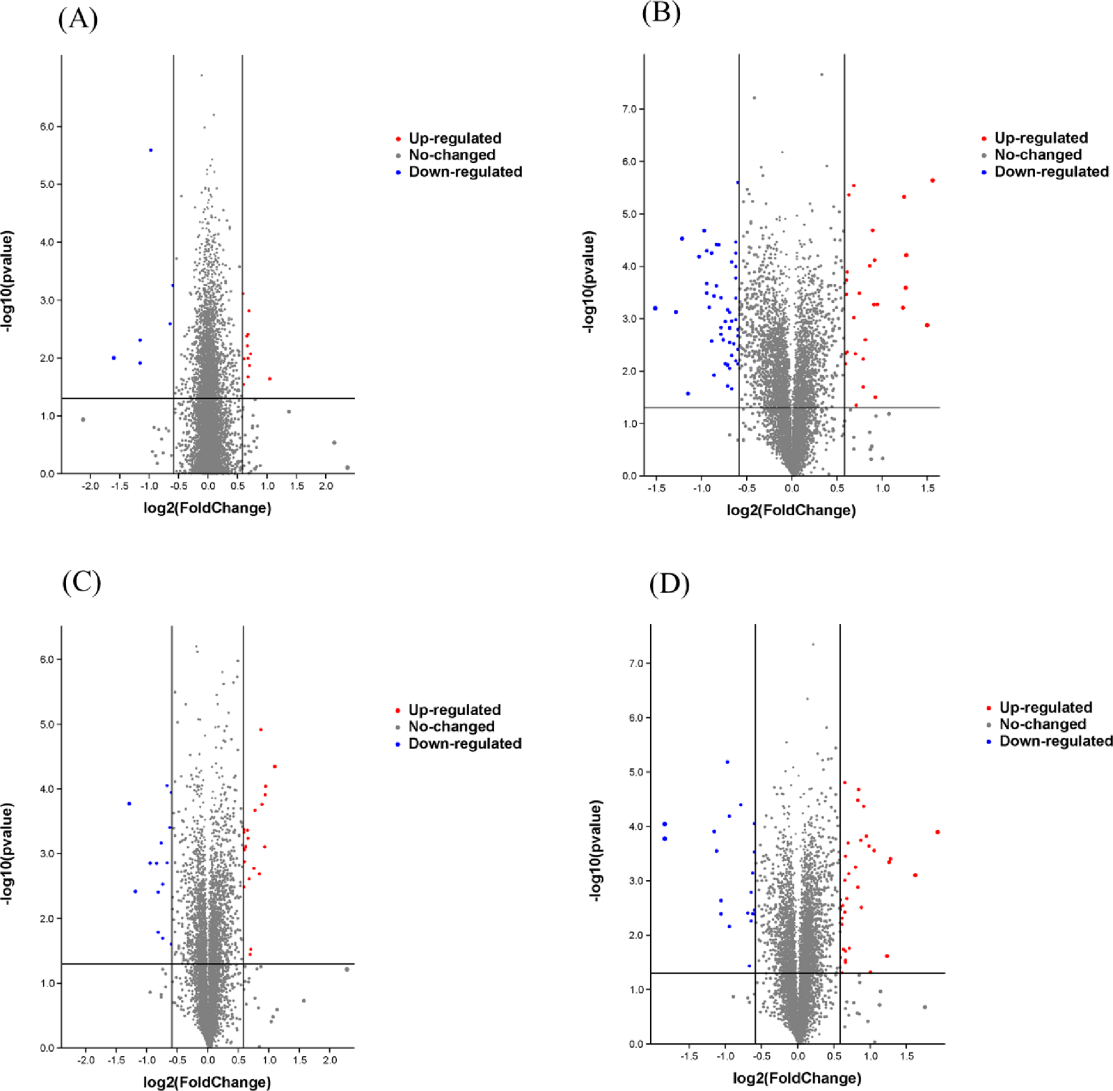
Volcano plot demonstrating the differentially expressed proteins (DEPs) from PI312777 and Lemont under DIMBOA treatment. The differentially expressed proteins between the treatment and control groups from PI312777 and Lemont were selected to draw the volcano map by using fold change and *p* values. The pairwise comparisons were as follows: 0.04 mM DIMBOA-treated PI312777 compared to its control group without DIMBOA addition (**A**), 0.06 mM DIMBOA-treated PI312777 compared to its control group (**B**), 0.04 mM DIMBOA-treated Lemont compared to its control group (**C**), and 0.06 mM DIMBOA-treated Lemont compared to its control group (**D**). The red dot represents significantly upregulated proteins, the blue dot represents significantly downregulated proteins (fold change ≥ 1.5), and the grey dot represents the proteins without a significant fold change

Seedlings of PI312777 and Lemont were treated with 0.04 mM and 0.06 mM DIMBOA, respectively (Fig. S1), and iTRAQ quantitative proteomics was conducted to explore differentially expressed proteins (DEPs) between the DIMBOA-treated rice and the control group without DIMBOA treatment. The results showed that 231 proteins were upregulated and 135 proteins were downregulated in the roots of PI312777 treated with 0.04 mM DIMBOA, while 211 proteins were upregulated and 450 proteins were downregulated in the roots of PI312777 treated with 0.06 mM DIMBOA compared to the control group for PI312777. In Lemont, 0.04 mM DIMBOA treatment resulted in 236 proteins being upregulated and 144 proteins being downregulated in the root, and 0.06 mM DIMBOA treatment resulted in 268 proteins being upregulated and 175 proteins being downregulated in the root when compared with the control group for Lemont (Fig. S2). Prediction of the protein‒protein interactions of the DEPs showed that most of the DEPs from the treatment group and control group closely interacted, especially the DEPs from PI312777 treated with 0.06 mM DIMBOA (Fig. S3). Among these DEPs, the expression of 41 and 40 proteins was significantly upregulated (fold change ≥ 1.5, the same below) in PI312777 under 0.04 mM and 0.06 mM DIMBOA treatment, in comparison with control group respectively, while the expression of 17 and 52 proteins was significantly downregulated. In Lemont, the expression of 38 and 53 proteins was significantly upregulated in the 0.04 mM and 0.06 mM DIMBOA-treated groups, respectively (Fig. 2).

### Pathway enrichment of DEPs

**Fig. 3:**
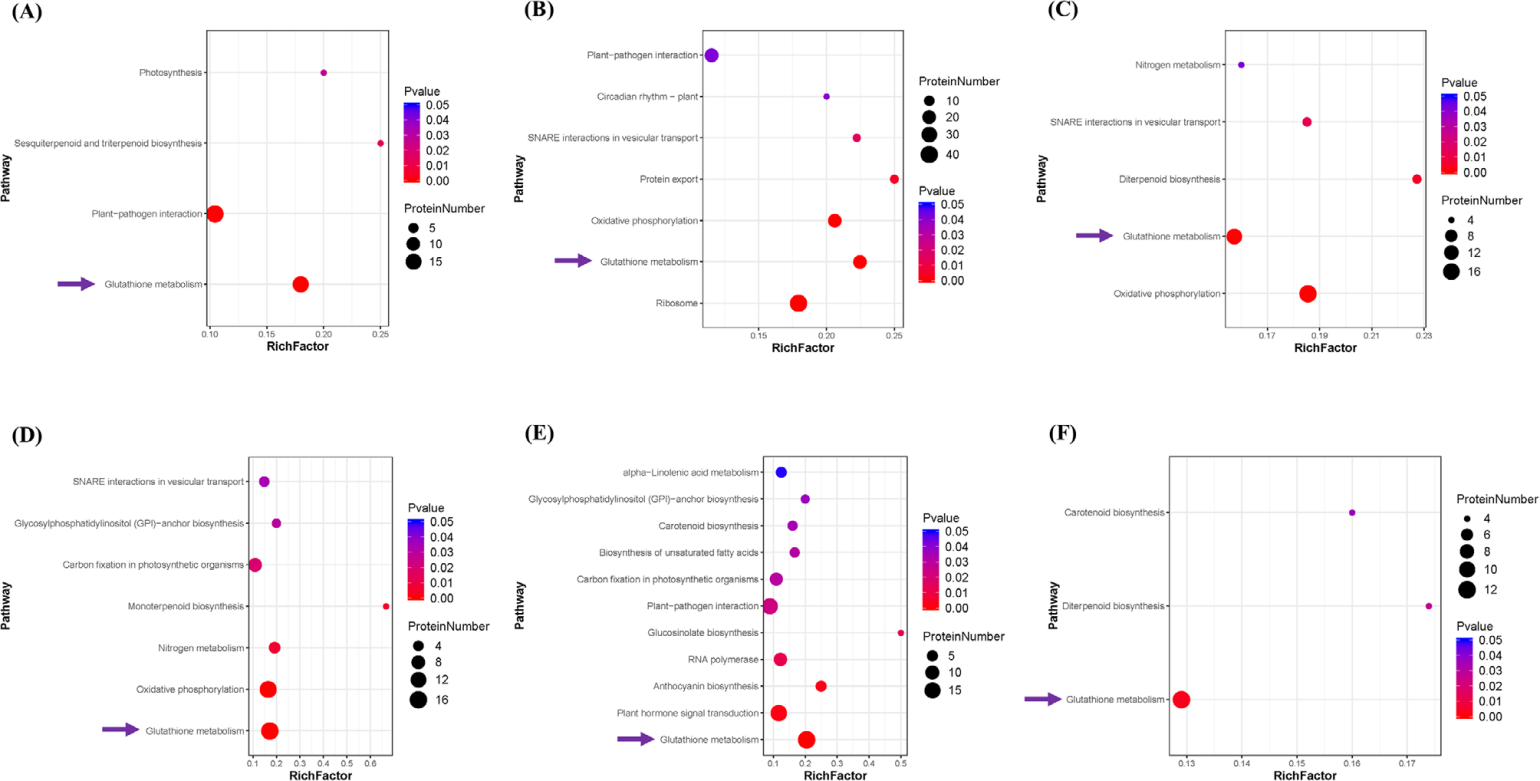
KEGG enrichment of DEPs in PI312777 and Lemont under DIMBOA treatment. The DEPs between the treatment and control groups for PI312777 and Lemont were annotated with KEGG pathways to determine pathway enrichment. The pairwise comparisons were as follows: 0.04 mM DIMBOA-treated PI312777 compared to its control group without DIMBOA addition (**A**), 0.06 mM DIMBOA-treated PI312777 compared to its control group (**B**), 0.06 mM DIMBOA-treated PI312777 compared to 0.04 mM DIMBOA-treated PI312777 (**C**), 0.04 mM DIMBOA-treated Lemont compared to its control group (**D–E**), 0.06 mM DIMBOA-treated Lemont compared to its control group, and 0.06 mM DIMBOA-treated Lemont compared to 0.04 mM DIMBOA-treated Lemont (**F**). The arrows point to the uniformly enriched pathway (glutathione metabolism) among all pairwise comparisons

DEPs from each individual treatment were annotated with relevant pathways to explore KEGG pathway enrichment. The results showed that the DEPs from 0.04 mM DIMBOA-treated PI312777 (PI-TR1) were mainly enriched in glutathione metabolism, plant‒pathogen interaction, sesquiterpenoid and triterpenoid biosynthesis, and photosynthesis (Fig. 3A), while the DEPs from 0.06 mM DIMBOA-treated PI312777 (PI-TR2) were mainly enriched in ribosomes, glutathione metabolism, oxidative phosphorylation, plant‒pathogen interaction, protein export, biological clock, SNARE interactions in vesicular transport and plant circadian rhythm (Fig. 3B). When comparing PI-TR2 and PI-TR1, most DEPs were enriched in glutathione metabolism, oxidative phosphorylation, diterpenoid biosynthesis, SNARE interactions in vesicular transport and nitrogen metabolism. Taken together, glutathione metabolism and the relevant DEPs participating in this pathway were involved in the regulation of PI312777 tolerance to DIMBOA (Fig. 3C).

The DEPs in the Lemont treated with 0.04 mM DIMBOA (LE-TR1) were mainly enriched in glutathione metabolism, oxidative phosphorylation, nitrogen metabolism, monoterpenoid synthesis, carbon fixation in photosynthetic organisms, GPI-anchor biosynthesis, and SNARE interactions in vesicular transport (Fig. 3D). Those in Lemont treated with 0.06 mM DIMBOA (LE-TR2) were significantly enriched in glutathione metabolism, plant hormone signal transduction, anthocyanin synthesis, RNA polymerase, glucosinolate biosynthesis, plant‒pathogen interaction, and carbon fixation in photosynthetic organisms. (Fig. 3E). The DEPs between LE-TR2 and LE-TR1 were also mainly enriched in glutathione metabolism (Fig. 3F). The results of iTRAQ quantitative proteomics for PI312777 and Lemont indicated that glutathione metabolism is indispensable in rice tolerance to DIMBOA.

### Changes in glutathione metabolic pathway-related protein expression levels

**Fig. 4:**
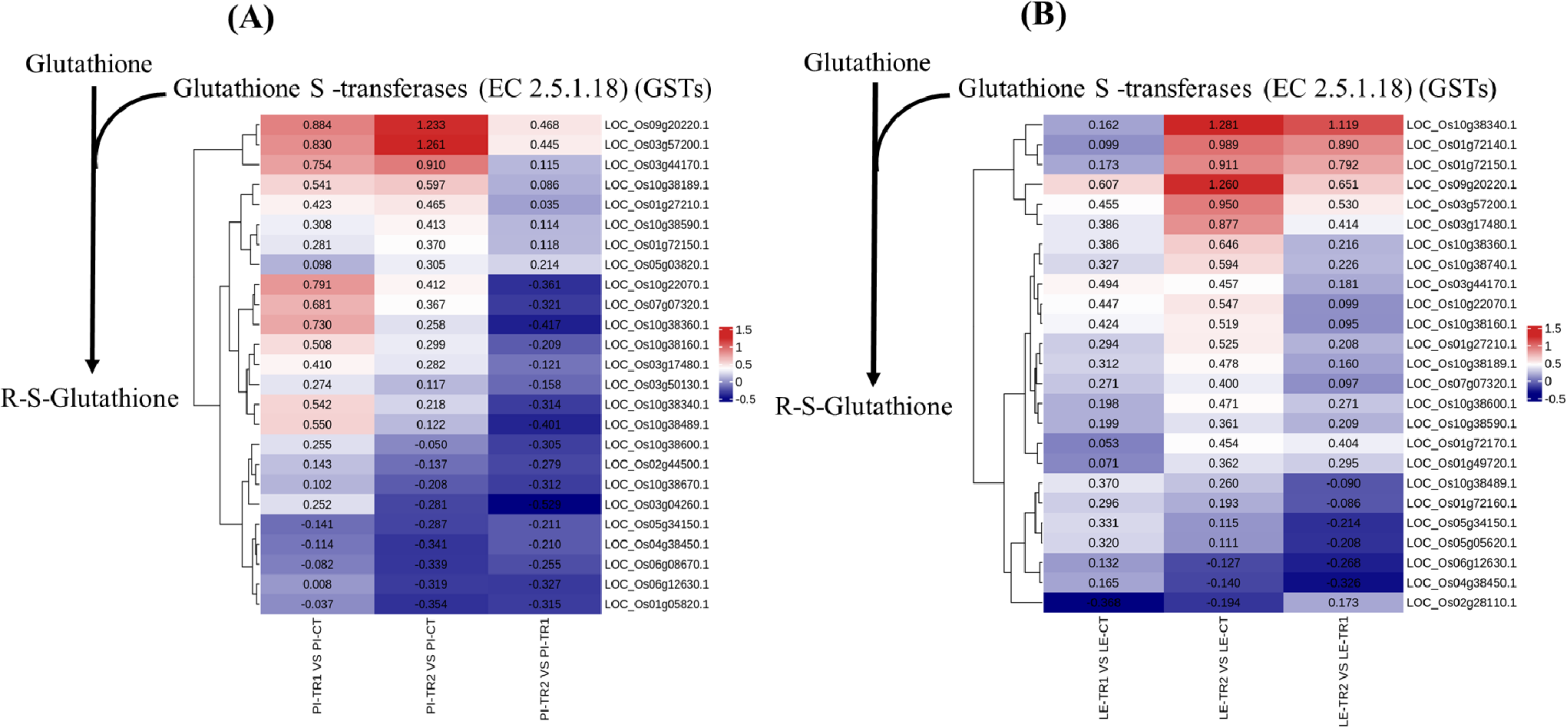
Expression changes in GSTs participating in glutathione metabolism in the roots of PI312777 (**A**) and Lemont (**B**) under DIMBOA treatment. The differentially expressed GSTs in PI312777 and Lemont that participate in glutathione metabolism were selected for a comparison of their fold changes in expression and heatmap analysis. The data in each column represent the fold change in independent proteins based on pairwise comparisons of PI312777 (**A**) and Lemont (**B**), including 0.04 mM DIMBOA-treated PI312777 compared to its control group without DIMBOA addition (PI-TR1 vs. PI-CT), 0.06 mM DIMBOA-treated PI312777 compared to its control group (PI-TR2 vs. PI-CT), 0.06 mM DIMBOA-treated PI312777 compared to 0.04 mM DIMBOA-treated PI312777 (PI-TR2 vs. PI-TR1), 0.04 mM DIMBOA-treated Lemont compared to its control group (LE-TR1 vs. LE-CT), 0.06 mM DIMBOA-treated Lemont compared to its control group (LE-TR2 vs. LE-CT), and 0.06 mM DIMBOA-treated Lemont compared to 0.04 mM DIMBOA-treated Lemont (LE-TR2 vs. LE-TR1)

The expression levels of DEPs involved in glutathione metabolism were analysed. The results showed that the expression levels of several glutathione *S*-transferases (GSTs) involved in this pathway were changed in PI312777 and Lemont treated with DIMBOA. The expression patterns of PI312777 and Lemont were also different. In PI312777, treatment with 0.04 mM and 0.06 mM DIMBOA resulted in the upregulation of 8 proteins, LOC_Os03g44170.1 (Os03g0643700), LOC_Os03g57200.1 (Os03g0785900), LOC_Os09g20220.1 (Os09g0367700), LOC_Os10g38189.1 (Os10g0525800), LOC_Os01g27210.1 (Os01g0369700), LOC_Os05g03820.1 (Os05g0129000), LOC_Os10g38590.1 (Os05g0129000), and LOC_Os01g72150.1 (Os01g0949900). The relative protein expression increased with increasing DIMBOA concentration (0.04 mM and 0.06 mM) (Fig. 4A). In Lemont, seventeen proteins, LOC_Os10g38340.1 (Os10g0527400), LOC_Os01g72150.1 (Os01g0949900), LOC_Os01g72140.1 (Os01g0949800), LOC_Os09g20220.1 (Os09g0367700), LOC_Os03g57200.1 (Os03g0785900), LOC_Os03g17480.1 (Os03g0283200), LOC_Os01g72170.1 (Os01g0950300), LOC_Os01g49720.1 (Os01g0692100), LOC_Os10g38600.1 (Os10g0529500), LOC_Os10g38590.1 (Os10g0529400), LOC_Os07g07320.1 (Os07g0168300), LOC_Os10g38189.1 (Os10g0525800), LOC_Os01g27210.1 (Os01g0369700), LOC_Os10g38740.1 (Os10g0530900), LOC_Os10g38360.1 (Os10g0527800), LOC_Os10g38160.1, and LOC_Os10g22070.1 (Os10g0365200), were all upregulated in the 0.04 mM DIMBOA and 0.06 mM DIMBOA treatment groups, and the relative protein expression increased with increasing concentration of exogenous DIMBOA (Fig. 4B). These results suggested that glutathione metabolism plays a dominant role in the regulation of rice tolerance.

### Changes in GST gene expression in PI312777 and lemont roots under DIMBOA treatment

**Fig. 5:**
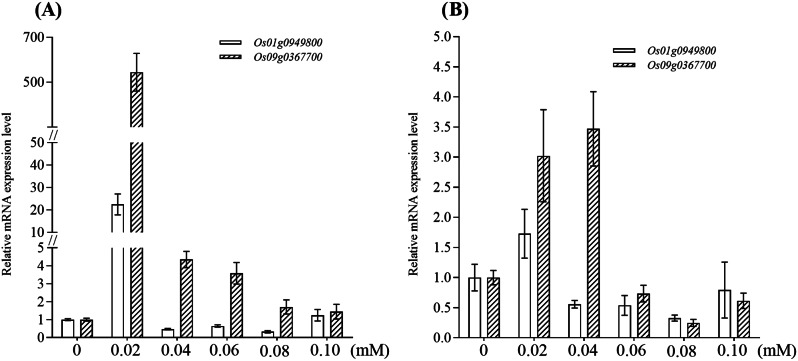
Expression changes for GST in the roots of PI312777 (**A**) and Lemont (**B**) under DIMBOA treatment. The transcript levels of *Os01g0949800* and *Os09g0367700* in PI312777 (**A**) and Lemont (**B**) treated with 0.02 mM-0.10 mM were determined and compared to the control groups

The transcriptional levels of *Os01g0949800* and *Os09g0367700*, two genes encoding glutathione *S*-transferase (GST), in PI312777 and Lemont roots treated with DIMBOA at different concentrations were detected. The results showed that compared with the control group, the *Os01g0949800* gene in PI312777 roots was upregulated 22-fold under 0.02 mM DIMBOA treatment. When the exogenous concentration of DIMBOA was increased to 0.04 mM, 0.06 mM and 0.08 mM, the *Os01g0949800* gene was downregulated in the treated group compared with the control group. The expression of the *Os09g0367700* gene was upregulated in PI312777 treated with 0.02–0.10 mM DIMBOA, and compared with the control group, this gene was upregulated 544-fold in the 0.02 mM DIMBOA-treated group (Fig. 5A).

In Lemont, the expression of *Os01g0949800* was upregulated 1.7-fold under 0.02 mM DIMBOA treatment, whereas it was downregulated when the DIMBOA concentration was increased to 0.04 mM or higher. *Os09g0367700* gene expression was upregulated 3.02- and 3.47-fold under 0.02 mM and 0.04 mM DIMBOA treatment, respectively. Gene expression was downregulated in Lemont when the DIMBOA concentration was increased to 0.06 mM or higher (Fig. 5B).

When comparing the expression changes in *Os01g0949800* and *Os09g0367700* in PI312777 and Lemont under DIMBOA treatment, it was found that *GST* gene expression in PI312777 was more strongly induced by low concentrations of DIMBOA.

### GST activity in the roots of PI312777 and lemont

**Fig. 6:**
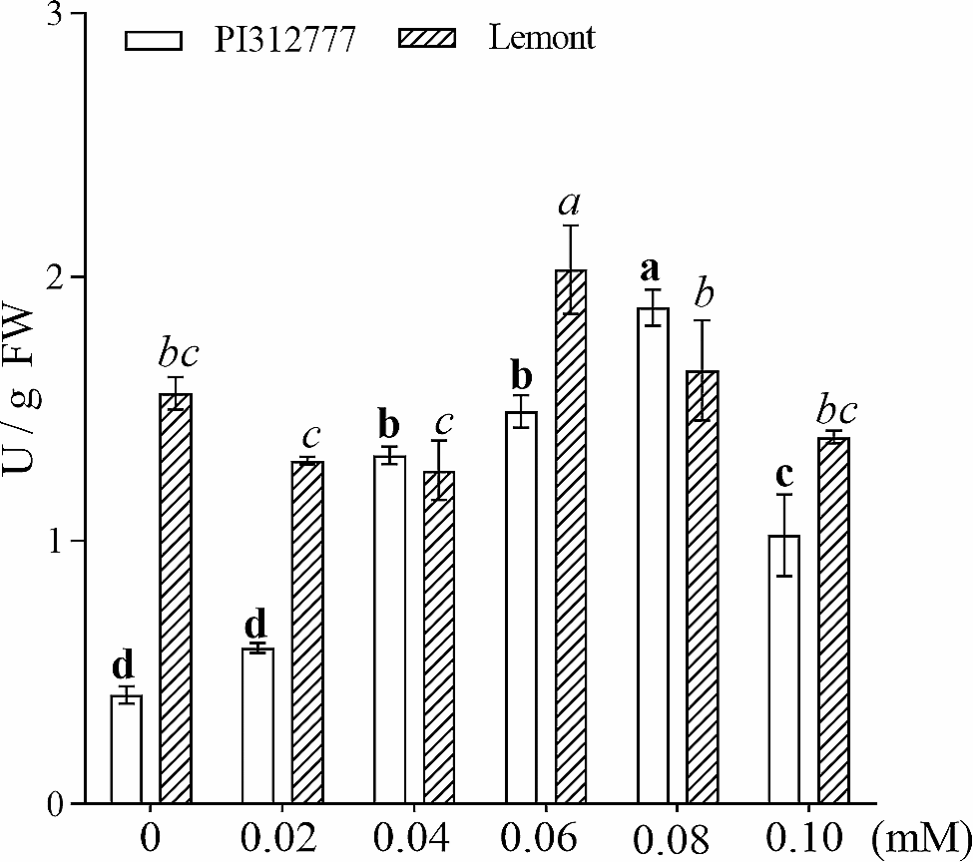
GST activity in the roots of PI312777 and Lemont under DIMBOA treatment. GST activities in the roots of PI312777 and Lemont treated with 0.02 mM-0.10 mM were determined and compared to the control groups. Error bars indicate standard deviation. Significant differences (*p* < 0.05) among different treatments are indicated by different lowercase letters according to analysis of variance (ANOVA). Lowercase letters with bold correspond to the PI312777 treatment comparison, and those letters with italics correspond to the Lemont treatment comparison

The GST activity in the roots of PI312777 was enhanced with increasing DIMBOA concentrations. The GST activity of PI312777 peaked at 1.88 U/g fresh weight under the 0.08 mM DIMBOA treatment. When the concentration of DIMBOA reached 0.10 mM, the GST activity was 1.02 U/g fresh weight, which was still significantly higher than that of the control group. In comparison with that of PI312777, the GST activity of Lemont rice roots under normal conditions (1.55 U/g fresh weight) was significantly higher than that of PI312777 roots under the same conditions (0.41 U/g fresh weight). Under 0.06 mM DIMBOA treatment, the GST activity of Lemont roots was significantly increased to 2.03 U/g fresh weight, while that under the other treatments showed no significant change compared with the control group (Fig. 6). A comparison of the GST activities in PI312777 and Lemont rice roots showed that the nonallelopathic rice Lemont had higher GST activity, which helped relieve the physiological toxicity of DIMBOA. The GST activity of the allelopathic rice PI312777 was lower than that of Lemont, but the GST activity of PI312777 was induced by DIMBOA treatment.

### GST overexpression in transgenic rice affects tolerance to DIMBOA

**Fig. 7:**
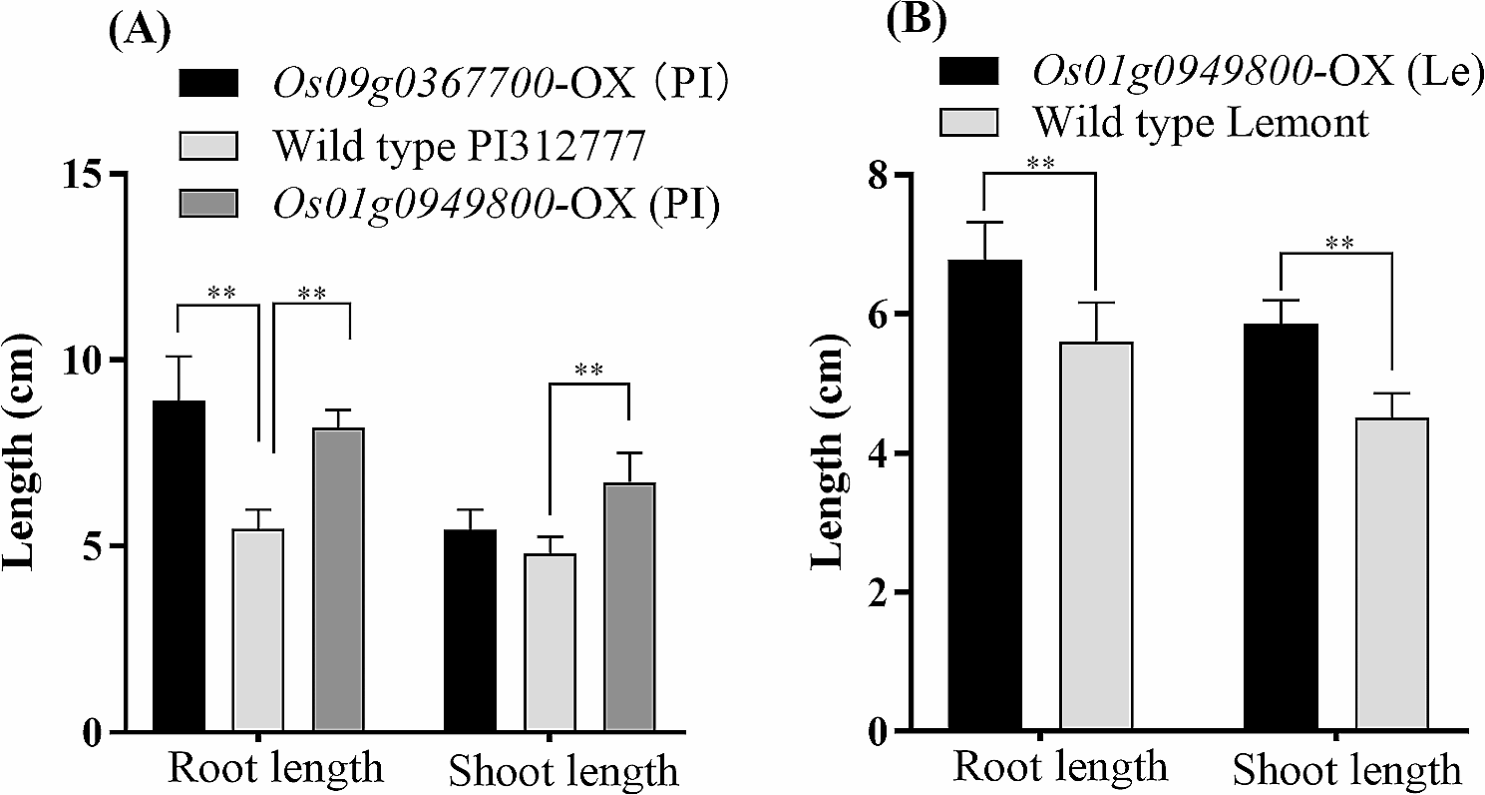
Growth effect of DIMBOA on the *GST*-OX and wild type lines. Germinated seeds of PI312777 (**A**) and Lemont (**B**) and their *GST*-OX lines were soaked in 0.05 mM DIMBOA. After 10 days, rice root and shoot lengths from the *GST*-OX and wild-type groups were measured. Error bars indicate standard deviation. Significant differences between the *GST*-OX line and its wild type are indicated by double asterisks (*p* < 0.01) according to Student’s t test

To further validate the function of GST in the regulation of rice tolerance to DIMBOA, we overexpressed *Os01g0949800* and *Os09g0367700* in PI312777 and *Os01g0949800* in Lemont (Fig. S4). Evaluation of the *GST*-OX rice tolerance to DIMBOA showed that increased *Os01g0949800* and *Os09g0367700* expression in PI312777 facilitated tolerance, and the root and shoot lengths of *Os01g0949800*-OX transgenic PI312777 were significantly longer than those of WT PI312777 when these two rice lines were treated with 0.05 mM DIMBOA (Fig. 7A). Overexpression of *Os01g0949800* in Lemont also resulted in enhanced rice tolerance to DIMBOA toxicity, and significantly longer root and shoot lengths were observed in *Os01g0949800*-OX transgenic Lemont than in its WT. In addition, the root length of *Os09g0367700*-OX transgenic PI312777 was also significantly longer than that of its WT (Fig. 7B).

### Protein regulatory networks of GST

**Fig. 8:**
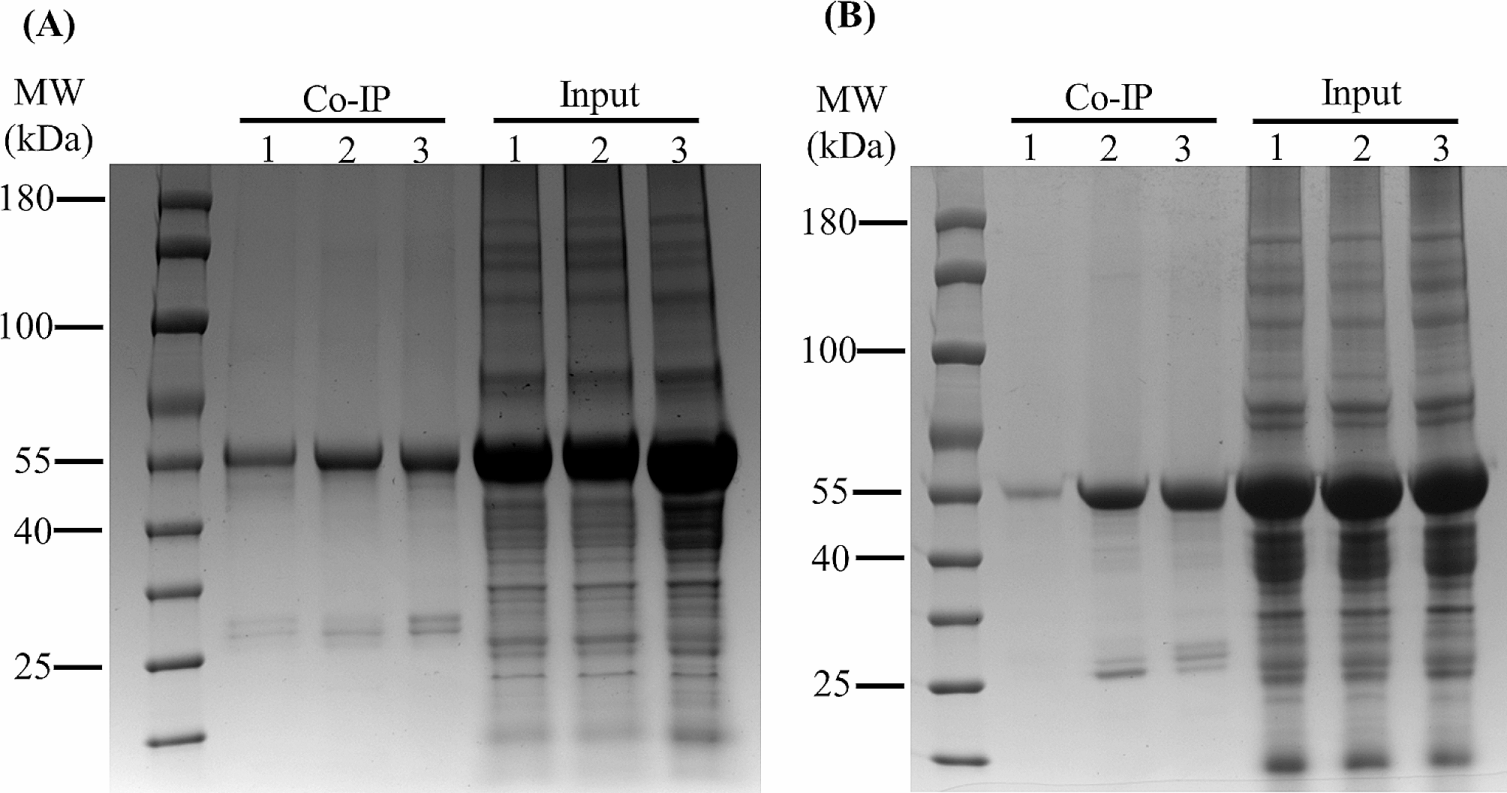
GST-interacting proteins from *GST*-OX transgenic rice. Proteins from the leaves of *Os01g0949800*-OX PI312777 (Lane 1), *Os09g0367700*-OX PI312777 (Lane 2) and *Os01g0949800*-OX Lemont (Lane 3) were extracted and incubated with GFP-Trap agarose beads to precipitate the proteins interacting with GST (Os01g0949800 and Os09g0367700). These rice lines were placed into two groups, without (**A**) and with DIMBOA treatment (**B**). The bands with a MW of approximately 55 kDa in the Input lanes represent GST protein fused with eYFP. Other bands in other lanes are the proteins interacting with GST

Proteins that interacted with GST were separated and identified (Fig. 8), and the results showed that the interacting proteins of GST (Os09g0367700) included two glutathione transferases, as well as thioredoxin-dependent peroxiredoxin, glutamate-1-semialdehyde 2,1-aminomutase, quinol cytochrome *c* reductase, protein-methionine-S-oxide reductase, serine/threonine-protein kinase SNT7, lipoxygenase, (S)-2-hydroxy-acid oxidase, cysteine desulfurase, and chloroplast-associated proteins (Table 1, Dataset S2). These proteins were enriched in metabolic pathways, secondary metabolite synthesis, carbon metabolism, amino acid synthesis, and glutathione metabolism (Fig. S5A).

**Table 1:** Proteins interacting with GST (Os09g0367700 and Os01g0949800) in rice

Accession	Description	Coverage	Peptides	PSMs
**Proteins interacting with GST (Os09g0367700)**				
Q5K3B1	Ribulose bisphosphate carboxylase large chain (Fragment)	54.41176471	23	93
Q7G7F8	Os10g0530500 protein	75.96566524	21	174
Q339G9	Ribulose bisphosphate carboxylase large chain	53.03867403	20	86
H2KVX3	Ribulose bisphosphate carboxylase/oxygenase activase, chloroplast, putative, expressed	58.54341737	19	44
A0A0E0HGC5	Phosphoglycerate kinase	49.07597536	19	38
A0A0E0HIC8	Uncharacterized protein	53.1120332	17	82
Q7 × 8A1	Glyceraldehyde-3-phosphate dehydrogenase	46.76616915	17	57
E9KIN8	ATP synthase subunit alpha, chloroplastic	39.58724203	17	23
A0A0E0HIB8	Uncharacterized protein	52.26337449	15	44
A0A0E0FK45	Magnesium-protoporphyrin IX monomethyl ester (oxidative) cyclase	33.25791855	15	16
Q9SNK3	Glyceraldehyde-3-phosphate dehydrogenase	33.33333333	15	40
B8AEQ9	Elongation factor Tu	39.13894325	15	31
A3BFU9	Os07g0108300 protein	40.20618557	14	23
B8AKV8	Uncharacterized protein	34.9009901	13	15
A0A0P0XX30	Os10g0530500 protein	67.2	13	102
A3AV14	Glyceraldehyde-3-phosphate dehydrogenase	45.71428571	13	30
A0A0E0HLA5	Geranylgeranyl reductase	41.03671706	13	17
A0A0E0HUY0	Uncharacterized protein	33.55408389	12	16
A0A0E0IUD7	Uncharacterized protein	32.35294118	11	13
A0A0P0WIQ8	Os05g0164100 protein	17.59379043	11	12
B8A962	Uncharacterized protein	40.69264069	10	29
Q0J128	Os09g0467200 protein	45.29147982	10	20
Q0J294	Os09g0367700 protein (Fragment)	41.63090129	10	69
B8AF09	Glyceraldehyde-3-phosphate dehydrogenase	35.6741573	10	23
A0A0E0H6Y4	Glutamine synthetase	29.66292135	10	14
A0A0E0IF06	Glutamate-1-semialdehyde 2,1-aminomutase	32.42677824	10	12
Q10QZ4	Elongation factor 1-alpha	34.07572383	10	19
Q0JKY8	Carbonic anhydrase (Fragment)	41.63701068	10	24
B8BA11	Uncharacterized protein	33.56643357	9	11
A0A0E0IVE3	Uncharacterized protein	10.76023392	9	25
A0A0E0HK09	Uncharacterized protein	20.63227953	9	10
P46265	Tubulin beta-5 chain	25.05592841	9	11
A0A0E0ITA5	Tubulin beta chain	25.16853933	9	11
A0A0E0HC00	NAD(P)-bd_dom domain-containing protein	22.52747253	9	9
A2YQT7	Glyceraldehyde-3-phosphate dehydrogenase, cytosolic	39.46587537	9	19
Q6WSC2	Glutathione S-transferase	30.90128755	9	20
A0A0E0IS47	Epimerase domain-containing protein	28.04232804	9	13
Q53N83	Chlorophyll a-b binding protein, chloroplastic	44.87632509	9	24
A0A5S6R9N4	ATP synthase subunit beta	26.90763052	9	11
A0A0E0G138	ATP synthase subunit alpha	17.82682513	9	12
A0A0E0IE06	Alanine–glyoxylate transaminase	31.39534884	9	10
A0A0E0GUL1	(S)-2-hydroxy-acid oxidase	15.82952816	9	9
**Proteins interacting with GST (Os01g0949800)**				
Q0JG12	Os01g0949800 protein	69.26406926	28	371
Q5K3B1	Ribulose bisphosphate carboxylase large chain	55.88235294	27	74
A0A0E0FYT0	Uncharacterized protein	18.43657817	25	330
P93431	Ribulose bisphosphate carboxylase/oxygenase activase, chloroplastic	61.80257511	25	77
Q7 × 8A1	Glyceraldehyde-3-phosphate dehydrogenase	66.16915423	24	85
Q339G9	Ribulose bisphosphate carboxylase large chain	54.97237569	23	67
A0A0E0HGC5	Phosphoglycerate kinase	57.90554415	22	55
Q9SNK3	Glyceraldehyde-3-phosphate dehydrogenase	51.12612613	22	63
A0A0E0HIC8	Uncharacterized protein	74.27385892	21	62
A3BFU9	Os07g0108300 protein	53.19587629	21	53
B8AEQ9	Elongation factor Tu	53.42465753	20	41
E9KIN8	ATP synthase subunit alpha, chloroplastic	40.15009381	20	26
Q7G7F8	Os10g0530500 protein	59.65665236	19	77
A0A0P0 × 1V6	Os07g0108300 protein (Fragment)	55.14874142	18	42
P0C2Z7	ATP synthase subunit beta, chloroplastic	53.81526104	17	26
A0A0E0H6Y4	Glutamine synthetase	39.3258427	16	25
A0A0E0IVK6	Uncharacterized protein	23.04526749	15	203
B8AW41	NAD(P)-bd_dom domain-containing protein	40.4040404	15	18
A0A0E0HLA5	Geranylgeranyl reductase	45.35637149	15	16
A3AV14	Glyceraldehyde-3-phosphate dehydrogenase	51.94805195	14	25
A0A0E0HIB8	Uncharacterized protein	52.26337449	13	18
P46265	Tubulin beta-5 chain	37.36017897	13	16
A0A0E0ITA5	Tubulin beta chain	37.52808989	13	16
A0A0E0HHS1	Protein kinase domain-containing protein	29.05982906	13	14
A0A0P0XX30	Os10g0530500 protein	72.8	13	51
A0A0E0FK45	Magnesium-protoporphyrin IX monomethyl ester (oxidative) cyclase	31.67420814	13	15
A2YQT7	Glyceraldehyde-3-phosphate dehydrogenase, cytosolic	64.09495549	13	22
A2XVY3	Elongation factor G, chloroplastic	23.51421189	13	16
B8A8L8	Uncharacterized protein	45.34534535	12	14
A0A0E0IUD7	Uncharacterized protein	35.74660633	12	16
Q7Y1F0	Serine hydroxymethyltransferase	37.70197487	12	14
Q0J128	Os09g0467200 protein	48.87892377	12	33
A0A0E0IF06	Glutamate-1-semialdehyde 2,1-aminomutase	34.10041841	12	14
A0A0E0GWL1	Elongation factor Tu	37.08609272	12	16
A2Y0Q8	CBM20 domain-containing protein	21.76165803	12	12
A0A0E0IVE3	Uncharacterized protein	14.15204678	11	17
B8AF09	Glyceraldehyde-3-phosphate dehydrogenase	43.25842697	11	18
B8B936	Glutamate-1-semialdehyde 2,1-aminomutase	33.11688312	11	13
Q0JHF8	Fructose-1,6-bisphosphatase, cytosolic	33.92330383	11	13
Q10QZ4	Elongation factor 1-alpha	43.65256125	11	24
E9KIQ1	Cytochrome f	41.92546584	11	14
Q0JKY8	Carbonic anhydrase (Fragment)	42.34875445	11	25
P0C520	ATP synthase subunit alpha, mitochondrial	27.11198428	11	16
A0A0E0IZ66	Aldehyde dehydrogenase	27.32793522	11	13
A0A0E0IE06	Alanine-glyoxylate transaminase	40.46511628	11	14
P0C539	Actin-2	45.88859416	11	17
A0A218KL39	Actin-1	45.88859416	11	20
A0A0E0HWH9	(S)-2-hydroxy-acid oxidase	40.92140921	11	16
A0A0E0GUL1	(S)-2-hydroxy-acid oxidase	19.7869102	11	14
B9F4B5	Uncharacterized protein	26.51356994	10	11
A0A0E0IYB0	Uncharacterized protein	24.70817121	10	10
A0A0E0IYI4	Uncharacterized protein	40.15345269	10	19
A0A0E0FW49	Uncharacterized protein	40.69148936	10	15
A0A0E0HUY0	Uncharacterized protein	30.24282561	10	15
A2YIS2	Ribos_L4_asso_C domain-containing protein	37.5308642	10	11
Q8GRU9	Phosphoribulokinase	31.51364764	10	16
Q0J294	Os09g0367700 protein (Fragment)	41.63090129	10	29
Q94DL4	Os01g0964133 protein	40.31830239	10	18
A0A0E0IFL1	NmrA domain-containing protein	27.4611399	10	10
A0A0E0HVM5	Component of oligomeric Golgi complex 7	13.76744186	10	11

GST (Os01g0949800) also interacted with several glutathione transferases, together with geranylgeranyl reductase, thioredoxin-dependent peroxiredoxin, S-(hydroxymethyl) glutathione dehydrogenase, serine hydroxymethyltransferase, peroxidase, *S*-adenosylmethionine synthase, peroxisomal membrane protein 11 − 1, sulfate adenylyltransferase, protein disulfide-isomerase, cytochrome *b-c1* complex subunit, cytochrome f, and other proteins that interacted with Os09g0367700 (Table 1, Dataset S3). These proteins were enriched in metabolic pathways, secondary metabolite synthesis, carbon metabolism, amino acid synthesis, glutathione metabolism, and oxidative phosphorylation pathways (Fig. S5B).

### Transcript level of proteins interacting with GST

The transcript levels of GST-interacting protein-coding genes in PI312777 and Lemont, including L-ascorbate peroxidase 4 (*Os08g0549100*), phosphoglycerate kinase (*Os05g0496200*), fructose-6-phosphate-2-kinase (*Os05g0164100*), GST (*Os10g0530500*), peroxidase precursor (*Os02g0240300*), dehydrogenase (*Os02g0815500*), aminotransferase (*Os07g0108300*), and glutaredoxin-dependent peroxiredoxin (Peroxiredoxin-2E-2, *Os02g0192700*) were determined. Among these genes, the expression of *Os02g0240300* and *Os10g0530500* was upregulated in PI312777 when the rice was treated with 0.02 mM-0.10 mM DIMBOA. The fold changes of these genes were found to be the highest under 0.10 mM DIMBOA treatment, and *Os10g0530500* presented a 10.8-fold upregulation in comparison with the control group.

**Fig. 9:**
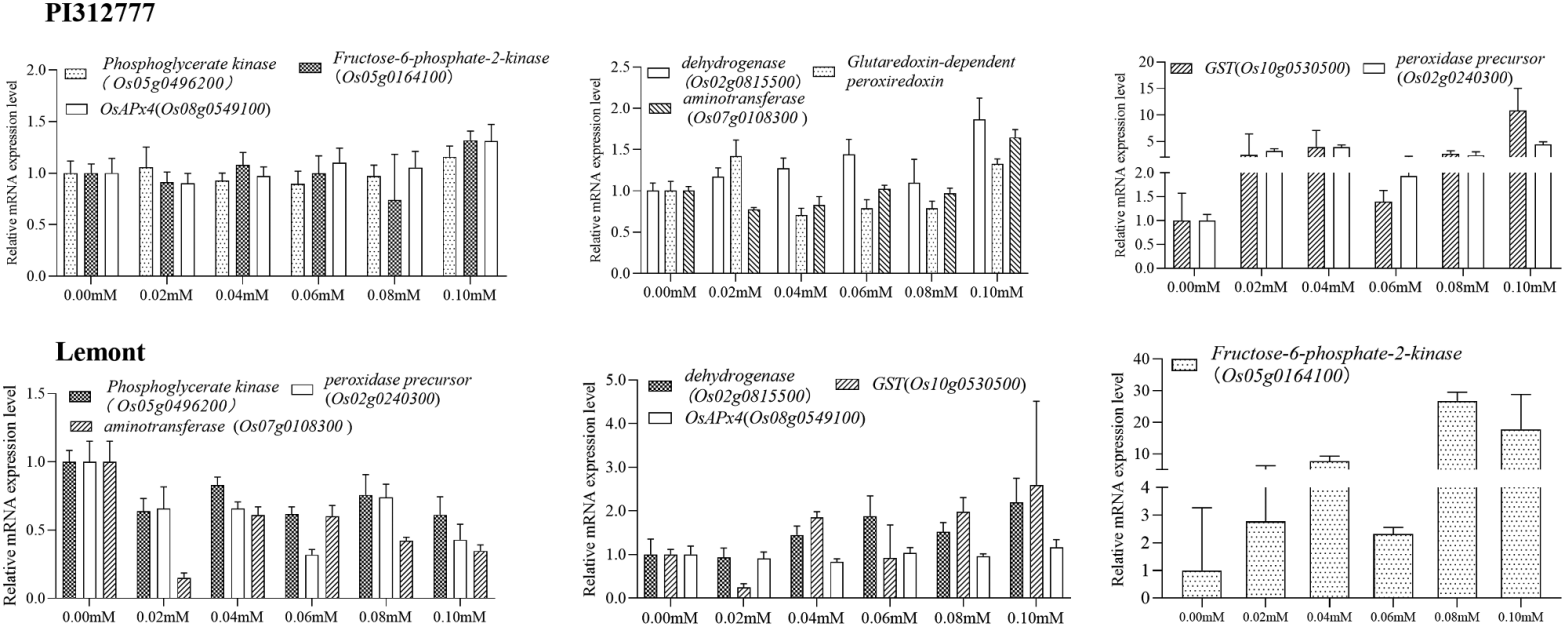
Gene transcript levels of GST-interacting proteins in the roots of PI312777 and Lemont under DIMBOA treatment. The transcript levels of GST (Os01g0949800, Os09g0367700)-interacting proteins in PI312777 and Lemont cultivars treated with concentrations ranging from 0.02 mM to 0.10 mM were determined and compared with the corresponding control groups

For Lemont, *Os10g0530500* and *Os02g0815500* were mostly upregulated in these treatment groups, and *Os05g0164100* was upregulated in all treatment groups (Fig. 9).

## Discussion

Weeds are unavoidable limiting factors in crop production because they release inhibitory compounds that can disturb crop growth and act as competitors for resources with crops [10, 20]. Among them, barnyard grass (*Echinochloa crus-galli*) is one of the most noxious weeds in paddy fields, seriously affecting the yield and quality of rice. Currently, chemical herbicides are mainly used to control *Echinochloa crus-galli* [21]. Studies have shown that within a certain dose range, allelopathic rice exhibits an enhancement of allelopathic potential when subjected to an increased exogenous addition of barnyard grass extract. However, if the dose of weed extract is too high, it inhibits rice growth [22]. DIMBOA, a root exudate, has been reported as a severely toxic compound that interferes with rice growth. A gene cluster for DIMBOA biosynthesis has been identified in the genome of barnyard grass but is absent in the rice genome [10]. Thus, DIMBOA is specifically released from the roots of barnyard grass to suppress rice growth in paddy fields during rice-barnyard grass competition. Therefore, the tolerance and detoxification capacity of rice towards DIMBOA are vital for the survival of the crop but may vary across different rice accessions.

In this study, the allelopathic rice PI312777 and nonallelopathic rice Lemont showed different levels of tolerance to exogenous DIMBOA. Tolerance in PI312777 was inducible under an appropriate concentration of DIMBOA (≤ 0.06 mM in this study), while Lemont showed more consistent tolerance, with no significant effect observed on Lemont rice growth when the exogenous DIMBOA concentration was lower than 0.08 mM. The results suggest that nonallelopathic rice lacks strong “allelochemicals weapon” to suppress barnyard grass growth [4, 6, 23], nevertheless, this rice variety has evolved to be more tolerant to DIMBOA exudates from barnyard grass. This characteristic benefits the nonallelopathic rice in its survival within the natural rice and weed system.

According to the iTRAQ quantitative proteomics results in rice, GST proteins participating in glutathione metabolism were speculated to be relevant to regulating rice tolerance to DIMBOA. In barnyard grass, genome sequencing and gene annotation revealed that a large number of genes that function in detoxification are distributed in the barnyard grass genome, such as GST and cytochrome P450 monooxygenase (CYP450), and these genes likely play positive roles in the detoxification of rice allelochemicals [24]. GSTs are important defence enzymes that are mainly cytosolic proteins, and these enzymes usually play active roles in the form of dimers to detoxify xenobiotics and reduce oxidative damage and endogenous metabolism, which results in diverse catalytic and noncatalytic activities in metabolism [25]. In the plant response to environmental stress factors, GSTs function in protecting cells from damage by invading organisms, heavy metals, oxidative stress and pathogens [26–28]. As a multifunctional hypergene family, there are at least 79 genes encoding GST in rice [29], and plant *GST* genes are divided into fourteen classes, including tau (U), phi (F), lambda (L), dehydroascorbate reductase (DHAR), theta (T), and zeta (Z), depending on their nucleotide composition [30]. It was documented that phi GSTs in black grass (*Alopecurus myosuroides*) and annual rye grass (*Lolium rigidum*) can participate in the detoxification metabolism of herbicides [31].

Two GSTs showed expression changes in both PI312777 and Lemont treated with DIMBOA, and these differentially expressed GSTs resulted in significant enrichment in glutathione metabolism, indicating the tight correlation of this pathway with rice tolerance. Several of these enzymes showed increased fold changes with an increasing DIMBOA dosage, suggesting a positive reaction to the tolerance to DIMBOA. In particular, the transcript levels of *Os01g0949800* and *Os09g0367700* increased in rice under DIMBOA treatment, especially the expression of *Os09g0367700* in DIMBOA-treated PI312777. The enhanced expression of GST leads to increased enzyme activity during detoxification. In PI312777, lower GST activity was found in the roots without DIMBOA treatment; however, GST activity was slightly induced by DIMBOA, resulting in a higher tolerant ability. In contrast to PI312777, the nonallelopathic rice Lemont has a higher GST activity in the roots under natural conditions, and the activity was more stable under low concentrations of DIMBOA, which enabled the rice to tolerate the toxicity of DIMBOA.

A total of 29 DEPs from PI312777 treated with 0.06 mM DIMBOA were closely related to each other, indicating the systemic response of PI312777 to DIMBOA treatment and the cross-talk of the defence network in rice. Here, we overexpressed *Os01g0949800* and *Os09g0367700* in PI312777 and *Os01g0949800* in Lemont. The results showed that increasing the level of *GST* gene expression in rice enhanced its tolerance to DIMBOA. The root and shoot lengths of *Os01g0949800*-OX transgenic rice were both significantly longer than those of their corresponding WT when treated with 0.05 mM DIMBOA. The results indicated that the *GST*-OX transgenic rice lines had a higher tolerance to relieve the toxic effects of DIMBOA, which also validates the role of GST in the transformation or degradation of xenobiotics. Overexpression of the *OsGSTL1* gene in rice enhanced tolerance to chlorosulfuron and glyphosate in rice seedlings [32].

During the process of GST activation, Os01g0949800 (GST) interacted with other GSTs, as well as L-ascorbate peroxidase, peroxisomal membrane protein 11 − 1, protein disulfide-isomerase, and many proteins involved in porphyrin and chlorophyll metabolism, oxidative phosphorylation, the pentose phosphate pathway, and the main pathways from plant photosynthesis and respiration. These results suggested that the detoxification process was associated with energy metabolism and substrate metabolite transformation. The increased transcriptional expression of these interacting proteins in PI312777 was consistent with the expression pattern of the target GST, and the results indicated the cooperation of these particular proteins.

In *Arabidopsis*, *O*-glucosylation of allelochemical benzoxazolin-2(3 H)-one (BOA) was identified as the predominant detoxification to the chemical [33]. Meanwhile, upregulation of UDP-glucosyltransferase would also facilitate rice detoxification of DIMBOA.

In this study, the different responses of the allelopathic rice PI312777 and nonallelopathic rice Lemont to DIMBOA were mainly dependent on the basal and inducible levels of GST activity. The sensitivity to the induction of GST activity in PI312777 enables the plant to effectively react to xenobiotics, which allows allelopathic rice to lessen the toxic effects on cellular metabolism and to maintain plant growth. However, in the nonallelopathic rice Lemont, a low level of induction but high basal GST activity helped the rice line tolerate the toxicity of DIMBOA, which prevented major reductions in plant growth. The different strategies of allelopathic rice and nonallelopathic rice in the tolerance of the barnyard grass exudate DIMBOA provide additional directions for breeding weed-tolerant rice.

## Methods

### Plant materials

The allelopathic rice accession PI312777 and nonallelopathic accession Lemont (*Oryza sativa* japonica) were used as the study materials [34, 35]; PI312777 seeds were obtained from the International Rice Research Institute, The Philippines. Lemont seeds were obtained from Texas A&M University Agricultural Research and Extension Center, Beaumont, Texas.

### Evaluation of the DIMBOA tolerance of PI31277 and Lemont

The seeds of PI312777 and Lemont were disinfected with 2.5% sodium hypochlorite (NaClO) solution for 30 min, washed with sterile water to clear the NaClO residue on the seeds, and soaked overnight at 30 °C in a thermostatic incubator to promote germination. DIMBOA (Toronto Research Chemicals Inc., Canada) was dissolved in the methanol to prepare a 100 mM storage solution. This storage solution was then further diluted with sterile water to create different concentrations of a working solution. The treatment groups were prepared with a series of concentration gradients: 0.02 mM, 0.04 mM, 0.06 mM, 0.08 mM, and 0.10 mM DIMBOA. The experiment was conducted in six-well plates, with each well containing 10 ml of solution. In each well, 10 germinated seeds of either PI312777 or Lemont rice were soaked. The corresponding control groups for PI312777 and Lemont consisted of germinated seeds cultured in sterile water with 10 µL of methanol solvent. Both the treatment groups and the control groups were tested in triplicate. The plates were placed in an artificial climate chamber (MGC-300 H, Shanghai Yiheng Scientific Instrument Co., LTD) set at a temperature of 26 °C, humidity of 85%, light intensity of 12,000 lx, and a light/dark cycle of 14 h of light and 10 h of darkness. After 4 days of culture, the root length and plant height of the control group and each treatment group were measured.

### Quantitative proteomics analysis of DIMBOA-treated rice seedlings

Seeds of PI312777 and Lemont were disinfected and germinated following the description above. The germinated seeds were sown in a net and floated in a 1 L rectangular container fully filled with a 1/2 concentration of optimized rice hydroponic nutrient solution [36, 37]. The containers were placed in the climate chamber at 28 °C for 14 h/22°C for 10 h for 10 days (when the rice seedlings grew to the 3-leaf stage). Seedlings of PI312777 and Lemont were cultivated in complete hydroponic nutrient solution containing 0.04 mM and 0.06 mM DIMBOA, respectively. Seedlings of both cultivars grown in the nutrient solution without DIMBOA were used as controls. Rice roots from the treatment and control groups were independently sampled after treatment for 7 days and kept in liquid nitrogen.

### Rice root protein extraction

One gram of rice roots with 5% PVPP were ground into powder in liquid nitrogen and resuspended in Tris-saturated phenol (Beijing Solarbio Science & Technology Co., Ltd.) at a 5:1 ratio. The mixture was then vortexed at 4 °C for 15 min. After centrifugation at 25,000 g and 4 °C for 20 min, the supernatant was treated by adding 5 volumes of 0.1 M ammonium acetate/methanol with 10 mM DTT to precipitate proteins at -20 °C for 2 h, and then centrifugation at 25,000 *g* and 4 °C for 15 min. The precipitation step was repeated twice. After that, pre-cooled acetone with 10 mM DTT was added in the precipitation and fully mixed, and kept at at -20 °C for 2 h, the supernatant was discarded after centrifugation at 25,000 g and 4 °C for 20 min, and the precipitation step was repeated once. The proteins were air dried at 4 °C and resuspended in lysis buffer (8 M urea and 40 mM Tris-HCl containing 1 mM PMSF, 2 mM EDTA, 10 mM DTT and 1×Protease Inhibitor Cocktail, pH 8.5) again and ultrasonicated on ice for 5 min (sonication for 2 s/pause for 3 s) to improve protein dissolution. After centrifugation, DTT (10 mM final concentration, FC) was added to the supernatant, which was incubated at 56 °C for 1 h for reduction and then alkylated with 55 mM iodoacetamide (IAM) in the dark at room temperature for 45 min. Five volumes of acetone was used to precipitate proteins at -20 °C overnight. Lysis buffer was used to dissolve the proteins with the help of sonication on ice for 5 min (2 s/3 sec). Proteins were quantitated with a Bradford assay and separated and qualified by SDS‒PAGE for the Isobaric Tags for Relative and Absolute Quantitation (iTRAQ) proteomic analysis. The experiment was conducted by BGI Co., LTD (Shenzhen, China) [38, 39]. The protocol details are available in Supplementary Dataset 1.

### Bioinformatics analysis of differentially expressed proteins and their related pathways

The raw MS/MS data were converted into MGF format by the corresponding tool, and the exported MGF files were searched against the database described above via the local Mascot server. In addition, quality control (QC) was performed to determine if a reanalysis step was needed. Automated software named Iquant was applied to the quantification results of the proteins [40]. All the proteins with a false discovery rate (FDR) less than 1% were subjected to downstream analysis, including Gene Ontology (GO), COG/KOG and pathway analyses. Proteins with an expression fold change between the treatment and control of more than 1.5-fold were regarded as differentially expressed proteins (DEPs). Furthermore, analysis of the GO enrichment and KEGG pathway enrichment of DEPs was performed to investigate their relevant pathways.

### Determination of glutathione S-transferase (GST) gene expression

Total RNA from the roots of PI312777 and Lemont treated with 0.02 mM, 0.04 mM, 0.06 mM, 0.08 mM and 0.10 mM DIMBOA and their control groups was extracted by using the TRIzol method. TransScript OneStep gDNA Removal and cDNA Synthesis SuperMix (TransGen Biotech Co., Ltd., Beijing, China) were used to reverse transcribe the mRNA into cDNA. The transcript levels of two genes, *Os01g0949800* and *Os09g0367700*, which both encode *glutathione S-transferase* (*GST*), were determined in the DIMBOA-treated rice and the control group according to quantitative PCR (qPCR) with gene-specific primers (*Os01g0949800*, F: 5 ‘-GGGAGTGGCTTACGAGTTCA-3’, R: 5’-TCGGTGGGTAGGATGGGAC-3’; *Os09g0367700*, F: 5’-CTCGTCATCCTCGAGTACATC-3’, R: 5’- ACAGCTTCTTGTC GACGTAG-3’). The *β-actin* gene (F: 5 ‘-CTTCATAGGAATGGAAGCTGCGGGTA-3’, 5 ‘-CGACCACCTTGATCTTCATGCTGCTA-3’) was used as an internal reference for qPCR detection. The reaction mix for gene amplification was prepared following the TransStart Green qPCR SuperMix Kit (TransGen Biotech Co., Ltd., Beijing, China). The PCR program was set as follows: initial denaturation at 94 ℃ for 30 s, denaturation at 94 ℃ for 5 s, annealing at 58 ℃ for 15 s, and extension at 72 ℃ for 10 s. The reaction from denaturation to extension was conducted for 42 cycles; thereafter, the temperature rose from 60 ℃ to 95 ℃ at a rate of 0.2 ℃/s. The reaction was conducted in a realplex^4^ Eppendorf. The threshold value (CT value) was automatically recorded by Eppendorf instrument software, and the relative gene expression level was calculated by the $2^{-\Delta\Delta{\rm Ct}}$ method [41] and plotted in GraphPad Prism 5.

### Determination of GST activity

The roots of P312777 and Lemont treated with 0.02 mM, 0.04 mM, 06 mM, 0.08 mM and 0.10 mM DIMBOA and their control groups were used to extract the crude enzyme. Both the treatment group and the control group were tested in triplicate, and 0.1 g root tissue of each replicate was used for enzyme extraction. The GST activity of PI312777 and Lemont roots treated with different DIMBOA concentrations was determined by using the Glutathione *S*-transferase (GST) Activity Assay Kit (Beijing Solarbio Science & Technology Co., Ltd.).

### Overexpression of GST in rice

The CDS of *OsGST* (*Os01g0949800* and *Os09g0367700*) was amplified and inserted into a modified pCambia2300 vector (with the 35S promoter and *eYFP* gene fused to the target gene) to construct the *eYFP* recombinant vector for rice transformation [14]. *Agrobacterium tumefaciens* (EHA105)-mediated transformation was performed on calli of PI312777 and Lemont [42, 43]. Resistant calli were screened by 80µg/ml geneticin G418, and putatively transformed tissue was then differentiated and dedifferentiated to generate T_0_ transgenic rice seedlings. Fragments containing both *GST* and eYFP were amplified for sequencing (*Os01g0949800*-Transdetect-F: 5’-AAGGAGACGAAGGAGAACCTGG-3’; *Os01g0949800*-Transdetect-R: 5’-GTGGCGGATCTTGAAGTTCACC-3’; *Os09g0367700*-Transdetect-F: 5’-AGAAGCTGTTCGACTGCCAGAC-3’; *Os09g0367700*-Transdetect-R: 5’-CTCCTTGAAGTCGATGCCCTTC-3’) to identify positive transgenic lines. The positive *GST* overexpression (*GST*-OX) T_0_ transgenic lines were then retained for harvesting the T_1_ transgenic seeds.

### Evaluation of the DIMBOA tolerance of GST-OX lines

The positive *GST*-OX T_1_ transgenic PI312777 and Lemont rice seeds were disinfected and germinated following the steps described above. Uniformly germinated transgenic and wild-type (WT) rice seeds were soaked in sterilized water with exogenous concentrations of 0.05 mM DIMBOA as the treatment group. The germinated transgenic and WT seeds cultured in sterile water without DIMBOA were used as the control groups. The experiment was also conducted in six-well plates, and both the treatment group and the control group were tested in triplicate and placed in an artificial climate chamber at 26 °C and 85% humidity. After 7 days of culture, the root length and plant height of the control group and the treated rice were measured.

### Identification of the proteins interacting with GST

Natural leaf proteins were extracted from a T_1_ generation of *GST*-overexpression transgenic rice by using Pi-IP buffer (50 mM Tris pH 7.5; 150 mM NaCl; 1 mM EDTA; 1% Triton X-100; 1 mM PMSF; 1× Complete cocktail, Roche; 10 µM MG132) and incubated with GFP-Trap agarose (Chromotek) to collect putative GST-interacting proteins. The protocol details are available in Supplementary Dataset 1. These proteins were identified by using a Q-Exactive Plus mass spectrometer (Thermo Fisher Scientific).

### Quantitative PCR to determine gene expression levels

The transcript levels of GST-interacting proteins in PI312777 and Lemont under 0.02 mM, 0.04 mM, 0.06 mM, 0.08 mM and 0.10 mM DIMBOA treatment were determined by qPCR. The transcript levels of the promoter binding proteins were also determined to evaluate their regulatory capacity on *GST* gene expression. The protocol for qPCR determination was described above.

### Statistical methods

SPSS 26.0 was utilized for the statistical analysis of the data. The data were presented as mean ± standard error (SE) values obtained from three replicates for each experiment or determination. The variance in the mean values of root length, shoot length, and GST activity among the different concentrations of DIMBOA treatment on rice was analyzed using one-way ANOVA, followed by Tukey’s honestly significant difference (HSD) test at a significance level of *p* < 0.05.

### Electronic supplementary material

Below is the link to the electronic supplementary material.

**Supplementary Material 1: Fig. S1.** Phenotype of PI312777 and Lemont seedlings after treatment with DIMBOA. Bar, 5cm

**Supplementary Material 2: Fig. S2.** Amounts of differentially expressed proteins from the roots of PI312777 and Lemont after treatment with DIMBOA

**Supplementary Material 3: Fig. S3.** Predicted protein‒protein interactions among differentially expressed proteins from DIMBOA-treated PI312777 and the control group. Red nodes represent upregulated proteins; blue nodes represent downregulated proteins

**Supplementary Material 4: Fig. S4.** PCR amplification of the DNA fragment of GST-eYFP fused gene from Os09g0367700-OX and Os01g0949800-OX line for positive transgenic lines identification

**Supplementary Material 5: Fig. S5.** KEGG enrichment of the proteins interacting with Os09g0367700 and Os01g0949800

**Supplementary Material 6: Dataset S1.** Protocol details of iTRAQ proteomics and Co-IP

**Supplementary Material 7: Dataset S2.** Mass spectrum identification of proteins interacting with Os09g0367700 in PI312777

**Supplementary Material 8: Dataset S3.** Mass spectrum identification of proteins interacting with Os01g0949800 in Lemont

**Supplementary Material 9: Electronic Supplementary Material 1.** Full length gel presents PCR amplification of the DNA fragment of GST-eYFP fused gene from Os09g0367700-OX and Os01g0949800-OX transgenic PI312777 lines

**Supplementary Material 10: Electronic Supplementary Material 2.** Full length gel presents PCR amplification of the DNA fragment of GST-eYFP fused gene from Os01g0949800-OX transgenic Lemont line

**Supplementary Material 11: Electronic Supplementary Material 3.** Full length gel presents GST-interacting proteins from GST-OX transgenic rice without DIMBOA treatment

**Supplementary Material 12: Electronic Supplementary Material 4.** Full length gel presents GST-interacting proteins from GST-OX transgenic rice with DIMBOA treatment

**Supplementary Material 13**

## Data Availability

All data generated during this study are included in this published article and its supplementary information files, and the raw data of iTRAQ quantitative proteomics generated in this study have been deposited to the ProteomeXchange Consortium (http://proteomecentral.proteomexchange.org) via the iProX partner repository [44, 45] with the dataset identifier PXD041754.

## Abbreviations

DEPs: differentially expressed proteins
DIMBOA: 2,4-dihydroxy-7-methoxy-2 H-1,4-benzoxazin-3(4 *H*)-one
GST: glutathione *S*-transferase
iTRAQ: Isobaric Tags for Relative and Absolute Quantitation
qPCR: quantitative PCR
