# Supplementary material for: Glutathione S-transferase activity facilitates rice tolerance to the barnyard grass root exudate DIMBOA
Source: BMC Plant Biol. 2024 Feb 17;24:117. doi: 10.1186/s12870-024-04802-5 (PMC10874003; doi:10.1186/s12870-024-04802-5)
Supplement: Supplementary file 1 — Supplementary Material 1: Fig. S1. Phenotype of PI312777 and Lemont seedlings after treatment with DIMBOA. Bar, 5cm [file 12870_2024_4802_MOESM1_ESM.docx]

**Protocols of Isobaric Tags for Relative and Absolute Quantitation (iTRAQ) proteomics**

1. **Protein Quantitation with Bradford assay**

Add 0, 2, 4, 6, 8, 10, 12, 14, 16 and 18μl BSA (0.2μg/μl) solution separately to a 96-well plate, and then add 20, 18, 16, 14, 12, 10, 8, 6, 4 and 2 μl pure water to the corresponding wells. Also prepare serial dilutions (20μl each well) of the unknown sample to be measured. Add 180μl of Coomassie Blue to each well and mix. Read the absorbance of each standard and sample well at 595 nm. Each sample has at least two duplicates. Plot the absorbance of the standards vs. their concentration. Compute the extinction coefficient and calculate the concentrations of the rice samples.

1. **SDS-PAGE**

Mix 30 μg proteins with loading buffer in centrifuge tube and heat them at 95℃ for 5 minutes. Then, centrifuge it at 25000g for 5 minutes and load the supernatant to sample holes in 12% polyacrylamide gel. Run SDS-PAGE in constant voltage at 120V for 120 minutes. Once it finished, stain gel with Coomassie Blue for 2 hours, then add destaining solution(40% ethanol and 10% acetic acid) and put it on a shaker (exchange destaining solution for 3~5 times, 30 minutes a time).

1. **Protein Digestion**

The protein solution (100μg) with 8M urea was diluted 4 times with 100mM TEAB. Trypsin Gold (Promega, Madison, WI, USA) was used to digest the proteins with the ratio of protein : trypsin =40 : 1 at 37°C overnight. After trypsin digestion, peptides were desalted with a Strata X C18 column (Phenomenex) and vacuum-dried according to the manufacturer's protocol.

1. **Peptide Labeling**

The peptides were dissolved in 30μl 0.5M TEAB with vortexing. After the iTRAQ labeling reagents were recovered to ambient temperature, they were transferred and combined with proper samples. Peptide labeling was performed by iTRAQ Reagent 8-plex Kit according to the manufacturer's protocol. The labeled peptides with different reagents were combined and desalted with a Strata X C18 column (Phenomenex) and vacuum-dried according to the manufacturer's protocol.

1. **Peptide Fractionation**

The peptides were separated on a Shimadzu LC-20AB HPLC Pump system coupled with a high pH RP column. The peptides were reconstituted with buffer A (5% ACN, 95% H_2_O, adjust pH to 9.8 with ammonia) to 2 ml and loaded onto a column containing 5-μm particles (Phenomenex). The peptides are separated at a flow rate of 1 mL/min with a gradient of 5% buffer B (5% H_2_O, 95% ACN, adjust pH to 9.8 with ammonia) for 10 min, 5-35% buffer B for 40min, 35-95% buffer B for 1 min. The system is then maintained in 95% buffer B for 3 min and decreases to 5% within 1 min before equilibrating with 5% buffer B for 10 min. Elution is monitored by measuring absorbance at 214 nm, and fractions are collected every 1 min. The eluted peptides are pooled as 20 fractions and vacuum-dried.

1. **HPLC**

Each fraction was resuspended in buffer A (2% ACN, 0.1%FA) and centrifuged at 20,000g for 10 min. The supernatant was loaded on Thermo Scientific™ UltiMate™ 3000 UHPLC system equipped with a trap and an analytical column. The samples were loaded on a trap column at 5 μL/min for 8 min, and then eluted into the homemade nanocapillary C18 column (ID 75 μm x 25 cm, 3 μm particles) at a flow rate 300nl/min. The gradient of buffer B (98% ACN, 0.1% FA) was increased from 5% to 25% in 40 min, and then increased to 35% in 5 min, followed by 2 min linear gradient to 80%, then maintenance at 80% B for 2 min, and finally return to 5% in 1 min and equilibrated for 6 min.

1. **Mass Spectrometer Detection**

The peptides separated from nanoHPLC were subjected into the tandem mass spectrometry Q EXACTIVE HF X (Thermo Fisher Scientific, San Jose, CA) for DDA (data-dependent acquisition) detection by nano-electrospray ionization. The parameters for MS analysis are listed as following: electrospray voltage: 2.0 kV; precursor scan range: 350-1500 m/z at a resolution of 60,000 in Orbitrap; MS/MS fragment scan range: >100 m/z at a resolution of 15,000 in HCD mode; normalized collision energy setting: 30%; dynamic Exclusion time: 30 s; Automatic gain control (AGC) for full MS target and MS2 target: 3e6 and1e5, respectively; The number of MS/MS scans following one MS scan: 20 most abundant precursor ions above a threshold ion count of 10,000.

**Protocols of Co-Immunoprecipitation to isolate proteins interacted with GST**

1. Grind rice leaves in liquid N_2_ into fine powder.
2. Estimate volume of pulverized tissue powder (3 ml), add 2 times volume of the freshly prepared Pi-IP buffer (50 mM Tris, pH 7.5; 150 mM NaCl, 1 mM 0.5M EDTA; 1% Triton X-100, 1mM PMSF, 1×Complete cocktail, Roche; 10μM MG132).
3. Votex well and let the mixture thaw on ice or rotate at 4℃ for 15 min.
4. Centrifuge the samples at 4℃, 16,000×g, for 15 min in a centrifuge.
5. Pour supernatant into a new tube, using sterile Miracloth to filter the supernatant. Repeat the centrifugation step to ensure removal of material that pellets at 16,000×g (save some supernatant as total input, 50ul*2 tubes).
6. Wash the beads before use it 1ml Pi-IP buffer, 2000×g for 1 min, 2 times
7. Transfer the supernatant to the beads at a ratio of 4ml : 15μl beads, rotate at 4℃ for 1 to 1.5 hours.
8. Aliquot the mixture into 1.5 ml EP tubes, spin at about 2000×g for 1 min, remove the supernatant completely by fine tips.
9. Wash the beads 3-5 times with 1 ml of Pi-IP buffer, spin at about 2000×g for 1 min between washing.
10. Remove as much of the supernatant as possible, add 2 folds bead volume 2×SDS sample buffer, vortex well and incubate on ice for 10 min.
11. Boil the samples for 5-10 min (~100℃), vortex again and spin at about 1000×g for 2 min.
12. Transfer the supernatant to a new tube. If the supernatant still contains beads, spin at 13000×g for 2 min at 4℃ and transfer the supernatant to a new tube.
