# Supplementary material for: Glutathione S-transferase activity facilitates rice tolerance to the barnyard grass root exudate DIMBOA
Source: BMC Plant Biol. 2024 Feb 17;24:117. doi: 10.1186/s12870-024-04802-5 (PMC10874003; doi:10.1186/s12870-024-04802-5)
Supplement: Supplementary file 2 — Supplementary Material 2: Fig. S2. Amounts of differentially expressed proteins from the roots of PI312777 and Lemont after treatment with DIMBOA [file 12870_2024_4802_MOESM2_ESM.pdf]

Accession	Description	Coverage	# Peptides	# PSMs	Area: F3: Sampl	emPAI	# Peptides Sequest HT
A0A0E0FR1	Zeta-carotene desaturase OS=Oryza nivara OX=4536 PE=3 SV=1	4.774897681	3	3	6700000	0.17	3
A0A0E0H1H	Zeaxanthin epoxidase, chloroplastic OS=Oryza nivara OX=4536 PE=4 SV=1	3.186646434	2	2	2200000	0.122	2
A0A0E0ITM	Vesicle-fusing ATPase OS=Oryza nivara OX=4536 PE=3 SV=1	1.576872536	1	1		0.055	1
A0A0E0HIU	Very-long-chain 3-oxoacyl-CoA synthase OS=Oryza nivara OX=4536 PE=3 SV=1	1.312335958	1	1	15000000	0.062	1
A0A0E0G25	V-ATPase 69 kDa subunit OS=Oryza nivara OX=4536 PE=3 SV=1	7.085346216	3	3	6100000	0.162	3
A3QQQ3	UTP--glucose-1-phosphate uridylyltransferase OS=Oryza sativa subsp. indica OX=39946 GN=UGP PE=2 SV=1	2.771855011	1	1	4000000	0.075	1
Q84Q70	Uncharacterized protein OSJNBa0071M09.9 OS=Oryza sativa subsp. japonica OX=39947 GN=OSJNBa0071M09.9 PE=3 SV=1	4.180064309	1	1	1800000	0.122	1
A0A7W7XW	Uncharacterized protein OS=Streptomyces scabiei OX=1930 GN=FHR26_006661 PE=4 SV=1	3.529411765	1	2	1100000	0.101	1
B9G2M9	Uncharacterized protein OS=Oryza sativa subsp. japonica OX=39947 GN=OsJ_28725 PE=4 SV=1	5.882352941	1	1	14000000	0.179	1
B9G1W3	Uncharacterized protein OS=Oryza sativa subsp. japonica OX=39947 GN=OsJ_28011 PE=4 SV=1	3.592814371	2	2	11000000	0.389	2
A3BQX7	Uncharacterized protein OS=Oryza sativa subsp. japonica OX=39947 GN=OsJ_26511 PE=3 SV=1	9.090909091	2	2	4700000	0.259	2
B9FVD6	Uncharacterized protein OS=Oryza sativa subsp. japonica OX=39947 GN=OsJ_23002 PE=3 SV=1	2.118003026	1	1	2400000	0.062	1
A3BFV7	Uncharacterized protein OS=Oryza sativa subsp. japonica OX=39947 GN=OsJ_22825 PE=3 SV=1	6.310679612	1	1	13000000	0.166	1
B9FS66	Uncharacterized protein OS=Oryza sativa subsp. japonica OX=39947 GN=OsJ_20585 PE=4 SV=1	1.140250855	1	1	860000	0.044	1
B9FMC0	Uncharacterized protein OS=Oryza sativa subsp. japonica OX=39947 GN=OsJ_17039 PE=3 SV=1	5.588822355	2	2	4800000	0.202	2
B9FF36	Uncharacterized protein OS=Oryza sativa subsp. japonica OX=39947 GN=OsJ_14740 PE=3 SV=1	18.61702128	3	3	13000000	0.778	3
A3ATE4	Uncharacterized protein OS=Oryza sativa subsp. japonica OX=39947 GN=OsJ_14634 PE=3 SV=1	9.777347531	8	8	9700000	0.286	8
A3AMD8	Uncharacterized protein OS=Oryza sativa subsp. japonica OX=39947 GN=OsJ_12459 PE=3 SV=1	4.340567613	2	2	3000000	0.136	2
B9F4C0	Uncharacterized protein OS=Oryza sativa subsp. japonica OX=39947 GN=OsJ_05928 PE=4 SV=1	8.431372549	3	3	5500000	0.334	3
A2ZQ33	Uncharacterized protein OS=Oryza sativa subsp. japonica OX=39947 GN=OsJ_00667 PE=3 SV=1	5.966587112	2	2	13000000	0.179	2
A2Z9K8	Uncharacterized protein OS=Oryza sativa subsp. indica OX=39946 GN=OsI_34418 PE=3 SV=1	14.46808511	4	13	4600000	1.738	4
A2Z9K3	Uncharacterized protein OS=Oryza sativa subsp. indica OX=39946 GN=OsI_34413 PE=4 SV=1	35.93073593	8	33	9600000000	2.875	8
A2Z9J2	Uncharacterized protein OS=Oryza sativa subsp. indica OX=39946 GN=OsI_34391 PE=3 SV=1	13.99176955	3	3	12000000	0.501	3
A2Z9J0	Uncharacterized protein OS=Oryza sativa subsp. indica OX=39946 GN=OsI_34389 PE=3 SV=1	24.18032787	6	6	95000000	1.371	6
B8BA11	Uncharacterized protein OS=Oryza sativa subsp. indica OX=39946 GN=OsI_28948 PE=4 SV=1	33.56643357	9	11	43000000	3.437	9
A2YML2	Uncharacterized protein OS=Oryza sativa subsp. indica OX=39946 GN=OsI_26464 PE=3 SV=1	4.87804878	1	1	4400000	0.155	1
A2YLT1	Uncharacterized protein OS=Oryza sativa subsp. indica OX=39946 GN=OsI_26177 PE=3 SV=1	15.36312849	4	4	31000000	0.551	4
B8B879	Uncharacterized protein OS=Oryza sativa subsp. indica OX=39946 GN=OsI_25294 PE=4 SV=1	6.103286385	3	3	2900000	0.218	3
B8B3U4	Uncharacterized protein OS=Oryza sativa subsp. indica OX=39946 GN=OsI_23436 PE=4 SV=1	8.951965066	3	4	4100000	0.269	3
B8B3P0	Uncharacterized protein OS=Oryza sativa subsp. indica OX=39946 GN=OsI_22016 PE=3 SV=1	4.332129964	1	1	5500000	0.122	1
A2Y534	Uncharacterized protein OS=Oryza sativa subsp. indica OX=39946 GN=OsI_20107 PE=3 SV=1	11.17021277	3	3	1400000	0.334	3
A2XYG6	Uncharacterized protein OS=Oryza sativa subsp. indica OX=39946 GN=OsI_17742 PE=3 SV=1	7.125307125	3	3	3800000	0.369	3
B8ASM5	Uncharacterized protein OS=Oryza sativa subsp. indica OX=39946 GN=OsI_16848 PE=4 SV=1	4.461538462	4	4	15000000	0.112	4
A2XPB4	Uncharacterized protein OS=Oryza sativa subsp. indica OX=39946 GN=OsI_14427 PE=3 SV=1	2.054794521	1	1	4800000	0.064	1
B8AMT7	Uncharacterized protein OS=Oryza sativa subsp. indica OX=39946 GN=OsI_14218 PE=3 SV=1	18.03713528	5	5	8500000	0.616	5
A2XNL2	Uncharacterized protein OS=Oryza sativa subsp. indica OX=39946 GN=OsI_14156 PE=3 SV=1	8.823529412	1	1	7000000	0.212	1
A2XN99	Uncharacterized protein OS=Oryza sativa subsp. indica OX=39946 GN=OsI_14032 PE=4 SV=1	9.88700565	3	4	5700000	0.52	3
A2XMP7	Uncharacterized protein OS=Oryza sativa subsp. indica OX=39946 GN=OsI_13813 PE=3 SV=1	14.86810552	4	4	7700000	0.492	4
A2XM46	Uncharacterized protein OS=Oryza sativa subsp. indica OX=39946 GN=OsI_13588 PE=3 SV=1	11.65048544	2	2	52000000	0.359	2
B8AKV8	Uncharacterized protein OS=Oryza sativa subsp. indica OX=39946 GN=OsI_12293 PE=3 SV=1	34.9009901	13	15	17000000	2.3	13
A2XAQ5	Uncharacterized protein OS=Oryza sativa subsp. indica OX=39946 GN=OsI_09337 PE=3 SV=1	3.578528827	1	1	3500000	0.08	1
B8AGU8	Uncharacterized protein OS=Oryza sativa subsp. indica OX=39946 GN=OsI_05455 PE=3 SV=1	5.609756098	2	2	5200000	0.172	2
B8A962	Uncharacterized protein OS=Oryza sativa subsp. indica OX=39946 GN=OsI_05201 PE=4 SV=1	40.69264069	10	29	290000000	9	10
B8A7L7	Uncharacterized protein OS=Oryza sativa subsp. indica OX=39946 GN=OsI_03288 PE=4 SV=1	5.023923445	2	2	4600000	0.202	2
B8A8L8	Uncharacterized protein OS=Oryza sativa subsp. indica OX=39946 GN=OsI_02088 PE=3 SV=1	18.31831832	4	4	13000000	0.52	4
A2WK17	Uncharacterized protein OS=Oryza sativa subsp. indica OX=39946 GN=OsI_00166 PE=4 SV=1	13.86138614	3	3	17000000	0.54	3

A0A0E0HIC8	Uncharacterized protein OS=Oryza nivara OX=4536 PE=4 SV=1	53.1120332	17	82	5500000000	114.478	17
A0A0E0HIB8	Uncharacterized protein OS=Oryza nivara OX=4536 PE=4 SV=1	52.26337449	15	44	960000000	30.623	15
A0A0E0IUD7	Uncharacterized protein OS=Oryza nivara OX=4536 PE=4 SV=1	32.35294118	11	13	88000000	2.03	11
A0A0E0IVE3	Uncharacterized protein OS=Oryza nivara OX=4536 PE=4 SV=1	10.76023392	9	25	89000000	0.638	9
A0A0E0HEF	Uncharacterized protein OS=Oryza nivara OX=4536 PE=4 SV=1	31.02189781	7	8	85000000	1.955	7
A0A0E0HDS	Uncharacterized protein OS=Oryza nivara OX=4536 PE=4 SV=1	17.55952381	5	6	13000000	0.823	5
A0A0E0ISN2	Uncharacterized protein OS=Oryza nivara OX=4536 PE=4 SV=1	15.99147122	5	5	11000000	0.585	5
A0A0E0I610	Uncharacterized protein OS=Oryza nivara OX=4536 PE=4 SV=1	6.338028169	5	5	11000000	0.238	5
A0A0E0H8C	Uncharacterized protein OS=Oryza nivara OX=4536 PE=4 SV=1	25	5	5	6300000	0.848	5
A0A0E0IVK6	Uncharacterized protein OS=Oryza nivara OX=4536 PE=4 SV=1	17.69547325	4	16	190000000	1.929	4
A0A0E0FRK1	Uncharacterized protein OS=Oryza nivara OX=4536 PE=4 SV=1	27.19665272	4	4	30000000	0.668	4
A0A0E0IVD2	Uncharacterized protein OS=Oryza nivara OX=4536 PE=4 SV=1	8.745247148	4	4	15000000	0.334	4
A0A0E0HGU	Uncharacterized protein OS=Oryza nivara OX=4536 PE=4 SV=1	10.80508475	4	4	6700000	0.374	4
A0A0E0HFL6	Uncharacterized protein OS=Oryza nivara OX=4536 PE=4 SV=1	22.56637168	4	5	19000000	0.668	4
A0A0E0G8Z	Uncharacterized protein OS=Oryza nivara OX=4536 PE=4 SV=1	13.95348837	3	3	11000000	0.389	3
A0A0E0GKP	Uncharacterized protein OS=Oryza nivara OX=4536 PE=4 SV=1	11.07692308	3	3	8200000	0.413	3
A0A0E0J7K7	Uncharacterized protein OS=Oryza nivara OX=4536 PE=4 SV=1	11.87335092	3	3	7400000	0.369	3
A0A0E0GW	Uncharacterized protein OS=Oryza nivara OX=4536 PE=4 SV=1	12.94642857	2	2	4600000	0.359	2
A0A0E0FN04	Uncharacterized protein OS=Oryza nivara OX=4536 PE=4 SV=1	6.097560976	2	2	6400000	0.136	2
A0A0E0IYB0	Uncharacterized protein OS=Oryza nivara OX=4536 PE=4 SV=1	4.280155642	2	3	7700000	0.141	2
A0A0E0H354	Uncharacterized protein OS=Oryza nivara OX=4536 PE=4 SV=1	1.908065915	2	2	2000000	0.066	2
A0A0E0HBX	Uncharacterized protein OS=Oryza nivara OX=4536 PE=4 SV=1	11.52073733	2	2	2800000	0.334	2
A0A0E0FRJ9	Uncharacterized protein OS=Oryza nivara OX=4536 PE=4 SV=1	7.746478873	2	2	2900000	0.334	2
A0A0E0FY40	Uncharacterized protein OS=Oryza nivara OX=4536 PE=4 SV=1	6.233062331	2	2	23000000	0.334	2
A0A0E0FFP0	Uncharacterized protein OS=Oryza nivara OX=4536 PE=4 SV=1	5.394190871	2	2	4600000	0.172	2
A0A0E0J795	Uncharacterized protein OS=Oryza nivara OX=4536 PE=4 SV=1	5.799648506	2	2	3400000	0.179	2
A0A0E0FJF4	Uncharacterized protein OS=Oryza nivara OX=4536 PE=4 SV=1	5.849582173	2	2	6200000	0.259	2
A0A0E0IGE4	Uncharacterized protein OS=Oryza nivara OX=4536 PE=4 SV=1	4.898648649	2	2	1300000	0.141	2
A0A0E0GKX	Uncharacterized protein OS=Oryza nivara OX=4536 PE=4 SV=1	5.098039216	1	1	3000000	0.155	1
A0A0E0H2H	Uncharacterized protein OS=Oryza nivara OX=4536 PE=4 SV=1	3.225806452	1	1	2200000	0.129	1
A0A0E0I922	Uncharacterized protein OS=Oryza nivara OX=4536 PE=4 SV=1	3.793103448	1	1	20000000	0.145	1
A0A0E0G5N	Uncharacterized protein OS=Oryza nivara OX=4536 PE=4 SV=1	3.384615385	1	1	5200000	0.116	1
A0A0E0J971	Uncharacterized protein OS=Oryza nivara OX=4536 PE=4 SV=1	2.087682672	1	1	3000000	0.096	1
A0A0E0IC83	Uncharacterized protein OS=Oryza nivara OX=4536 PE=4 SV=1	2.624671916	1	1	2300000	0.105	1
A0A0E0J9G5	Uncharacterized protein OS=Oryza nivara OX=4536 PE=4 SV=1	2.144249513	1	1	1100000	0.089	1
A0A0E0J9R1	Uncharacterized protein OS=Oryza nivara OX=4536 PE=4 SV=1	3.418803419	1	1	2100000	0.116	1
A0A0E0FLW	Uncharacterized protein OS=Oryza nivara OX=4536 PE=4 SV=1	0.942587832	1	1	2600000	0.03	1
A0A0E0FV30	Uncharacterized protein OS=Oryza nivara OX=4536 PE=4 SV=1	2.444987775	1	1	770000	0.089	1
A0A0E0HUY	Uncharacterized protein OS=Oryza nivara OX=4536 PE=3 SV=1	33.55408389	12	16	32000000	2.03	12
A0A0E0HK0	Uncharacterized protein OS=Oryza nivara OX=4536 PE=3 SV=1	20.63227953	9	10	21000000	0.833	9
A0A0E0IYI4	Uncharacterized protein OS=Oryza nivara OX=4536 PE=3 SV=1	31.20204604	7	10	54000000	1.291	7
A0A0E0GCD	Uncharacterized protein OS=Oryza nivara OX=4536 PE=3 SV=1	21.4673913	7	7	20000000	0.957	7
A0A0E0FL87	Uncharacterized protein OS=Oryza nivara OX=4536 PE=3 SV=1	25.96153846	4	4	92000000	1.154	4
A0A0E0FW4	Uncharacterized protein OS=Oryza nivara OX=4536 PE=3 SV=1	14.62765957	4	4	12000000	0.468	4
A0A0E0H5N	Uncharacterized protein OS=Oryza nivara OX=4536 PE=3 SV=1	15.49815498	4	4	10000000	0.624	4
A0A0E0FZU	Uncharacterized protein OS=Oryza nivara OX=4536 PE=3 SV=1	6.013363029	3	5	9100000	0.252	3
A0A0E0HZY4	Uncharacterized protein OS=Oryza nivara OX=4536 PE=3 SV=1	12.06896552	3	3	7100000	0.874	3

A0A0E0FVL	Uncharacterized protein OS=Oryza nivara OX=4536 PE=3 SV=1	6.635802469	3	3	5300000	0.205	3
A0A0E0HIH	Uncharacterized protein OS=Oryza nivara OX=4536 PE=3 SV=1	10.74380165	3	3	7500000	0.438	3
A0A0E0IWV	Uncharacterized protein OS=Oryza nivara OX=4536 PE=3 SV=1	3.859060403	2	2	7000000	0.129	2
A0A0E0IMV	Uncharacterized protein OS=Oryza nivara OX=4536 PE=3 SV=1	9.539473684	2	2	4700000	0.292	2
A0A0E0FW	Uncharacterized protein OS=Oryza nivara OX=4536 PE=3 SV=1	1.639344262	2	2	4900000	0.083	2
A0A0E0IQI1	Uncharacterized protein OS=Oryza nivara OX=4536 PE=3 SV=1	6.702412869	2	2	7400000	0.233	2
A0A0E0J9Q	Uncharacterized protein OS=Oryza nivara OX=4536 PE=3 SV=1	4.684684685	2	2	2800000	0.16	2
A0A0E0GY3	Uncharacterized protein OS=Oryza nivara OX=4536 PE=3 SV=1	5.102040816	2	2	9100000	0.202	2
A0A0E0GL8	Uncharacterized protein OS=Oryza nivara OX=4536 PE=3 SV=1	12.08459215	2	2	3700000	0.259	2
A0A0E0H6P	Uncharacterized protein OS=Oryza nivara OX=4536 PE=3 SV=1	2.765957447	1	1	6800000	0.116	1
A0A0E0J80	Uncharacterized protein OS=Oryza nivara OX=4536 PE=3 SV=1	8.791208791	1	1	17000000	0.145	1
A0A0E0GTS	Uncharacterized protein OS=Oryza nivara OX=4536 PE=3 SV=1	2.07253886	1	1	1000000	0.059	1
A0A0E0FXK	Uncharacterized protein OS=Oryza nivara OX=4536 PE=3 SV=1	3.319502075	1	1	3800000	0.086	1
A0A0E0HJP	Uncharacterized protein OS=Oryza nivara OX=4536 PE=3 SV=1	2.886597938	1	1	1300000	0.155	1
A0A0E0GQ3	Uncharacterized protein OS=Oryza nivara OX=4536 PE=3 SV=1	4.407713499	1	1	6300000	0.136	1
A0A0E0I640	Uncharacterized protein OS=Oryza nivara OX=4536 PE=3 SV=1	2.189781022	1	1	2700000	0.064	1
A0A0E0GW	Uncharacterized protein OS=Oryza nivara OX=4536 PE=3 SV=1	4.109589041	1	1	7200000	0.334	1
A0A0E0H9D	Uncharacterized protein OS=Oryza nivara OX=4536 PE=3 SV=1	2.242990654	1	1	2600000	0.072	1
A0A0E0GRC	Uncharacterized protein OS=Oryza nivara OX=4536 PE=3 SV=1	1.679389313	1	1	510000	0.059	1
A0A0E0H47	Uncharacterized protein OS=Oryza nivara OX=4536 PE=3 SV=1	3.472222222	1	1	9400000	0.233	1
A0A0E0GA	Uncharacterized protein OS=Oryza nivara OX=4536 PE=3 SV=1	2.96735905	1	1	1200000	0.194	1
A0A0E0HL1	Uncharacterized protein OS=Oryza nivara OX=4536 PE=3 SV=1	2.772277228	1	1	3400000	0.089	1
Q8H8C2	Uncharacterized protein OJ1134F05.7 OS=Oryza sativa subsp. japonica OX=39947 GN=OJ1134F05.7 PE=4 SV=1	4.260651629	1	1	12000000	0.129	1
Q84QA8	Uncharacterized protein OJ1012B02.13 OS=Oryza sativa subsp. japonica OX=39947 GN=OJ1012B02.13 PE=3 SV=1	2.582159624	1	1	1900000	0.096	1
B8A8E2	UDP-glucose-fructose-phosphate glucosyltransferase OS=Oryza sativa subsp. indica OX=39946 GN=OsI_04951 PE=3 SV=1	1.209677419	1	1	1600000	0.031	1
A0A0B4U1	UDP-glucose 6-dehydrogenase (Fragment) OS=Oryza sativa OX=4530 GN=LOC_Os03g55070.1 PE=2 SV=1	12.91666667	5	5	15000000	0.377	5
B8B8B1	UDP-arabinopyranose mutase OS=Oryza sativa subsp. indica OX=39946 GN=OsI_26792 PE=3 SV=1	8.469945355	3	3	8400000	0.318	3
A0A0P0X6U	Ubiquitin OS=Oryza sativa subsp. japonica OX=39947 GN=Os07g0489500 PE=3 SV=1	14.96062992	3	5	34000000	0.585	3
A0A0E0J4R	TYR_PHOSPHATASE_2 domain-containing protein OS=Oryza nivara OX=4536 PE=4 SV=1	9.230769231	3	3	4900000	0.35	3
P46265	Tubulin beta-5 chain OS=Oryza sativa subsp. japonica OX=39947 GN=TUBB5 PE=1 SV=1	25.05592841	9	11	6100000	1.61	9
A0A0E0ITA5	Tubulin beta chain OS=Oryza nivara OX=4536 PE=3 SV=1	25.16853933	9	11	9100000	1.61	9
Q0D584	Tubulin alpha chain OS=Oryza sativa subsp. japonica OX=39947 GN=Os07g0574800 PE=3 SV=1	5.555555556	2	2	25000000	0.233	2
B8B8G2	Tubulin alpha chain OS=Oryza sativa subsp. indica OX=39946 GN=OsI_25395 PE=3 SV=1	5.567928731	2	2	6000000	0.212	2
A0A0E0J095	Tubulin alpha chain OS=Oryza nivara OX=4536 PE=3 SV=1	5.321507761	2	2	49000000	0.212	2
A0A0E0GX7	Tr-type G domain-containing protein OS=Oryza nivara OX=4536 PE=3 SV=1	6.40569395	4	4	9000000	0.198	4
A0A218KL5	Triosephosphate isomerase OS=Oryza sativa OX=4530 PE=2 SV=1	5.533596838	1	1	5600000	0.179	1
Q2RAH8	Transposon protein, putative, unclassified OS=Oryza sativa subsp. japonica OX=39947 GN=LOC_Os11g05340 PE=4 SV=1	2.278481013	1	1	2200000	0.032	1
A0A0E0HKI	Transketolase OS=Oryza nivara OX=4536 PE=3 SV=1	6.191117093	4	5	6500000	0.233	4
A2XPF9	Thioredoxin-like_fold domain-containing protein OS=Oryza sativa subsp. indica OX=39946 GN=OsI_14471 PE=4 SV=1	5.309734513	1	1	6100000	0.166	1
Q84NN4	Thioredoxin-like protein CDSP32, chloroplastic OS=Oryza sativa subsp. japonica OX=39947 GN=CDSP32 PE=2 SV=1	5.315614618	1	1	1500000	0.129	1
A0A0E0G7U	Thioredoxin-dependent peroxiredoxin OS=Oryza nivara OX=4536 PE=4 SV=1	17.11026616	3	3	29000000	0.438	3
B9GC77	Thioredoxin domain-containing protein OS=Oryza sativa subsp. japonica OX=39947 GN=OsJ_35463 PE=4 SV=1	3.603603604	1	1	3900000	0.083	1
B8AI99	Thioredoxin domain-containing protein OS=Oryza sativa subsp. indica OX=39946 GN=OsI_07299 PE=4 SV=1	3.173164098	2	2	2600000	0.084	2
A2YM28	Thiamine thiazole synthase, chloroplastic OS=Oryza sativa subsp. indica OX=39946 GN=THI1 PE=3 SV=1	37.11048159	8	11	89000000	1.848	8
B8BLP4	TCTP domain-containing protein OS=Oryza sativa subsp. indica OX=39946 GN=OsI_36917 PE=3 SV=1	1.807228916	1	1	1400000	0.077	1
A2YNN3	Str_synth domain-containing protein OS=Oryza sativa subsp. indica OX=39946 GN=OsI_26852 PE=3 SV=1	9.308510638	2	2	4600000	0.274	2
A0A0E0FZ2	SRP54 domain-containing protein OS=Oryza nivara OX=4536 PE=4 SV=1	5.80474934	2	2	2200000	0.259	2

A0A0E0GJ07	Smr domain-containing protein OS=Oryza nivara OX=4536 PE=3 SV=1	1.923076923	1	1	2500000	0.058	1
B8AI25	SET domain-containing protein OS=Oryza sativa subsp. indica OX=39946 GN=OsI_08832 PE=4 SV=1	2.674897119	1	1	3100000	0.096	1
Q7Y0B9	Serine/threonine-protein kinase SAPK8 OS=Oryza sativa subsp. japonica OX=39947 GN=SAPK8 PE=1 SV=1	3.504043127	1	1	400000	0.116	1
Q0D798	Serine/threonine protein phosphatase 2A regulatory subunit OS=Oryza sativa subsp. japonica OX=39947 GN=Os07g0274800 PE=3	1.160541586	1	1	500000	0.072	1
Q7Y1F0	Serine hydroxymethyltransferase OS=Oryza sativa subsp. japonica OX=39947 GN=OSJNBa0057G07.17 PE=3 SV=1	8.438061041	3	3	11000000	0.212	3
A0A542HVU	S-DNA-T family DNA segregation ATPase FtsK/SpoIIIE OS=Streptomyces sp. SLBN-115 OX=2768453 GN=FBY34_3941 PE=4 SV=1	0.681302044	1	1	20000000	0.035	1
A0A0E0JCG7	SAM_MPBQ_MSBQ_MT domain-containing protein OS=Oryza nivara OX=4536 PE=3 SV=1	12.89398281	3	4	11000000	0.585	3
B7EKA4	S-adenosylmethionine synthase OS=Oryza sativa subsp. japonica OX=39947 PE=2 SV=1	8.333333333	3	4	8400000	0.492	3
B8BIW2	Sacchrp_dh_NADP domain-containing protein OS=Oryza sativa subsp. indica OX=39946 GN=OsI_35044 PE=4 SV=1	3.044496487	1	1		0.093	1
B9F6V9	S5 DRBM domain-containing protein OS=Oryza sativa subsp. japonica OX=39947 GN=OsJ_13042 PE=3 SV=1	13.73239437	4	4	7100000	0.624	4
A0A0E0HQ1	RuvB-like helicase OS=Oryza nivara OX=4536 PE=3 SV=1	3.354297694	1	1	1900000	0.086	1
B9FAQ8	RNase H domain-containing protein OS=Oryza sativa subsp. japonica OX=39947 GN=OsJ_12207 PE=4 SV=1	4.47761194	2	2	5100000	0.172	2
Q6YZW2	RNA-binding protein 208 OS=Oryza sativa subsp. japonica OX=39947 GN=RBP-208 PE=1 SV=1	2.237136465	1	1	4300000	0.136	1
A2ZCZ7	Rieske domain-containing protein OS=Oryza sativa subsp. indica OX=39946 GN=OsI_35659 PE=4 SV=1	32.49097473	7	7	150000000	1.448	7
B8APA6	Ribulose-phosphate 3-epimerase OS=Oryza sativa subsp. indica OX=39946 GN=OsI_10180 PE=3 SV=1	20.80291971	3	3	30000000	0.638	3
H2KVX3	Ribulose bisphosphate carboxylase/oxygenase activase, chloroplast, putative, expressed OS=Oryza sativa subsp. japonica OX=3994	58.54341737	19	44	2200000000	32.246	19
A2ZJQ0	Ribulose bisphosphate carboxylase small subunit OS=Oryza sativa subsp. indica OX=39946 GN=OsI_38046 PE=3 SV=1	53.14285714	8	11	480000000	2.981	8
Q339G9	Ribulose bisphosphate carboxylase large chain OS=Oryza sativa subsp. japonica OX=39947 GN=LOC_Os10g21280 PE=3 SV=2	53.03867403	20	86	840000000	66.002	20
Q5K3B1	Ribulose bisphosphate carboxylase large chain (Fragment) OS=Oryza sativa OX=4530 GN=rbcL PE=3 SV=1	54.41176471	23	93	640000000	57.78	23
A0A7G8PYR	Ribulose bisphosphate carboxylase large chain (Fragment) OS=Oryza sativa OX=4530 GN=rbcL PE=3 SV=1	51.57232704	6	21	69000000	99	6
A0A0E0FEX6	Ribosomal_S7 domain-containing protein OS=Oryza nivara OX=4536 PE=3 SV=1	6.5	1	1	4300000	0.233	1
B8AU49	Ribosomal_S17_N domain-containing protein OS=Oryza sativa subsp. indica OX=39946 GN=OsI_17370 PE=4 SV=1	2.056074766	1	1	1600000	0.08	1
A0A0E0GJR0	Ribosomal_S10 domain-containing protein OS=Oryza nivara OX=4536 PE=3 SV=1	2.04778157	1	1	6500000	0.07	1
A0A0E0ICN1	Ribosomal_L2_C domain-containing protein OS=Oryza nivara OX=4536 PE=3 SV=1	9.195402299	2	2	1100000	0.292	2
B8AYH0	Ribosomal_L16 domain-containing protein OS=Oryza sativa subsp. indica OX=39946 GN=OsI_18615 PE=3 SV=1	10.26785714	2	2	6900000	0.334	2
A0A0E0HE9	Ribosomal_L16 domain-containing protein OS=Oryza nivara OX=4536 PE=3 SV=1	10.71428571	2	2	5000000	0.334	2
A0A0E0HXB	Ribose-5-phosphate isomerase OS=Oryza nivara OX=4536 PE=3 SV=1	7.594936709	3	3	9000000	0.25	3
A2YZA3	Rhodanese domain-containing protein OS=Oryza sativa subsp. indica OX=39946 GN=OsI_30678 PE=4 SV=1	4.184100418	1	1	2500000	0.129	1
B8AI07	Rhodanese domain-containing protein OS=Oryza sativa subsp. indica OX=39946 GN=OsI_08794 PE=4 SV=1	5.764075067	3	3	7700000	0.166	3
A0A0E0J535	RF_PROK_I domain-containing protein OS=Oryza nivara OX=4536 PE=3 SV=1	2.884615385	1	1	4200000	0.093	1
A0A0E0FST5	Reticulon-like protein OS=Oryza nivara OX=4536 PE=4 SV=1	4.347826087	1	1	4500000	0.212	1
A0A0E0H7T	RCK N-terminal domain-containing protein OS=Oryza nivara OX=4536 PE=4 SV=1	1.053864169	2	2	5300000	0.033	2
B9F0A5	Quinol--cytochrome-c reductase OS=Oryza sativa subsp. japonica OX=39947 GN=OsJ_06940 PE=3 SV=1	8.894230769	2	2	11000000	0.259	2
Q6AVA8	Pyruvate, phosphate dikinase 1, chloroplastic OS=Oryza sativa subsp. japonica OX=39947 GN=PPDK1 PE=1 SV=1	2.639915523	2	2	7000000	0.077	2
Q259K4	Pyruvate kinase OS=Oryza sativa OX=4530 GN=H0402C08.9 PE=3 SV=1	4.69667319	2	2	4300000	0.15	2
A0A0E0HXE	Pyruvate kinase OS=Oryza nivara OX=4536 PE=3 SV=1	11.64383562	8	9	43000000	0.339	8
B8ABM9	Pyr_redox_2 domain-containing protein OS=Oryza sativa subsp. indica OX=39946 GN=OsI_04297 PE=4 SV=1	4.426559356	2	2	2300000	0.155	2
Q84SS7	Putative ribosomal protein S5 OS=Oryza sativa subsp. japonica OX=39947 GN=OSJNBb0047D08.4 PE=3 SV=1	2.752293578	1	1	10000000	0.129	1
Q9SNL7	Putative magnesium-protoporphyrin IX methyltransferase OS=Oryza sativa subsp. japonica OX=39947 GN=134P10.4 PE=4 SV=1	6.748466258	2	2	18000000	0.233	2
Q94I53	Putative hydrolase OS=Oryza sativa subsp. japonica OX=39947 GN=OSJNBa0084C09.19 PE=4 SV=1	5.483028721	2	2	8100000	0.222	2
A0A0P0X2Y5	Putative glutathione S-transferase GST27 OS=Oryza sativa subsp. japonica OX=39947 GN=OSJNBa0050F10.6 PE=4 SV=1	30.63829787	6	6	16000000	1.154	6
A0A0P0X6V	Putative ATP-dependent proteinase BsgA OS=Oryza sativa subsp. japonica OX=39947 GN=P0409B11.11 PE=4 SV=1	4.810996564	1	1	4600000	0.194	1
Q6Z2T4	Putative ATPase OS=Oryza sativa subsp. japonica OX=39947 GN=OJ1118_G04.14 PE=4 SV=1	4.926108374	1	1	16000000	0.096	1
A0A0E0I902	P-type H(+)-exporting transporter OS=Oryza nivara OX=4536 PE=3 SV=1	1.460564752	1	1	7300000	0.042	1
A0A0E0JBR9	PSI-K OS=Oryza nivara OX=4536 PE=3 SV=1	6.766917293	1	1	44000000	0.334	1
A0A0E0IT94	PSI-F OS=Oryza nivara OX=4536 PE=3 SV=1	12.71186441	2	2	7900000	0.425	2
Q8W3D9	Protochlorophyllide reductase B, chloroplastic OS=Oryza sativa subsp. japonica OX=39947 GN=PORB PE=2 SV=1	25.87064677	8	13	49000000	1.783	8

B8AA22	Protein-serine/threonine phosphatase OS=Oryza sativa subsp. indica OX=39946 GN=OsI_02395 PE=3 SV=1	9.487179487	3	3	4900000	0.389	3
B9G712	Protein-methionine-S-oxide reductase OS=Oryza sativa subsp. japonica OX=39947 GN=OsJ_32480 PE=3 SV=1	10	3	4	9100000	0.304	3
Q10A77	Protein TIC 62, chloroplastic OS=Oryza sativa subsp. japonica OX=39947 GN=TIC62 PE=1 SV=1	3.420523139	1	1	12000000	0.122	1
Q6ETQ7	Protein THYLAKOID RHODANESE-LIKE, chloroplastic OS=Oryza sativa subsp. japonica OX=39947 GN=TROL PE=1 SV=1	4.184100418	1	2	1600000	0.389	1
A0A0E0G9G	Protein kinase domain-containing protein OS=Oryza nivara OX=4536 PE=4 SV=1	6.937394247	3	3	11000000	0.25	3
A0A0E0G8G	Protein kinase domain-containing protein OS=Oryza nivara OX=4536 PE=4 SV=1	4.444444444	2	2	2100000	0.179	2
B8BCJ0	Protein disulfide-isomerase OS=Oryza sativa subsp. indica OX=39946 GN=OsI_31602 PE=3 SV=1	2.857142857	1	1	5600000	0.089	1
Q10QW8	Proteasome subunit alpha type OS=Oryza sativa subsp. japonica OX=39947 GN=LOC_Os03g08280 PE=3 SV=1	4.895104895	1	1		0.116	1
B9FVN3	Prolyl-tRNA synthetase OS=Oryza sativa subsp. japonica OX=39947 GN=OsJ_23204 PE=3 SV=1	7.52293578	3	3	9900000	0.225	3
Q6ZJJ1	Probable L-ascorbate peroxidase 4, peroxisomal OS=Oryza sativa subsp. japonica OX=39947 GN=APX4 PE=2 SV=1	4.810996564	1	1	2800000	0.11	1
A2XMN2	Probable glutathione S-transferase GSTU1 OS=Oryza sativa subsp. indica OX=39946 GN=GSTU1 PE=1 SV=1	22.07792208	4	13	82000000	0.833	4
Q651D5	Probable aquaporin PIP2-7 OS=Oryza sativa subsp. japonica OX=39947 GN=PIP2-7 PE=2 SV=2	12.06896552	2	2	6500000	0.52	2
Q69WE1	Plastid-lipid associated protein PAP/fibrillin family-like OS=Oryza sativa subsp. japonica OX=39947 GN=OJ1103_E04.105 PE=4 SV=	7.918552036	3	3	6100000	0.25	3
A0A0E0IM0	PKS_ER domain-containing protein OS=Oryza nivara OX=4536 PE=4 SV=1	9.550561798	2	2	4000000	0.233	2
A0A0E0J853	PKS_ER domain-containing protein OS=Oryza nivara OX=4536 PE=4 SV=1	6.651884701	2	2	7300000	0.233	2
A0A0E0GFI5	PKS_ER domain-containing protein OS=Oryza nivara OX=4536 PE=4 SV=1	4	1	1	4500000	0.105	1
J7EYL8	Photosystem II protein D1 OS=Oryza sativa subsp. indica OX=39946 GN=psbA PE=3 SV=1	17.28045326	4	4	17000000	0.778	4
Q7M1Y7	Photosystem II oxygen-evolving complex protein 2 (Fragment) OS=Oryza sativa OX=4530 PE=1 SV=1	35.13513514	1	1	25000000	2.162	1
E9KIM8	Photosystem II D2 protein OS=Oryza sativa subsp. japonica OX=39947 GN=psbD PE=3 SV=1	12.53481894	3	3	38000000	0.874	3
D0EKL9	Photosystem II CP47 reaction center protein OS=Oryza sativa OX=4530 GN=psbB PE=3 SV=1	2.755905512	1	1		0.122	1
A0A1W5HL3	Photosystem II CP43 reaction center protein OS=Oryza nivara OX=4536 GN=psbC PE=3 SV=1	11.41649049	4	4	23000000	0.778	4
A2WXD9	Photosystem II 22 kDa protein 1, chloroplastic OS=Oryza sativa subsp. indica OX=39946 GN=PSBS1 PE=1 SV=1	14.17910448	3	3	19000000	0.54	3
B9FZJ0	Photosystem II 10 kDa polypeptide, chloroplastic OS=Oryza sativa subsp. japonica OX=39947 GN=OsJ_26372 PE=3 SV=1	13.42281879	1	1	3200000	0.468	1
E9KIP0	Photosystem I P700 chlorophyll a apoprotein A2 OS=Oryza sativa subsp. japonica OX=39947 GN=psaB PE=3 SV=1	1.62601626	1	1	4100000	0.116	1
Q7M1U9	Photosystem I 9K protein (Fragment) OS=Oryza sativa OX=4530 PE=1 SV=1	18	1	1	12000000	1.154	1
Q8GRU9	Phosphoribulokinase OS=Oryza sativa subsp. indica OX=39946 GN=OsI_08574 PE=2 SV=1	22.3325062	7	8	13000000	1.081	7
Q09HR2	Phosphoglycerate kinase OS=Oryza sativa subsp. indica OX=39946 PE=2 SV=1	16.66666667	5	6	28000000	0.61	5
A0A0E0HGC	Phosphoglycerate kinase OS=Oryza nivara OX=4536 PE=3 SV=1	49.07597536	19	38	370000000	8.284	19
A2WLX8	Phosphoenolpyruvate carboxylase OS=Oryza sativa subsp. indica OX=39946 GN=OsI_00846 PE=3 SV=1	1.028999065	1	1	1300000	0.03	1
A0A0P0XH7	Phospho-2-dehydro-3-deoxyheptonate aldolase OS=Oryza sativa subsp. japonica OX=39947 GN=Os08g0484500 PE=3 SV=1	4.95049505	2	2	5300000	0.141	2
Q9S7C5	Phosphatase 2A regulatory A subunit OS=Oryza sativa OX=4530 GN=RPA PE=2 SV=1	1.533219761	1	1		0.068	1
A0A0E0IEF7	Phenylalanyl-tRNA synthetase OS=Oryza nivara OX=4536 PE=3 SV=1	8.035714286	3	3	2800000	0.25	3
Q10SM7	Peroxisomal membrane protein 11-1 OS=Oryza sativa subsp. japonica OX=39947 GN=PEX11-1 PE=2 SV=1	4.641350211	1	1	8300000	0.194	1
A0A0E0H91	Peroxidase OS=Oryza nivara OX=4536 PE=3 SV=1	1.231060606	1	1	4800000	0.035	1
Q0DHL6	Peptidylprolyl isomerase OS=Oryza sativa subsp. japonica OX=39947 GN=Os05g0458100 PE=4 SV=1	5.913978495	1	1	9600000	0.389	1
Q0E4F7	Peptidyl-prolyl cis-trans isomerase OS=Oryza sativa subsp. japonica OX=39947 GN=Os02g0121300 PE=3 SV=1	8.139534884	1	1	1500000	0.194	1
A0A0E0H2J	PDZ domain-containing protein OS=Oryza nivara OX=4536 PE=3 SV=1	10.33653846	3	3	3600000	0.438	3
A2Z3M0	PCI domain-containing protein OS=Oryza sativa subsp. indica OX=39946 GN=OsI_32229 PE=3 SV=1	9.240246407	3	3	8100000	0.225	3
A0A0E0H2B	PCI domain-containing protein OS=Oryza nivara OX=4536 PE=4 SV=1	14.91002571	5	5	3900000	0.585	5
A0A0E0GIK	PCI domain-containing protein OS=Oryza nivara OX=4536 PE=4 SV=1	14.17525773	5	5	2800000	0.585	5
A0A0E0IFI4	PCI domain-containing protein OS=Oryza nivara OX=4536 PE=3 SV=1	10.90534979	4	4	5800000	0.225	4
A0A0E0GWI	PCI domain-containing protein OS=Oryza nivara OX=4536 PE=3 SV=1	6.342494715	3	3	5500000	0.233	3
A0A0E0H2L	PCI domain-containing protein OS=Oryza nivara OX=4536 PE=3 SV=1	5.529953917	2	2	8700000	0.179	2
A0A0E0GIZ5	PCI domain-containing protein OS=Oryza nivara OX=4536 PE=3 SV=1	2.301790281	1	1		0.105	1
A2ZFY8	PAP_fibrillin domain-containing protein OS=Oryza sativa subsp. indica OX=39946 GN=OsI_36693 PE=4 SV=1	8.518518519	2	2	5500000	0.359	2
Q9FP37	p0035H10.3 protein OS=Oryza sativa subsp. japonica OX=39947 GN=P0035H10.3 PE=4 SV=1	2.330097087	1	1	3900000	0.089	1
P83646	Oxygen-evolving enhancer protein 3, chloroplastic OS=Oryza sativa subsp. indica OX=39946 GN=OsI_025465 PE=1 SV=2	9.677419355	1	1	8000000	0.194	1

A0A0P0WB(	OSJNBb0011N17.9 protein OS=Oryza sativa subsp. japonica OX=39947 GN=Os04g0481300 PE=4 SV=1	4.850746269	1	1	6600000	0.136	1
Q7XXC5	OSJNBa0027O01.13 protein OS=Oryza sativa subsp. japonica OX=39947 GN=OSJNBa0027O01.13 PE=4 SV=2	0.566572238	1	3	16000000	0.039	1
Q2QSR7	Os12g0420200 protein OS=Oryza sativa subsp. japonica OX=39947 GN=LOC_Os12g23180 PE=4 SV=2	11.43617021	3	3	9000000	0.35	3
Q2QXS4	Os12g0145100 protein OS=Oryza sativa subsp. japonica OX=39947 GN=LOC_Os12g05050 PE=2 SV=1	11.34453782	2	2	4800000	0.425	2
A0A0P0Y34	Os11g0546000 protein OS=Oryza sativa subsp. japonica OX=39947 GN=Os11g0546000 PE=4 SV=1	2.649006623	1	1	1700000	0.07	1
A0A0P0Y3G	Os11g0544800 protein (Fragment) OS=Oryza sativa subsp. japonica OX=39947 GN=Os11g0544800 PE=3 SV=1	2.169981917	1	1	2800000	0.062	1
Q0IUM6	Os11g0147800 protein OS=Oryza sativa subsp. japonica OX=39947 GN=Os11g0147800 PE=4 SV=1	9.282700422	2	2	2400000	0.425	2
A0A0P0XX3(	Os10g0530500 protein OS=Oryza sativa subsp. japonica OX=39947 GN=Os10g0530500 PE=4 SV=1	67.2	13	102	6100000	128.155	13
Q7G7F8	Os10g0530500 protein OS=Oryza sativa subsp. japonica OX=39947 GN=Os10g0530500 PE=3 SV=1	75.96566524	21	174	27000000000	999	21
Q0IW72	Os10g0529700 protein (Fragment) OS=Oryza sativa subsp. japonica OX=39947 GN=Os10g0529700 PE=4 SV=1	15.6	4	6	3300000	0.668	4
C7J7M6	Os10g0527601 protein OS=Oryza sativa subsp. japonica OX=39947 GN=Os10g0527601 PE=4 SV=1	7.392996109	2	2		0.334	2
A0A0N7KS2	Os10g0509200 protein OS=Oryza sativa subsp. japonica OX=39947 GN=Os10g0509200 PE=4 SV=1	16.84210526	4	4	7900000	0.624	4
Q0J128	Os09g0467200 protein OS=Oryza sativa subsp. japonica OX=39947 GN=Os09g0467200 PE=3 SV=1	45.29147982	10	20	290000000	12.594	10
Q0J294	Os09g0367700 protein (Fragment) OS=Oryza sativa subsp. japonica OX=39947 GN=Os09g0367700 PE=4 SV=1	41.63090129	10	69	7900000000	12.594	10
Q6Z1P3	Os08g0566400 protein OS=Oryza sativa subsp. japonica OX=39947 GN=Os08g0566400 PE=2 SV=1	7.035175879	2	2	2800000	0.259	2
Q0J3Q9	Os08g0558200 protein OS=Oryza sativa subsp. japonica OX=39947 GN=Os08g0558200 PE=4 SV=1	3.519061584	1	1	30000000	0.166	1
A0A0P0XBM	Os08g0109200 protein (Fragment) OS=Oryza sativa subsp. japonica OX=39947 GN=Os08g0109200 PE=4 SV=1	2.336448598	1	1	6100000	0.096	1
A0A0P0X6V	Os07g0509800 protein (Fragment) OS=Oryza sativa subsp. japonica OX=39947 GN=Os07g0509800 PE=4 SV=1	3.354297694	1	1	5900000	0.083	1
A0A0P0X3G	Os07g0195100 protein (Fragment) OS=Oryza sativa subsp. japonica OX=39947 GN=Os07g0195100 PE=4 SV=1	26.37362637	5	6	7700000	0.833	5
A3BFU9	Os07g0108300 protein OS=Oryza sativa subsp. japonica OX=39947 GN=P0585H11.115 PE=4 SV=1	40.20618557	14	23	110000000	2.398	14
Q0DAL0	Os06g0646500 protein (Fragment) OS=Oryza sativa subsp. japonica OX=39947 GN=Os06g0646500 PE=3 SV=1	19.92619926	4	5	5200000	0.73	4
A0A0P0WY(	Os06g0597900 protein OS=Oryza sativa subsp. japonica OX=39947 GN=Os06g0597900 PE=3 SV=1	3.674540682	1	1	1100000	0.093	1
A0A0P0WX(	Os06g0550000 protein (Fragment) OS=Oryza sativa subsp. japonica OX=39947 GN=Os06g0550000 PE=3 SV=1	12.06030151	2	2	11000000	0.425	2
A0A0P0WR1	Os05g0564400 protein OS=Oryza sativa subsp. japonica OX=39947 GN=Os05g0564400 PE=4 SV=1	24.88479263	5	5	4900000	0.968	5
Q0DG76	Os05g0549100 protein OS=Oryza sativa subsp. japonica OX=39947 GN=Os05g0549100 PE=4 SV=1	16.45796064	7	8	37000000	0.693	7
A0A0N7KKH	Os05g0299200 protein OS=Oryza sativa subsp. japonica OX=39947 GN=Os05g0299200 PE=4 SV=1	6.653225806	2	2	9000000	0.172	2
A0A0P0WIQ	Os05g0164100 protein OS=Oryza sativa subsp. japonica OX=39947 GN=Os05g0164100 PE=4 SV=1	17.59379043	11	12	19000000	0.734	11
Q5WMY3	Os05g0155100 protein OS=Oryza sativa subsp. japonica OX=39947 GN=Os05g0155100 PE=2 SV=1	12.23404255	2	2	2200000	0.585	2
Q0DLH7	Os05g0102000 protein OS=Oryza sativa subsp. japonica OX=39947 GN=Os05g0102000 PE=4 SV=1	2.949061662	1	1	1200000	0.11	1
Q7XQV1	Os04g0640500 protein OS=Oryza sativa subsp. japonica OX=39947 GN=Os04g0640500 PE=2 SV=2	1.666666667	1	1	970000	0.062	1
A0A0P0WCZ	Os04g0542900 protein OS=Oryza sativa subsp. japonica OX=39947 GN=Os04g0542900 PE=3 SV=1	2.312138728	1	1		0.072	1
Q0JCD0	Os04g0479200 protein (Fragment) OS=Oryza sativa subsp. japonica OX=39947 GN=Os04g0479200 PE=3 SV=1	2.409638554	1	1	1300000	0.122	1
A0A0N7KIF2	Os03g0856500 protein OS=Oryza sativa subsp. japonica OX=39947 GN=Os03g0856500 PE=4 SV=1	11.97411003	2	2	16000000	0.274	2
Q10MY4	Os03g0289400 protein OS=Oryza sativa subsp. japonica OX=39947 GN=LOC_Os03g18020 PE=4 SV=1	6.161137441	1	1	2400000	0.194	1
Q0DU42	Os03g0208900 protein OS=Oryza sativa subsp. japonica OX=39947 GN=Os03g0208900 PE=4 SV=1	3.614457831	1	1		0.105	1
A0A0P0VTX	Os03g0182600 protein OS=Oryza sativa subsp. japonica OX=39947 GN=Os03g0182600 PE=3 SV=1	15.31531532	1	1	27000000	0.334	1
A0A0P0VT8	Os03g0144700 protein (Fragment) OS=Oryza sativa subsp. japonica OX=39947 GN=Os03g0144700 PE=3 SV=1	2.387267905	1	1	5100000	0.122	1
A0A0P0VSE	Os03g0122200 protein (Fragment) OS=Oryza sativa subsp. japonica OX=39947 GN=Os03g0122200 PE=3 SV=1	6.222222222	1	1	3500000	0.145	1
A0A0P0VNK	Os02g0708100 protein OS=Oryza sativa subsp. japonica OX=39947 GN=Os02g0708100 PE=3 SV=1	4.4397463	2	2	5200000	0.212	2
Q6YVH6	Os02g0705100 protein OS=Oryza sativa subsp. japonica OX=39947 GN=Os02g0705100 PE=2 SV=1	8.048780488	1	1	1200000	0.145	1
Q6K1X5	Os02g0608900 protein OS=Oryza sativa subsp. japonica OX=39947 GN=Os02g0608900 PE=2 SV=1	8.918918919	3	3	3700000	0.35	3
Q6K919	Os02g0596000 protein OS=Oryza sativa subsp. japonica OX=39947 GN=Os02g0596000 PE=4 SV=1	9.931506849	2	3	16000000	0.438	2
Q0DZW0	Os02g0595500 protein OS=Oryza sativa subsp. japonica OX=39947 GN=Os02g0595500 PE=3 SV=1	5.026455026	1	1	11000000	0.136	1
Q0E446	Os02g0137200 protein (Fragment) OS=Oryza sativa subsp. japonica OX=39947 GN=Os02g0137200 PE=3 SV=1	18.97810219	4	5	30000000	0.968	4
Q94DL4	Os01g0964133 protein OS=Oryza sativa subsp. japonica OX=39947 GN=Os01g0964133 PE=2 SV=1	26.79045093	6	8	9700000	0.957	6
A2ZXU2	Os01g0749200 protein OS=Oryza sativa subsp. japonica OX=39947 GN=Os01g0749200 PE=3 SV=1	16.30901288	3	4	11000000	0.719	3
Q0JLB5	Os01g0611000 protein (Fragment) OS=Oryza sativa subsp. japonica OX=39947 GN=Os01g0611000 PE=4 SV=1	2.255639098	1	1	840000	0.105	1

Q8LJ81	Os01g0581300 protein OS=Oryza sativa subsp. japonica OX=39947 GN=Os01g0581300 PE=2 SV=1	1.851851852	1	1	3500000	0.08	1
A0A0E0FYU	OMPdecase OS=Oryza nivara OX=4536 PE=3 SV=1	1.500535906	1	1	2200000	0.053	1
Q10CT2	Obg-like ATPase 1 OS=Oryza sativa subsp. japonica OX=39947 GN=OSJNBa0091J19.9 PE=3 SV=1	12.76102088	4	4	6400000	0.468	4
A0A0E0GKF	NTP_transferase domain-containing protein OS=Oryza nivara OX=4536 PE=4 SV=1	3.047091413	1	1	1300000	0.129	1
A0A0E0IFL1	NmrA domain-containing protein OS=Oryza nivara OX=4536 PE=4 SV=1	19.68911917	6	6	47000000	0.738	6
Q259D2	NADPH-protochlorophyllide oxidoreductase OS=Oryza sativa OX=4530 GN=H0801D08.7 PE=3 SV=1	9.560723514	2	2	6600000	0.179	2
A0A0E0H91	NAD(P)H dehydrogenase subunit H OS=Oryza nivara OX=4536 PE=3 SV=1	17.83088235	7	8	35000000	0.887	7
B9FF00	NAD(P)-bd_dom domain-containing protein OS=Oryza sativa subsp. japonica OX=39947 GN=OsJ_14672 PE=4 SV=1	9.356725146	2	4	11000000	0.668	2
A0A0E0HC0	NAD(P)-bd_dom domain-containing protein OS=Oryza nivara OX=4536 PE=4 SV=1	22.52747253	9	9	21000000	0.725	9
B9F1E6	MSP domain-containing protein OS=Oryza sativa subsp. japonica OX=39947 GN=OsJ_07762 PE=3 SV=1	1.805054152	1	2		0.077	1
Q53PA7	Mitochondrial carrier protein, expressed OS=Oryza sativa subsp. japonica OX=39947 GN=LOC_Os11g24450 PE=2 SV=1	16.18122977	4	4	18000000	0.624	4
A0A0E0GPP	Mg-protoporphyrin IX chelatase OS=Oryza nivara OX=4536 PE=3 SV=1	7.951807229	3	3	8000000	0.304	3
A0A0E0IXV2	MFS domain-containing protein OS=Oryza nivara OX=4536 PE=3 SV=1	1.113172542	1	1	2600000	0.032	1
B8B332	Methionyl-tRNA synthetase OS=Oryza sativa subsp. indica OX=39946 GN=OsI_23153 PE=3 SV=1	1.865671642	1	1	1800000	0.053	1
Q0DKP1	Methenyltetrahydrofolate cyclohydrolase (Fragment) OS=Oryza sativa subsp. japonica OX=39947 GN=Os05g0150800 PE=3 SV=1	24.31610942	6	6	41000000	0.823	6
A0A0E0GUE	Malate dehydrogenase OS=Oryza nivara OX=4536 PE=3 SV=1	9.719222462	3	3	9900000	0.16	3
A0A0E0IFV5	Malate dehydrogenase (NADP(+)) OS=Oryza nivara OX=4536 PE=3 SV=1	6.04288499	2	2	3600000	0.141	2
A0A0E0FK4	Magnesium-protoporphyrin IX monomethyl ester (oxidative) cyclase OS=Oryza nivara OX=4536 PE=3 SV=1	33.25791855	15	16	74000000	2.562	15
A3AHG6	Magnesium chelatase OS=Oryza sativa subsp. japonica OX=39947 GN=OsJ_10668 PE=3 SV=1	1.180555556	1	1	1300000	0.028	1
A0A4R1FGU	Lysine--tRNA ligase OS=Dermacoccus sp. SAI-028 OX=2768432 GN=lysS PE=3 SV=1	0.704845815	1	1	340000	0.032	1
B1PYP0	Lipoxygenase OS=Oryza sativa subsp. japonica OX=39947 GN=LOX2 PE=2 SV=1	4.121475054	3	3	3300000	0.142	3
A0A0E0G38	Lipoxygenase OS=Oryza nivara OX=4536 PE=3 SV=1	1.187904968	1	1	3600000	0.042	1
A0A0E0G8B	Lipase_3 domain-containing protein OS=Oryza nivara OX=4536 PE=4 SV=1	4.545454545	2	2	180000	0.16	2
A0A0E0GLX	Lactamase_B domain-containing protein OS=Oryza nivara OX=4536 PE=3 SV=1	3.603603604	1	1	1200000	0.155	1
A0A0E0H2K	KH type-2 domain-containing protein OS=Oryza nivara OX=4536 PE=3 SV=1	12.54019293	3	4	31000000	0.413	3
A2YMD1	Isopentenyl-diphosphate Delta-isomerase OS=Oryza sativa subsp. indica OX=39946 GN=OsI_26387 PE=3 SV=1	1.800554017	1	1	1100000	0.047	1
B8AJH5	Isoleucyl-tRNA synthetase OS=Oryza sativa subsp. indica OX=39946 GN=OsI_09155 PE=3 SV=1	1.228733459	1	1	1500000	0.038	1
A0A0E0FK0	Isocitrate dehydrogenase [NAD] subunit, mitochondrial OS=Oryza nivara OX=4536 PE=3 SV=1	8.695652174	3	3	6500000	0.318	3
A0A0E0H2Z	Isocitrate dehydrogenase (NADP(+)) OS=Oryza nivara OX=4536 PE=3 SV=1	2.226027397	1	1	7400000	0.058	1
A0A0E0G20	Inositol-1-monophosphatase OS=Oryza nivara OX=4536 PE=3 SV=1	2.459016393	1	1	3500000	0.136	1
A0A0E0JBK	Histone H4 OS=Oryza nivara OX=4536 PE=3 SV=1	11.65048544	1	1	1900000	0.468	1
A3BFF4	HATPase_c domain-containing protein OS=Oryza sativa subsp. japonica OX=39947 GN=OsJ_22671 PE=3 SV=1	1.193317422	1	1	700000	0.045	1
A0A0E0JCQ	HATPase_c domain-containing protein OS=Oryza nivara OX=4536 PE=3 SV=1	3.789836348	3	3	6400000	0.098	3
A0A0E0GX2	HATPase_c domain-containing protein OS=Oryza nivara OX=4536 PE=3 SV=1	3.413940256	2	2	7900000	0.105	2
A0A0E0GV0	H15 domain-containing protein OS=Oryza nivara OX=4536 PE=3 SV=1	2.666666667	1	1	5600000	0.089	1
A0A5C8LKB	GTP-binding protein (Fragment) OS=Rheinheimera tangshanensis OX=400153 GN=FU839_18715 PE=4 SV=1	21.9858156	2	3		1.371	2
A2Y0P2	GTP diphosphokinase OS=Oryza sativa subsp. indica OX=39946 GN=OsI_18566 PE=3 SV=1	2.325581395	1	1	2400000	0.07	1
A0A0E0HP6	Glycosyltransferase OS=Oryza nivara OX=4536 PE=3 SV=1	3.105590062	1	1	12000000	0.089	1
A0A0E0FT6	Glyco_hydro_18 domain-containing protein OS=Oryza nivara OX=4536 PE=4 SV=1	4.04040404	1	1	2100000	0.166	1
A2ZX46	Glycine cleavage system P protein OS=Oryza sativa subsp. japonica OX=39947 GN=OsJ_03218 PE=3 SV=1	2.70531401	2	2	9400000	0.091	2
A2YQT7	Glyceraldehyde-3-phosphate dehydrogenase, cytosolic OS=Oryza sativa subsp. indica OX=39946 GN=GAPC PE=2 SV=1	39.46587537	9	19	190000000	3.329	9
A3AV14	Glyceraldehyde-3-phosphate dehydrogenase OS=Oryza sativa subsp. japonica OX=39947 GN=OsJ_15252 PE=3 SV=1	45.71428571	13	30	140000000	5.918	13
Q7X8A1	Glyceraldehyde-3-phosphate dehydrogenase OS=Oryza sativa subsp. japonica OX=39947 GN=Os04g0459500 PE=2 SV=1	46.76616915	17	57	2700000000	13.454	17
Q9SNK3	Glyceraldehyde-3-phosphate dehydrogenase OS=Oryza sativa subsp. japonica OX=39947 GN=OJ1528D07.7 PE=3 SV=1	33.33333333	15	40	1400000000	4.736	15
B8AF09	Glyceraldehyde-3-phosphate dehydrogenase OS=Oryza sativa subsp. indica OX=39946 GN=OsI_07948 PE=3 SV=1	35.6741573	10	23	56000000	3.786	10
A2WYR9	Glutathione transferase OS=Oryza sativa subsp. indica OX=39946 GN=OsI_05077 PE=3 SV=1	5.150214592	1	1	4900000	0.194	1
Q6WSC2	Glutathione S-transferase OS=Oryza sativa subsp. indica OX=39946 GN=gstu4 PE=2 SV=1	30.90128755	9	20	92000000	5.661	9

Q7G227	Glutathione S-transferase GSTU6, putative, expressed OS=Oryza sativa subsp. japonica OX=39947 GN=LOC_Os10g38160 PE=3 SV=	17.68953069	5	14		1.783	5
A0A0E0HQ0	Glutathione peroxidase OS=Oryza nivara OX=4536 PE=3 SV=1	4.60251046	1	1	2100000	0.179	1
A2X1X4	Glutaredoxin-dependent peroxiredoxin OS=Oryza sativa subsp. indica OX=39946 GN=OsI_06200 PE=3 SV=1	9.777777778	2	2	7200000	0.425	2
A0A0E0J7F4	Glutaredoxin domain-containing protein OS=Oryza nivara OX=4536 PE=3 SV=1	3.859649123	1	1	4400000	0.155	1
A0A0E0I8K6	Glutamyl-tRNA(Gln) amidotransferase subunit A, chloroplastic/mitochondrial OS=Oryza nivara OX=4536 GN=GATA PE=3 SV=1	6.206896552	2	2	16000000	0.16	2
A0A0E0H6Y	Glutamine synthetase OS=Oryza nivara OX=4536 PE=3 SV=1	29.66292135	10	14	160000000	3.329	10
B9FUN5	Glutamine amidotransferase type-2 domain-containing protein OS=Oryza sativa subsp. japonica OX=39947 GN=OsJ_25434 PE=3 S	1.410177805	2	2	3300000	0.05	2
A0A0E0IF06	Glutamate-1-semialdehyde 2,1-aminomutase OS=Oryza nivara OX=4536 PE=3 SV=1	32.42677824	10	12	120000000	1.754	10
A0A542RWI	Glutamate dehydrogenase OS=Nocardioides sp. SLBN-35 OX=2768445 GN=FBY25_2670 PE=3 SV=1	2.684563758	1	1	15000000	0.072	1
Q8LLP2	Glutamate decarboxylase OS=Oryza sativa subsp. japonica OX=39947 GN=OSJNBa0031O09.06 PE=3 SV=1	3.118908382	1	2	4300000	0.086	1
A0A0E0GLM	Glucose-6-phosphate 1-dehydrogenase OS=Oryza nivara OX=4536 PE=3 SV=1	2.663115846	2	2	5100000	0.105	2
A0A0E0H7V	Glucose-1-phosphate adenylyltransferase OS=Oryza nivara OX=4536 PE=3 SV=1	1.567944251	1	1	6400000	0.059	1
Q6AVT2	Glucose-1-phosphate adenylyltransferase large subunit 1, chloroplastic/amyloplastic OS=Oryza sativa subsp. japonica OX=39947 G	8.023483366	4	4	2900000	0.199	4
A0A0E0ICZ8	Germin-like protein OS=Oryza nivara OX=4536 PE=3 SV=1	15.96244131	3	6	51000000	5.813	3
A0A0E0HLA	Geranylgeranyl reductase OS=Oryza nivara OX=4536 PE=3 SV=1	41.03671706	13	17	83000000	3.262	13
A2ZBX1	Fructose-bisphosphate aldolase OS=Oryza sativa subsp. indica OX=39946 GN=OsI_35277 PE=3 SV=1	19.58762887	5	9	51000000	1.031	5
A0A1L2JKJ5	Fructose-bisphosphate aldolase OS=Oryza sativa OX=4530 PE=2 SV=1	16.20111732	5	5	2300000	0.73	5
A0A0E0HTC	Fructose-bisphosphate aldolase OS=Oryza nivara OX=4536 PE=3 SV=1	9.880749574	5	5	11000000	0.45	5
A0A0E0HE7	Fructose-bisphosphate aldolase OS=Oryza nivara OX=4536 PE=3 SV=1	11.45251397	4	4		0.52	4
A2YQL4	Fructokinase-2 OS=Oryza sativa subsp. indica OX=39946 GN=FRK2 PE=1 SV=2	3.273809524	1	1	4200000	0.116	1
A0A0E0HRV	Flavin-containing monooxygenase OS=Oryza nivara OX=4536 PE=3 SV=1	2.268041237	1	1	1100000	0.072	1
Q0JJS8	Fe-S cluster assembly factor HCF101, chloroplastic OS=Oryza sativa subsp. japonica OX=39947 GN=HCF101 PE=3 SV=3	2.071563089	1	1	2600000	0.101	1
A0A0E0FZU	Ferredoxin--NADP reductase, chloroplastic OS=Oryza nivara OX=4536 PE=3 SV=1	16.66666667	4	5	23000000	0.509	4
A0A0E0HJQ	Ferredoxin--NADP reductase, chloroplastic OS=Oryza nivara OX=4536 PE=3 SV=1	12.93532338	4	4	15000000	0.389	4
A2YLG4	FAD_binding_3 domain-containing protein OS=Oryza sativa subsp. indica OX=39946 GN=OsI_26059 PE=3 SV=1	5.676855895	2	2	2900000	0.16	2
A0A0E0IS47	Epimerase domain-containing protein OS=Oryza nivara OX=4536 PE=4 SV=1	28.04232804	9	13	34000000	1.512	9
A0A0E0H6D	Epimerase domain-containing protein OS=Oryza nivara OX=4536 PE=4 SV=1	7.225433526	2	2	3800000	0.259	2
A0A0E0HY4	Epimerase domain-containing protein OS=Oryza nivara OX=4536 PE=4 SV=1	3.307888041	1	1	3600000	0.101	1
A2XMW9	ENT domain-containing protein OS=Oryza sativa subsp. indica OX=39946 GN=OsI_13894 PE=3 SV=1	9.05752754	5	5	4300000	0.259	5
A0A542V9L	Elongation factor Tu OS=Pseudomonas sp. SLBN-26 OX=2768443 GN=tuf PE=3 SV=1	6.801007557	2	3	30000000	0.304	2
B8AEQ9	Elongation factor Tu OS=Oryza sativa subsp. indica OX=39946 GN=OsI_07899 PE=3 SV=1	39.13894325	15	31	730000000	3.417	15
A0A0E0GWI	Elongation factor Tu OS=Oryza nivara OX=4536 PE=3 SV=1	20.08830022	6	7	13000000	0.711	6
A2XVY3	Elongation factor G, chloroplastic OS=Oryza sativa subsp. indica OX=39946 GN=OsI_16800 PE=3 SV=1	15.63307494	8	9	17000000	0.456	8
Q10QZ4	Elongation factor 1-alpha OS=Oryza sativa subsp. japonica OX=39947 GN=LOC_Os03g08060 PE=3 SV=1	34.07572383	10	19	210000000	1.807	10
A0A0E0GVT	Dolichyl-phosphate beta-D-mannosyltransferase OS=Oryza nivara OX=4536 PE=4 SV=1	1.890034364	1	2	6300000	0.066	1
A0A0E0GMI	Divinyl chlorophyllide a 8-vinyl-reductase, chloroplastic OS=Oryza nivara OX=4536 PE=4 SV=1	10.86419753	3	3	6400000	0.389	3
A0A0E0J4Y3	Dirigent protein OS=Oryza nivara OX=4536 PE=3 SV=1	7.5	1	1	1600000	0.389	1
A2XYX9	Dihydroorotate dehydrogenase (quinone), mitochondrial OS=Oryza sativa subsp. indica OX=39946 GN=OsI_17913 PE=3 SV=1	2.558635394	1	1	4200000	0.093	1
A3ATC6	Dihydrolipoyllysine-residue succinyltransferase OS=Oryza sativa subsp. japonica OX=39947 GN=Os04g0394200 PE=3 SV=1	9.545454545	2	2	4500000	0.222	2
A0A0E0FL8	Dihydrolipoyl dehydrogenase OS=Oryza nivara OX=4536 PE=3 SV=1	2.317290553	1	1	5100000	0.083	1
A0A0E0ILV6	Dihydrolipoamide acetyltransferase component of pyruvate dehydrogenase complex OS=Oryza nivara OX=4536 PE=3 SV=1	6.145251397	3	3	16000000	0.199	3
A0A0E0FKX	Dihydrolipoamide acetyltransferase component of pyruvate dehydrogenase complex OS=Oryza nivara OX=4536 PE=3 SV=1	4.206500956	1	1	7500000	0.08	1
A0A0E0IJD8	Dihydrolipoamide acetyltransferase component of pyruvate dehydrogenase complex OS=Oryza nivara OX=4536 PE=3 SV=1	2.247191011	1	1	7700000	0.08	1
A0A0E0IC70	Dihydrolipoamide acetyltransferase component of pyruvate dehydrogenase complex OS=Oryza nivara OX=4536 PE=3 SV=1	2.066115702	1	1	2500000	0.096	1
A0A0E0IN07	DHQ_synthase domain-containing protein OS=Oryza nivara OX=4536 PE=3 SV=1	4.288939052	2	2	7600000	0.186	2
B9EUU5	D-fructose-1,6-bisphosphate 1-phosphohydrolase OS=Oryza sativa subsp. japonica OX=39947 GN=OsJ_04198 PE=3 SV=1	16.17021277	6	6	20000000	0.585	6
A0A0E0GKD	D-fructose-1,6-bisphosphate 1-phosphohydrolase OS=Oryza nivara OX=4536 PE=3 SV=1	17.22488038	5	7	16000000	1.069	5

A0A0E0HV2	Delta-aminolevulinic acid dehydratase OS=Oryza nivara OX=4536 PE=3 SV=1	2.02020202	1	1	1900000	0.086	1
A0A0B4U0L	Dehydrogenase (Fragment) OS=Oryza sativa OX=4530 GN=LOC_Os01g53910.1 PE=2 SV=1	3.779069767	1	1	2400000	0.136	1
A2YR99	DAGKc domain-containing protein OS=Oryza sativa subsp. indica OX=39946 GN=OsI_27828 PE=4 SV=1	2.360515021	1	1	2800000	0.093	1
A0A0P0WZL	D-3-phosphoglycerate dehydrogenase OS=Oryza sativa subsp. japonica OX=39947 GN=Os06g0655100 PE=3 SV=1	2.05371248	1	1	1900000	0.066	1
A0A0E0H6Q	D-3-phosphoglycerate dehydrogenase OS=Oryza nivara OX=4536 PE=3 SV=1	2.120717781	1	1	5000000	0.072	1
A0A0E0GE0	CYTOSOL_AP domain-containing protein OS=Oryza nivara OX=4536 PE=3 SV=1	4.013377926	2	2	3500000	0.15	2
A0A0E0FMY	Cytochrome f OS=Oryza nivara OX=4536 PE=3 SV=1	4.459691252	2	2	11000000	0.133	2
Q7XLG4	Cytochrome b-c1 complex subunit Rieske, mitochondrial OS=Oryza sativa subsp. japonica OX=39947 GN=Os04g0398500 PE=2 SV=	6.115107914	1	1	9700000	0.145	1
A3AQX8	Cysteine synthase OS=Oryza sativa subsp. japonica OX=39947 GN=OsJ_13780 PE=3 SV=1	3.10880829	1	1	4200000	0.11	1
A0A0E0J938	Cysteine desulfurase OS=Oryza nivara OX=4536 PE=4 SV=1	15.56503198	5	5	13000000	0.403	5
A0A0E0IHY0	Cysteine desulfurase OS=Oryza nivara OX=4536 PE=3 SV=1	2.036659878	1	1	2200000	0.083	1
A0A0E0IXJ3	CTP:phosphoethanolamine cytidylyltransferase OS=Oryza nivara OX=4536 PE=3 SV=1	2.764976959	1	1	5000000	0.093	1
B8B8S6	CpSecY OS=Oryza sativa subsp. indica OX=39946 GN=OsI_28473 PE=3 SV=1	0.779220779	1	1	6300000	0.042	1
A0A0E0H5U	Coproporphyrinogen oxidase OS=Oryza nivara OX=4536 PE=3 SV=1	5.25	2	2	5600000	0.172	2
A0A0E0HVM	Component of oligomeric Golgi complex 7 OS=Oryza nivara OX=4536 PE=3 SV=1	9.76744186	7	7	21000000	0.302	7
A0A0E0H5V	Coatomer subunit epsilon OS=Oryza nivara OX=4536 PE=3 SV=1	3.484320557	1	1	2000000	0.129	1
A0A7S7YAC0	Clp protease proteolytic subunit OS=Oryza sativa temperate japonica subgroup OX=1736657 GN=clpP PE=4 SV=1	25	4	7	13000000	3.642	4
A0A5C8LLI6	Citrate synthase OS=Rheinheimera tangshanensis OX=400153 GN=gltA PE=3 SV=1	3.294117647	1	1	10000000	0.101	1
B9G3G3	Cinnamyl-alcohol dehydrogenase OS=Oryza sativa subsp. japonica OX=39947 GN=OsJ_29281 PE=3 SV=1	9.817351598	3	3	4900000	0.35	3
B8B9C5	Cinnamyl-alcohol dehydrogenase OS=Oryza sativa subsp. indica OX=39946 GN=OsI_30129 PE=3 SV=1	8.423913043	3	3	12000000	0.389	3
A0A0E0FI37	Cinnamyl-alcohol dehydrogenase OS=Oryza nivara OX=4536 PE=3 SV=1	2.732240437	1	1	4500000	0.136	1
A0A172MA9	Chloroplast 9-cis-epoxycarotenoid dioxygenase 1 OS=Oryza sativa subsp. indica OX=39946 PE=2 SV=1	7.680250784	3	3	4200000	0.218	3
Q10HD0	Chlorophyll a-b binding protein, chloroplastic OS=Oryza sativa subsp. japonica OX=39947 GN=RCABP89 PE=2 SV=1	16.73003802	5	10	73000000	2.162	5
Q6Z411	Chlorophyll a-b binding protein, chloroplastic OS=Oryza sativa subsp. japonica OX=39947 GN=RCABP69 PE=2 SV=1	8.620689655	2	2	48000000	0.389	2
Q53N83	Chlorophyll a-b binding protein, chloroplastic OS=Oryza sativa subsp. japonica OX=39947 GN=LOC_Os11g13890 PE=2 SV=1	44.87632509	9	24	290000000	9	9
A2WUJ5	Chlorophyll a-b binding protein, chloroplastic OS=Oryza sativa subsp. indica OX=39946 GN=OsI_03546 PE=3 SV=1	6.037735849	2	3	8500000	0.468	2
O22542	Chlorophyll a-b binding protein, chloroplastic OS=Oryza sativa OX=4530 PE=2 SV=1	5.592105263	1	1	7800000	0.122	1
Q01JQ3	Chlorophyll a-b binding protein, chloroplastic OS=Oryza sativa OX=4530 GN=H0523F07.10 PE=3 SV=1	5.158730159	1	1	5600000	0.179	1
A0A0E0ICB1	Chlorophyll a-b binding protein, chloroplastic OS=Oryza nivara OX=4536 PE=3 SV=1	5.737704918	1	1	17000000	0.194	1
A6N104	Chlorophyll a-b binding protein, chloroplastic (Fragment) OS=Oryza sativa subsp. indica OX=39946 PE=2 SV=1	39.79057592	6	9	210000000	12.335	6
A0A0E0GV6	CCT-theta OS=Oryza nivara OX=4536 PE=3 SV=1	2.456140351	1	1	3600000	0.058	1
A0A0E0FTZ3	Catalase OS=Oryza nivara OX=4536 PE=3 SV=1	8.536585366	4	4	6300000	0.374	4
B8AME2	Catalase isozyme C OS=Oryza sativa subsp. indica OX=39946 GN=OsI_09857 PE=3 SV=1	9.146341463	4	5	10000000	0.509	4
Q0JKY8	Carbonic anhydrase (Fragment) OS=Oryza sativa subsp. japonica OX=39947 GN=Os01g0639900 PE=3 SV=1	41.63701068	10	24	540000000	5.952	10
B9EXM2	Carbamoyl-phosphate synthase large chain, chloroplastic OS=Oryza sativa subsp. japonica OX=39947 GN=CARB PE=2 SV=1	9.300341297	8	8	8700000	0.301	8
A2Z3W4	C2 NT-type domain-containing protein OS=Oryza sativa subsp. indica OX=39946 GN=OsI_32329 PE=4 SV=1	5.889014723	3	3	5500000	0.124	3
A0A0E0GJV8	Beta-galactosidase OS=Oryza nivara OX=4536 PE=3 SV=1	1.248699272	1	1	2400000	0.058	1
Q5NBF5	ATP-dependent Clp protease proteolytic subunit OS=Oryza sativa subsp. japonica OX=39947 GN=OsJ_01248 PE=3 SV=1	16.33986928	5	5	19000000	0.896	5
Q0DRY8	ATP-dependent Clp protease proteolytic subunit OS=Oryza sativa subsp. japonica OX=39947 GN=Os03g0344900 PE=3 SV=1	8.309455587	2	2	19000000	0.245	2
A2X7K2	ATP-dependent Clp protease proteolytic subunit OS=Oryza sativa subsp. indica OX=39946 GN=OsI_08189 PE=3 SV=1	6.918238994	1	1	91000000	0.194	1
A0A0E0HJ81	ATP-dependent Clp protease proteolytic subunit OS=Oryza nivara OX=4536 PE=3 SV=1	6.735751295	2	3	13000000	0.222	2
A2ZH35	ATPase_AAA_core domain-containing protein OS=Oryza sativa subsp. indica OX=39946 GN=OsI_37097 PE=4 SV=1	13.33333333	3	5	25000000	0.557	3
B8AVM5	ATPase_AAA_core domain-containing protein OS=Oryza sativa subsp. indica OX=39946 GN=OsI_17750 PE=4 SV=1	8.552631579	3	4	9000000	0.346	3
A0A5S6R9N	ATP synthase subunit beta OS=Oryza sativa subsp. japonica OX=39947 GN=OSJNBb0075K12.38 PE=3 SV=1	26.90763052	9	11	69000000	1.395	9
A0A0E0HHV	ATP synthase subunit beta OS=Oryza nivara OX=4536 PE=3 SV=1	1.037037037	1	1	7500000	0.032	1
A0A0N9E0X	ATP synthase subunit b, chloroplastic OS=Oryza sativa tropical japonica subgroup OX=1736656 GN=atpF PE=3 SV=1	10.10638298	2	2	6300000	0.425	2
E9KIN8	ATP synthase subunit alpha, chloroplastic OS=Oryza sativa subsp. japonica OX=39947 GN=atpA PE=3 SV=1	39.58724203	17	23	180000000	2.652	17

A0A0E0G13	ATP synthase subunit alpha OS=Oryza nivara OX=4536 PE=3 SV=1	17.82682513	9	12	56000000	0.778	9
Q8HCR5	ATP synthase protein MI25 OS=Oryza sativa subsp. japonica OX=39947 GN=orf25 PE=3 SV=2	5.583756345	1	1	2100000	0.194	1
B8BMJ6	ATP citrate synthase OS=Oryza sativa subsp. indica OX=39946 GN=OsI_38756 PE=3 SV=1	10.72210066	4	4	5400000	0.52	4
A0A542P23	Aspartate carbamoyltransferase OS=Arthrobacter sp. SLBN-53 OX=2768412 GN=pyrB PE=3 SV=1	3.144654088	1	1	78000000	0.145	1
Q8GTK9	Asparagine--tRNA ligase OS=Oryza sativa subsp. japonica OX=39947 GN=OJ1753_E03.117 PE=3 SV=1	1.712328767	1	1	7100000	0.072	1
V5K4S9	Asparagine synthetase [glutamine-hydrolyzing] OS=Oryza sativa subsp. japonica OX=39947 PE=2 SV=1	3.045685279	1	1	2000000	0.07	1
B8BF46	Annexin OS=Oryza sativa subsp. indica OX=39946 GN=OsI_31267 PE=3 SV=1	7.449856734	2	2	7700000	0.194	2
B7ENR4	Aminomethyltransferase (Fragment) OS=Oryza sativa subsp. japonica OX=39947 PE=2 SV=1	12.22493888	4	5	4100000	0.509	4
Q8RUM9	Amidophosphoribosyltransferase OS=Oryza sativa subsp. japonica OX=39947 GN=OSJNBb0008G24.26 PE=3 SV=1	1.996370236	1	1	2900000	0.077	1
B8ACF5	Alpha-1,4 glucan phosphorylase OS=Oryza sativa subsp. indica OX=39946 GN=OsI_04460 PE=3 SV=1	1.545778835	1	1	1300000	0.049	1
A3C1M3	Aldo_ket_red domain-containing protein OS=Oryza sativa subsp. japonica OX=39947 GN=OsJ_30388 PE=4 SV=1	6.540084388	3	3	6500000	0.25	3
A0A0E0IZ66	Aldehyde dehydrogenase OS=Oryza nivara OX=4536 PE=3 SV=1	6.072874494	2	2	14000000	0.202	2
A0A0P0XM	Aldehyde dehydrogenase (NAD(+)) OS=Oryza sativa subsp. japonica OX=39947 GN=OJ1344_B01.27-1 PE=3 SV=1	2.554027505	1	1	1700000	0.083	1
B9V0N7	Alcohol dehydrogenase family-1 OS=Oryza nivara OX=4536 GN=petD PE=3 SV=1	7.65171504	2	2	4000000	0.233	2
A0A0E0IE06	Alanine--glyoxylate transaminase OS=Oryza nivara OX=4536 PE=3 SV=1	31.39534884	9	10	27000000	1.424	9
B9FHE3	AIG1-type G domain-containing protein OS=Oryza sativa subsp. japonica OX=39947 GN=OsJ_17149 PE=3 SV=1	1.055011304	1	1	3000000	0.036	1
A2YW01	Aha1_N domain-containing protein OS=Oryza sativa subsp. indica OX=39946 GN=OsI_29507 PE=3 SV=1	11.87214612	4	4	11000000	0.389	4
A1YQJ8	ADP/ATP translocase OS=Oryza sativa subsp. japonica OX=39947 GN=Os02g0718900 PE=3 SV=1	16.23036649	6	7	11000000	1.081	6
A0A0N7KFQ	Adenosine kinase (Fragment) OS=Oryza sativa subsp. japonica OX=39947 GN=Os02g0625500 PE=3 SV=1	4.255319149	1	1	1900000	0.105	1
A0A0E0IS36	Acylaminoacyl-peptidase OS=Oryza nivara OX=4536 PE=3 SV=1	1.775147929	1	1	2400000	0.055	1
P0C539	Actin-2 OS=Oryza sativa subsp. indica OX=39946 GN=ACT2 PE=3 SV=1	35.80901857	8	9	11000000	1.371	8
A0A218KL3	Actin-1 OS=Oryza sativa OX=4530 PE=2 SV=1	30.23872679	7	9	49000000	1.154	7
B8AFH3	Acetohydroxy-acid synthase small subunit OS=Oryza sativa subsp. indica OX=39946 GN=OsI_08011 PE=3 SV=1	1.985559567	1	1	7500000	0.07	1
A0A0E0HYB	ABC1 domain-containing protein OS=Oryza nivara OX=4536 PE=4 SV=1	4.07079646	2	2	2600000	0.141	2
B9FIP1	AB hydrolase-1 domain-containing protein OS=Oryza sativa subsp. japonica OX=39947 GN=OsJ_18574 PE=4 SV=1	2.727272727	1	1	3600000	0.122	1
A2XY71	AB hydrolase-1 domain-containing protein OS=Oryza sativa subsp. indica OX=39946 GN=OsI_17656 PE=4 SV=1	17.09183673	4	5	18000000	0.833	4
A2XWA1	AAI domain-containing protein OS=Oryza sativa subsp. indica OX=39946 GN=OsI_16928 PE=4 SV=1	7.299270073	1	1	1700000	0.389	1
B8AM18	AAA domain-containing protein OS=Oryza sativa subsp. indica OX=39946 GN=OsI_11173 PE=3 SV=1	1.956521739	1	1	7500000	0.08	1
A0A0E0HVR	AAA domain-containing protein OS=Oryza nivara OX=4536 PE=3 SV=1	9.912536443	4	4	6100000	0.359	4
A0A0E0GDS	AAA domain-containing protein OS=Oryza nivara OX=4536 PE=3 SV=1	3.051643192	1	1	4200000	0.08	1
A2X7Z3	AA_kinase domain-containing protein OS=Oryza sativa subsp. indica OX=39946 GN=OsI_08341 PE=3 SV=1	3.757225434	1	1	2000000	0.122	1
A0A0E0IAA	60S ribosomal protein L7a OS=Oryza nivara OX=4536 PE=3 SV=1	4.089219331	1	1		0.145	1
A2X6N1	60S ribosomal protein L6 OS=Oryza sativa subsp. indica OX=39946 GN=OsI_07870 PE=3 SV=1	15.06849315	3	3	5800000	0.468	3
A0A0E0FT8	60S ribosomal protein L18a OS=Oryza nivara OX=4536 PE=3 SV=1	13.54166667	3	3	2800000	0.585	3
A2ZCQ7	60S ribosomal protein L10-1 OS=Oryza sativa subsp. indica OX=39946 GN=SC34 PE=2 SV=2	10.26785714	2	2	10000000	0.389	2
A0A0E0GG4	50S ribosomal protein L5, chloroplastic OS=Oryza nivara OX=4536 PE=3 SV=1	30.10752688	7	7	26000000	1.448	7
J7F224	50S ribosomal protein L23, chloroplastic OS=Oryza sativa subsp. indica OX=39946 GN=rpl23 PE=3 SV=1	11.11111111	1	1	3700000	0.389	1
A0A0N9E0Z	50S ribosomal protein L22, chloroplastic OS=Oryza sativa tropical japonica subgroup OX=1736656 GN=rpl22 PE=3 SV=1	8.724832215	1	1	9600000	0.334	1
Q7F4T2	50S ribosomal protein L14, chloroplastic OS=Oryza sativa subsp. japonica OX=39947 GN=rpl14 PE=3 SV=1	21.95121951	2	2	2500000	0.668	2
A0A0K0K9A	4-hydroxy-tetrahydrodipicolinate synthase OS=Oryza sativa OX=4530 GN=DHDPS PE=2 SV=1	2.631578947	1	1		0.122	1
A0A0E0GFG	4a-hydroxytetrahydrobiopterin dehydratase OS=Oryza nivara OX=4536 PE=3 SV=1	16.22807018	2	2	4800000	0.468	2
A2XHR6	40S ribosomal protein SA OS=Oryza sativa subsp. indica OX=39946 GN=OsI_11954 PE=3 SV=1	10.32258065	3	3	15000000	0.501	3
A0A5S6RDN	40S ribosomal protein S8 OS=Oryza sativa subsp. japonica OX=39947 GN=Os04g0349500 PE=3 SV=1	6.787330317	1	1	5200000	0.212	1
A2YNT9	40S ribosomal protein S6 OS=Oryza sativa subsp. indica OX=39946 GN=OsI_26912 PE=3 SV=1	6.4	1	1	2300000	0.155	1
A0A0E0FZW	40S ribosomal protein S4 OS=Oryza nivara OX=4536 PE=3 SV=1	10.56603774	3	3	3800000	0.389	3
A0A0E0J9J1	40S ribosomal protein S3a OS=Oryza nivara OX=4536 PE=3 SV=1	6.810035842	4	4	12000000	0.202	4
A2Y8X9	3-phosphoshikimate 1-carboxyvinyltransferase OS=Oryza sativa subsp. indica OX=39946 GN=EPSPS PE=2 SV=1	2.912621359	1	1	6300000	0.093	1

A0A0P0WU	3-oxoacyl-[acyl-carrier-protein] synthase OS=Oryza sativa subsp. japonica OX=39947 GN=Os06g0196600 PE=3 SV=1	13.11827957	4	4	8800000	0.492	4
A0A0H3UP3	3-ketoacyl-CoA thiolase-like protein OS=Oryza sativa subsp. japonica OX=39947 PE=2 SV=1	3.348214286	1	1	6900000	0.105	1
A6N079	30S ribosomal protein S8, chloroplastic OS=Oryza sativa subsp. indica OX=39946 GN=OsI_07208 PE=2 SV=1	6.153846154	1	1	2600000	0.233	1
A0A0E0JAH	30S ribosomal protein S4, chloroplastic OS=Oryza nivara OX=4536 PE=3 SV=1	4.615384615	1	1	14000000	0.179	1
A0A1W5HX	30S ribosomal protein S4, chloroplastic OS=Oryza grandiglumis OX=29690 GN=rps4 PE=3 SV=1	4.975124378	1	1	2100000	0.179	1
A0A0A8E0T	30S ribosomal protein S4 OS=Xanthomonas sacchari OX=56458 GN=rpsD PE=3 SV=1	3.349282297	1	1	3600000	0	1
A0A0K0LRP	30S ribosomal protein S3, chloroplastic OS=Oryza nivara OX=4536 GN=rps3 PE=3 SV=1	5.020920502	1	1	5000000	0.166	1
E9KIN4	30S ribosomal protein S2, chloroplastic OS=Oryza sativa subsp. japonica OX=39947 GN=rps2 PE=3 SV=1	9.236947791	2	2	22000000	0.311	2
Q10MB2	30S ribosomal protein S1, chloroplast, putative, expressed OS=Oryza sativa subsp. japonica OX=39947 GN=LOC_Os03g20100 PE=4	11.19402985	3	3	13000000	0.369	3
B8BLZ1	2-isopropylmalate synthase OS=Oryza sativa subsp. indica OX=39946 GN=OsI_37410 PE=3 SV=1	2.047244094	1	1	10000000	0.058	1
A2YGN8	26S proteasome non-ATPase regulatory subunit 2 homolog OS=Oryza sativa subsp. indica OX=39946 GN=OsI_24349 PE=3 SV=1	1.66481687	1	1	2700000	0.038	1
C0J9X5	26S protease regulatory subunit S10B OS=Oryza nivara OX=4536 PE=3 SV=1	9.725685786	3	3	10000000	0.292	3
O65103	23 kDa polypeptide of photosystem II OS=Oryza sativa OX=4530 PE=2 SV=2	23.22834646	4	5	20000000	0.896	4
A2WJR2	1-deoxy-D-xylulose-5-phosphate reductoisomerase OS=Oryza sativa subsp. indica OX=39946 GN=OsI_00059 PE=3 SV=1	5.285412262	2	2	4100000	0.172	2
A0A0E0GUL	(S)-2-hydroxy-acid oxidase OS=Oryza nivara OX=4536 PE=3 SV=1	15.82952816	9	9	52000000	0.995	9
A0A0E0HWI	(S)-2-hydroxy-acid oxidase OS=Oryza nivara OX=4536 PE=3 SV=1	18.69918699	5	8	26000000	1.089	5
A0A0E0I3N	(S)-2-hydroxy-acid oxidase OS=Oryza nivara OX=4536 PE=3 SV=1	3.916449086	1	1	1300000	0.075	1
