# Supplementary material for: Glutathione S-transferase activity facilitates rice tolerance to the barnyard grass root exudate DIMBOA
Source: BMC Plant Biol. 2024 Feb 17;24:117. doi: 10.1186/s12870-024-04802-5 (PMC10874003; doi:10.1186/s12870-024-04802-5)
Supplement: Supplementary file 3 — Supplementary Material 3: Fig. S3. Predicted protein‒protein interactions among differentially expressed proteins from DIMBOA-treated PI312777 and the control group. Red nodes represent upregulated proteins; blue nodes represent downregulated proteins [file 12870_2024_4802_MOESM3_ESM.pdf]

Accession	Description	Coverage	# Peptides	# PSMs	Area: F4: Sample	emPAI	# Peptides Sequest HT
Q0JG12	Os01g0949800 protein OS=Oryza sativa subsp. japonica OX=39947 GN=Os01g0949800 PE=4 SV=1	69.26406926	28	371	3800000000	49237.826	28
Q5K3B1	Ribulose bisphosphate carboxylase large chain (Fragment) OS=Oryza sativa OX=4530 GN=rbcL PE=3 SV=1	55.88235294	27	74	2500000000	202.092	27
A0A0E0FYT0	Uncharacterized protein OS=Oryza nivara OX=4536 PE=4 SV=1	18.43657817	25	330	10000000000	21.758	25
P93431	Ribulose bisphosphate carboxylase/oxygenase activase, chloroplastic OS=Oryza sativa subsp. japonica OX=39947 GN=RCA PE=1 SV=2	61.80257511	25	77	5900000000	71.79	25
Q7X8A1	Glyceraldehyde-3-phosphate dehydrogenase OS=Oryza sativa subsp. japonica OX=39947 GN=Os04g0459500 PE=2 SV=1	66.16915423	24	85	9500000000	82.176	24
Q339G9	Ribulose bisphosphate carboxylase large chain OS=Oryza sativa subsp. japonica OX=39947 GN=LOC_Os10g21280 PE=3 SV=2	54.97237569	23	67	1200000000	245.209	23
A0A0E0HGC5	Phosphoglycerate kinase OS=Oryza nivara OX=4536 PE=3 SV=1	57.90554415	22	55	2300000000	25.264	22
Q9SNK3	Glyceraldehyde-3-phosphate dehydrogenase OS=Oryza sativa subsp. japonica OX=39947 GN=OJ1528D07.7 PE=3 SV=1	51.12612613	22	63	5200000000	11.69	22
A0A0E0HIC8	Uncharacterized protein OS=Oryza nivara OX=4536 PE=4 SV=1	74.27385892	21	62	8400000000	204.353	21
A3BFU9	Os07g0108300 protein OS=Oryza sativa subsp. japonica OX=39947 GN=P0585H11.115 PE=4 SV=1	53.19587629	21	53	560000000	21.067	21
B8AEQ9	Elongation factor Tu OS=Oryza sativa subsp. indica OX=39946 GN=OsI_07899 PE=3 SV=1	53.42465753	20	41	1800000000	12.46	20
E9KIN8	ATP synthase subunit alpha, chloroplastic OS=Oryza sativa subsp. japonica OX=39947 GN=atpA PE=3 SV=1	40.15009381	20	26	610000000	4.623	20
Q7G7F8	Os10g0530500 protein OS=Oryza sativa subsp. japonica OX=39947 GN=Os10g0530500 PE=3 SV=1	59.65665236	19	77	14000000000	371.759	19
A0A0P0X1V6	Os07g0108300 protein (Fragment) OS=Oryza sativa subsp. japonica OX=39947 GN=Os07g0108300 PE=4 SV=1	55.14874142	18	42	15000000	14.317	18
P0C2Z7	ATP synthase subunit beta, chloroplastic OS=Oryza sativa OX=4530 GN=atpB PE=3 SV=1	53.81526104	17	26	310000000	3.894	17
A0A0E0H6Y4	Glutamine synthetase OS=Oryza nivara OX=4536 PE=3 SV=1	39.3258427	16	25	240000000	12.689	16
A0A0E0IVK6	Uncharacterized protein OS=Oryza nivara OX=4536 PE=4 SV=1	23.04526749	15	203	20000000000	134.936	15
B8AW41	NAD(P)-bd_dom domain-containing protein OS=Oryza sativa subsp. indica OX=39946 GN=OsI_19308 PE=4 SV=1	40.4040404	15	18	93000000	2.268	15
A0A0E0HLA5	Geranylgeranyl reductase OS=Oryza nivara OX=4536 PE=3 SV=1	45.35637149	15	16	150000000	2.594	15
A3AV14	Glyceraldehyde-3-phosphate dehydrogenase OS=Oryza sativa subsp. japonica OX=39947 GN=OsJ_15252 PE=3 SV=1	51.94805195	14	25	430000000	5.918	14
A0A0E0HIB8	Uncharacterized protein OS=Oryza nivara OX=4536 PE=4 SV=1	52.26337449	13	18	560000000	7.799	13
P46265	Tubulin beta-5 chain OS=Oryza sativa subsp. japonica OX=39947 GN=TUBB5 PE=1 SV=1	37.36017897	13	16	16000000	3.217	13
A0A0E0ITA5	Tubulin beta chain OS=Oryza nivara OX=4536 PE=3 SV=1	37.52808989	13	16	26000000	3.217	13
A0A0E0HHS1	Protein kinase domain-containing protein OS=Oryza nivara OX=4536 PE=4 SV=1	29.05982906	13	14	71000000	1.39	13
A0A0P0XX30	Os10g0530500 protein OS=Oryza sativa subsp. japonica OX=39947 GN=Os10g0530500 PE=4 SV=1	72.8	13	51	2200000	128.155	13
A0A0E0FK45	Magnesium-protoporphyrin IX monomethyl ester (oxidative) cyclase OS=Oryza nivara OX=4536 PE=3 SV=1	31.67420814	13	15	76000000	2.039	13
A2YQT7	Glyceraldehyde-3-phosphate dehydrogenase, cytosolic OS=Oryza sativa subsp. indica OX=39946 GN=GAPC PE=2 SV=1	64.09495549	13	22	790000000	6.305	13
A2XVY3	Elongation factor G, chloroplastic OS=Oryza sativa subsp. indica OX=39946 GN=OsI_16800 PE=3 SV=1	23.51421189	13	16	87000000	1.121	13
B8A8L8	Uncharacterized protein OS=Oryza sativa subsp. indica OX=39946 GN=OsI_02088 PE=3 SV=1	45.34534535	12	14	170000000	3.329	12
A0A0E0IUD7	Uncharacterized protein OS=Oryza nivara OX=4536 PE=4 SV=1	35.74660633	12	16	82000000	2.914	12
Q7Y1F0	Serine hydroxymethyltransferase OS=Oryza sativa subsp. japonica OX=39947 GN=OSJNBa0057G07.17 PE=3 SV=1	37.70197487	12	14	49000000	1.448	12
Q0J128	Os09g0467200 protein OS=Oryza sativa subsp. japonica OX=39947 GN=Os09g0467200 PE=3 SV=1	48.87892377	12	33	1500000000	45.416	12
A0A0E0IF06	Glutamate-1-semialdehyde 2,1-aminomutase OS=Oryza nivara OX=4536 PE=3 SV=1	34.10041841	12	14	21000000	2.311	12
A0A0E0GWL1	Elongation factor Tu OS=Oryza nivara OX=4536 PE=3 SV=1	37.08609272	12	16	64000000	2.415	12
A2Y0Q8	CBM20 domain-containing protein OS=Oryza sativa subsp. indica OX=39946 GN=OsI_18583 PE=4 SV=1	21.76165803	12	12	48000000	0.823	12
A0A0E0IVE3	Uncharacterized protein OS=Oryza nivara OX=4536 PE=4 SV=1	14.15204678	11	17	2200000000	0.707	11
B8AF09	Glyceraldehyde-3-phosphate dehydrogenase OS=Oryza sativa subsp. indica OX=39946 GN=OsI_07948 PE=3 SV=1	43.25842697	11	18	98000000	3.365	11
B8B936	Glutamate-1-semialdehyde 2,1-aminomutase OS=Oryza sativa subsp. indica OX=39946 GN=OsI_30021 PE=3 SV=1	33.11688312	11	13	23000000	2.02	11
Q0JHF8	Fructose-1,6-bisphosphatase, cytosolic OS=Oryza sativa subsp. japonica OX=39947 GN=CFBP1 PE=1 SV=2	33.92330383	11	13	140000000	3.16	11
Q10QZ4	Elongation factor 1-alpha OS=Oryza sativa subsp. japonica OX=39947 GN=LOC_Os03g08060 PE=3 SV=1	43.65256125	11	24	680000000	2.857	11
E9KIQ1	Cytochrome f OS=Oryza sativa subsp. japonica OX=39947 GN=petA PE=3 SV=1	41.92546584	11	14	24000000	3.16	11
Q0JKY8	Carbonic anhydrase (Fragment) OS=Oryza sativa subsp. japonica OX=39947 GN=Os01g0639900 PE=3 SV=1	42.34875445	11	25	680000000	9	11
P0C520	ATP synthase subunit alpha, mitochondrial OS=Oryza sativa OX=4530 GN=ATPA PE=3 SV=1	27.11198428	11	16	260000000	1.656	11
A0A0E0IZ66	Aldehyde dehydrogenase OS=Oryza nivara OX=4536 PE=3 SV=1	27.32793522	11	13	63000000	2.311	11
A0A0E0IE06	Alanine--glyoxylate transaminase OS=Oryza nivara OX=4536 PE=3 SV=1	40.46511628	11	14	76000000	2.455	11
P0C539	Actin-2 OS=Oryza sativa subsp. indica OX=39946 GN=ACT2 PE=3 SV=1	45.88859416	11	17	12000000	3.642	11
A0A218KL39	Actin-1 OS=Oryza sativa OX=4530 PE=2 SV=1	45.88859416	11	20	96000000	4.109	11
A0A0E0HWH9	(S)-2-hydroxy-acid oxidase OS=Oryza nivara OX=4536 PE=3 SV=1	40.92140921	11	16	45000000	2.981	11
A0A0E0GUL1	(S)-2-hydroxy-acid oxidase OS=Oryza nivara OX=4536 PE=3 SV=1	19.7869102	11	14	160000000	1.712	11
B9F4B5	Uncharacterized protein OS=Oryza sativa subsp. japonica OX=39947 GN=OsJ_05923 PE=4 SV=1	26.51356994	10	11	14000000	1.102	10
A0A0E0IYB0	Uncharacterized protein OS=Oryza nivara OX=4536 PE=4 SV=1	24.70817121	10	10	16000000	0.931	10
A0A0E0IYI4	Uncharacterized protein OS=Oryza nivara OX=4536 PE=3 SV=1	40.15345269	10	19	120000000	3.365	10
A0A0E0FW49	Uncharacterized protein OS=Oryza nivara OX=4536 PE=3 SV=1	40.69148936	10	15	44000000	2.831	10

A0A0E0HUY0	Uncharacterized protein OS=Oryza nivara OX=4536 PE=3 SV=1	30.24282561	10	15	25000000	2.03	10
A2YIS2	Ribos_L4_asso_C domain-containing protein OS=Oryza sativa subsp. indica OX=39946 GN=OsI_25124 PE=3 SV=1	37.5308642	10	11	99000000	1.395	10
Q8GRU9	Phosphoribulokinase OS=Oryza sativa subsp. indica OX=39946 GN=OsI_08574 PE=2 SV=1	31.51364764	10	16	51000000	3.806	10
Q0J294	Os09g0367700 protein (Fragment) OS=Oryza sativa subsp. japonica OX=39947 GN=Os09g0367700 PE=4 SV=1	41.63090129	10	29	12000000000	12.594	10
Q94DL4	Os01g0964133 protein OS=Oryza sativa subsp. japonica OX=39947 GN=Os01g0964133 PE=2 SV=1	40.31830239	10	18	20000000	3.217	10
A0A0E0IFL1	NmrA domain-containing protein OS=Oryza nivara OX=4536 PE=4 SV=1	27.4611399	10	10	120000000	1.512	10
A0A0E0HVM5	Component of oligomeric Golgi complex 7 OS=Oryza nivara OX=4536 PE=3 SV=1	13.76744186	10	11	58000000	0.515	10
A2ZNT6	Uncharacterized protein OS=Oryza sativa subsp. japonica OX=39947 GN=OsJ_00219 PE=4 SV=1	35.47717842	9	13	43000000	1.807	9
B8BA11	Uncharacterized protein OS=Oryza sativa subsp. indica OX=39946 GN=OsI_28948 PE=4 SV=1	34.26573427	9	12	75000000	3.437	9
B8B3U4	Uncharacterized protein OS=Oryza sativa subsp. indica OX=39946 GN=OsI_23436 PE=4 SV=1	23.36244541	9	9	15000000	1.043	9
B8AMT7	Uncharacterized protein OS=Oryza sativa subsp. indica OX=39946 GN=OsI_14218 PE=3 SV=1	33.68700265	9	14		2.481	9
B8AKV8	Uncharacterized protein OS=Oryza sativa subsp. indica OX=39946 GN=OsI_12293 PE=3 SV=1	25.99009901	9	10	24000000	1.346	9
A2X0V4	Uncharacterized protein OS=Oryza sativa subsp. indica OX=39946 GN=OsI_05839 PE=3 SV=1	30.9178744	9	13	6600000	1.649	9
A0A0E0HK09	Uncharacterized protein OS=Oryza nivara OX=4536 PE=3 SV=1	22.29617304	9	12	120000000	1.069	9
B8B2X2	PSII_BNR domain-containing protein OS=Oryza sativa subsp. indica OX=39946 GN=OsI_24541 PE=4 SV=1	8.013468013	9	9	31000000	0.256	9
Q6H6C7	Phosphoglycerate kinase OS=Oryza sativa subsp. japonica OX=39947 GN=Os02g0169300 PE=2 SV=1	33.58208955	9	15	83000000	2.29	9
Q2QSR7	Os12g0420200 protein OS=Oryza sativa subsp. japonica OX=39947 GN=LOC_Os12g23180 PE=4 SV=2	27.12765957	9	14	70000000	2.675	9
A0A0E0H916	NAD(P)H dehydrogenase subunit H OS=Oryza nivara OX=4536 PE=3 SV=1	22.97794118	9	12	88000000	1.593	9
Q6WSC2	Glutathione S-transferase OS=Oryza sativa subsp. indica OX=39946 GN=gstu4 PE=2 SV=1	30.04291845	9	11	60000000	3.437	9
A2ZBX1	Fructose-bisphosphate aldolase OS=Oryza sativa subsp. indica OX=39946 GN=OsI_35277 PE=3 SV=1	37.62886598	9	11	370000000	1.424	9
A0A0E0HTC2	Fructose-bisphosphate aldolase OS=Oryza nivara OX=4536 PE=3 SV=1	23.67972743	9	9	54000000	0.951	9
A2WZT1	Ferredoxin--NADP reductase, leaf isozyme 2, chloroplastic OS=Oryza sativa subsp. indica OX=39946 GN=OsI_05475 PE=3 SV=1	34.69945355	9	9	25000000	1.096	9
A0A0E0IBG9	FAD_binding_3 domain-containing protein OS=Oryza nivara OX=4536 PE=3 SV=1	23.58078603	9	10	32000000	1.102	9
A0A0E0IS47	Epimerase domain-containing protein OS=Oryza nivara OX=4536 PE=4 SV=1	29.36507937	9	15	190000000	2.311	9
Q53N83	Chlorophyll a-b binding protein, chloroplastic OS=Oryza sativa subsp. japonica OX=39947 GN=LOC_Os11g13890 PE=2 SV=1	45.22968198	9	20		9	9
A0A0E0J063	Chlorophyll a-b binding protein, chloroplastic OS=Oryza nivara OX=4536 PE=3 SV=1	45.22968198	9	21	460000000	10.45	9
Q10MB2	30S ribosomal protein S1, chloroplast, putative, expressed OS=Oryza sativa subsp. japonica OX=39947 GN=LOC_Os03g20100 PE=4 SV=1	30.59701493	9	10	45000000	1.848	9
A2Y534	Uncharacterized protein OS=Oryza sativa subsp. indica OX=39946 GN=OsI_20107 PE=3 SV=1	32.71276596	8	11	10000000	1.371	8
A0A0E0H5N0	Uncharacterized protein OS=Oryza nivara OX=4536 PE=3 SV=1	28.41328413	8	12	15000000	2.793	8
A0A0E0J095	Tubulin alpha chain OS=Oryza nivara OX=4536 PE=3 SV=1	27.05099778	8	12	60000000	2.162	8
B8AI25	SET domain-containing protein OS=Oryza sativa subsp. indica OX=39946 GN=OsI_08832 PE=4 SV=1	23.25102881	8	9	20000000	1.291	8
Q2QTK0	Ribulose bisphosphate carboxylase small subunit OS=Oryza sativa subsp. japonica OX=39947 GN=LOC_Os12g19394 PE=3 SV=1	60.15625	8	10	620000000	4.623	8
A0A0E0IFI4	PCI domain-containing protein OS=Oryza nivara OX=4536 PE=3 SV=1	23.04526749	8	8	11000000	0.719	8
Q0DKP1	Methenyltetrahydrofolate cyclohydrolase (Fragment) OS=Oryza sativa subsp. japonica OX=39947 GN=Os05g0150800 PE=3 SV=1	39.51367781	8	9	51000000	1.462	8
A0A0E0GKD9	D-fructose-1,6-bisphosphate 1-phosphohydrolase OS=Oryza nivara OX=4536 PE=3 SV=1	25.83732057	8	13	71000000	3.833	8
B7ENR4	Aminomethyltransferase (Fragment) OS=Oryza sativa subsp. japonica OX=39947 PE=2 SV=1	28.11735941	8	8	55000000	0.778	8
A0A0H3UP39	3-ketoacyl-CoA thiolase-like protein OS=Oryza sativa subsp. japonica OX=39947 PE=2 SV=1	25	8	9	18000000	1.462	8
A3BQX7	Uncharacterized protein OS=Oryza sativa subsp. japonica OX=39947 GN=OsJ_26511 PE=3 SV=1	20	7	11	20000000	2.162	7
A2Z9K8	Uncharacterized protein OS=Oryza sativa subsp. indica OX=39946 GN=OsI_34418 PE=3 SV=1	23.82978723	7	9	34000000	2.652	7
A2YLT1	Uncharacterized protein OS=Oryza sativa subsp. indica OX=39946 GN=OsI_26177 PE=3 SV=1	21.22905028	7	9	200000000	1.404	7
A2WK17	Uncharacterized protein OS=Oryza sativa subsp. indica OX=39946 GN=OsI_00166 PE=4 SV=1	30.0330033	7	7	23000000	1.738	7
A0A0E0ISN2	Uncharacterized protein OS=Oryza nivara OX=4536 PE=4 SV=1	24.30703625	7	7	31000000	0.905	7
A0A0E0IWV4	Uncharacterized protein OS=Oryza nivara OX=4536 PE=3 SV=1	19.12751678	7	8	22000000	0.624	7
A0A0E0HKI3	Transketolase OS=Oryza nivara OX=4536 PE=3 SV=1	13.86271871	7	7	19000000	0.442	7
Q7XXS4	Thiamine thiazole synthase, chloroplastic OS=Oryza sativa subsp. japonica OX=39947 GN=THI1 PE=2 SV=1	30.42253521	7	8	37000000	1.081	7
A0A7G8PYR6	Ribulose bisphosphate carboxylase large chain (Fragment) OS=Oryza sativa OX=4530 GN=rbcL PE=3 SV=1	55.97484277	7	17	170000000	145.78	7
Q6AVA8	Pyruvate, phosphate dikinase 1, chloroplastic OS=Oryza sativa subsp. japonica OX=39947 GN=PPDK1 PE=1 SV=1	9.926082365	7	8	27000000	0.297	7
Q8W3D9	Protochlorophyllide reductase B, chloroplastic OS=Oryza sativa subsp. japonica OX=39947 GN=PORB PE=2 SV=1	27.6119403	7	10	69000000	1.346	7
Q0INX9	Os12g0277500 protein OS=Oryza sativa subsp. japonica OX=39947 GN=Os12g0277500 PE=3 SV=1	16.0899654	7	7	17000000	0.565	7
Q6K1X5	Os02g0608900 protein OS=Oryza sativa subsp. japonica OX=39947 GN=Os02g0608900 PE=2 SV=1	27.02702703	7	8	25000000	1.228	7
A0A0E0H2K9	KH type-2 domain-containing protein OS=Oryza nivara OX=4536 PE=3 SV=1	30.54662379	7	9	100000000	1.512	7
Q6AVT2	Glucose-1-phosphate adenylyltransferase large subunit 1, chloroplastic/amyloplastic OS=Oryza sativa subsp. japonica OX=39947 GN=AGPL1	19.17808219	7	7	10000000	0.528	7
B8AVM5	ATPase_AAA_core domain-containing protein OS=Oryza sativa subsp. indica OX=39946 GN=OsI_17750 PE=4 SV=1	20.61403509	7	8	24000000	0.812	7
A2XY71	AB hydrolase-1 domain-containing protein OS=Oryza sativa subsp. indica OX=39946 GN=OsI_17656 PE=4 SV=1	27.55102041	7	7	26000000	1.336	7

A0A0E0GG46	50S ribosomal protein L5, chloroplastic OS=Oryza nivara OX=4536 PE=3 SV=1	26.88172043	7	8	120000000	1.783	7
A0A0P0WU44	3-oxoacyl-[acyl-carrier-protein] synthase OS=Oryza sativa subsp. japonica OX=39947 GN=Os06g0196600 PE=3 SV=1	24.30107527	7	7	27000000	1.015	7
B9F4C0	Uncharacterized protein OS=Oryza sativa subsp. japonica OX=39947 GN=OsJ_05928 PE=4 SV=1	17.25490196	6	7	11000000	0.957	6
A2Z9K3	Uncharacterized protein OS=Oryza sativa subsp. indica OX=39946 GN=OsI_34413 PE=4 SV=1	28.13852814	6	11	3800000000	1.254	6
A2XMP7	Uncharacterized protein OS=Oryza sativa subsp. indica OX=39946 GN=OsI_13813 PE=3 SV=1	19.90407674	6	7	14000000	1.015	6
B8AGU8	Uncharacterized protein OS=Oryza sativa subsp. indica OX=39946 GN=OsI_05455 PE=3 SV=1	20.73170732	6	6	24000000	0.61	6
A0A0E0G8Z2	Uncharacterized protein OS=Oryza nivara OX=4536 PE=4 SV=1	37.61904762	6	6	19000000	1.371	6
A0A0E0I9K2	Uncharacterized protein OS=Oryza nivara OX=4536 PE=4 SV=1	14.73477407	6	6	17000000	0.61	6
A0A0E0J243	Uncharacterized protein OS=Oryza nivara OX=4536 PE=3 SV=1	12.30769231	6	6	7100000	0.453	6
A0A0E0GE88	Uncharacterized protein OS=Oryza nivara OX=4536 PE=3 SV=1	17.24845996	6	6	29000000	0.61	6
A0A0E0GCD6	Uncharacterized protein OS=Oryza nivara OX=4536 PE=3 SV=1	17.93478261	6	6	19000000	0.778	6
B8B8G2	Tubulin alpha chain OS=Oryza sativa subsp. indica OX=39946 GN=OsI_25395 PE=3 SV=1	18.26280624	6	8	19000000	1.154	6
Q84NN4	Thioredoxin-like protein CDSP32, chloroplastic OS=Oryza sativa subsp. japonica OX=39947 GN=CDSP32 PE=2 SV=1	20.26578073	6	6	18000000	1.069	6
A0A0R7VIP9	S-adenosylmethionine synthase OS=Oryza sativa subsp. japonica OX=39947 PE=2 SV=1	26.64974619	6	8	19000000	1.404	6
A0A0E0G9G3	Protein kinase domain-containing protein OS=Oryza nivara OX=4536 PE=4 SV=1	13.19796954	6	6	29000000	0.562	6
Q6ZG77	Probable diaminopimelate decarboxylase, chloroplastic OS=Oryza sativa subsp. japonica OX=39947 GN=LYSA PE=2 SV=1	16.12244898	6	7	9200000	0.682	6
A2WXD9	Photosystem II 22 kDa protein 1, chloroplastic OS=Oryza sativa subsp. indica OX=39946 GN=PSBS1 PE=1 SV=1	23.88059701	6	6	46000000	1.371	6
A2Z3M0	PCI domain-containing protein OS=Oryza sativa subsp. indica OX=39946 GN=OsI_32229 PE=3 SV=1	16.83778234	6	6	7500000	0.501	6
A2ZFY8	PAP_fibrillin domain-containing protein OS=Oryza sativa subsp. indica OX=39946 GN=OsI_36693 PE=4 SV=1	31.48148148	6	6	18000000	1.512	6
B9FL32	Os05g0512200 protein OS=Oryza sativa subsp. japonica OX=39947 GN=Os05g0512200 PE=4 SV=1	16.5158371	6	7	25000000	0.778	6
Q7X5X9	Os04g0444600 protein OS=Oryza sativa subsp. japonica OX=39947 GN=Os04g0444600 PE=2 SV=2	19.52941176	6	7	22000000	0.778	6
Q0JKR5	Isocitrate dehydrogenase [NADP] OS=Oryza sativa subsp. japonica OX=39947 GN=Os01g0654500 PE=3 SV=1	20.14563107	6	6	13000000	0.585	6
Q0IQW2	DnaK-type molecular chaperone hsp70-rice OS=Oryza sativa subsp. japonica OX=39947 GN=Os11g0703900 PE=3 SV=1	12.32665639	6	6	4100000	0.453	6
Q0JM17	DEAD-box ATP-dependent RNA helicase 56 OS=Oryza sativa subsp. japonica OX=39947 GN=AIP1 PE=1 SV=2	17.59259259	6	8	16000000	0.887	6
A0A0E0J938	Cysteine desulfurase OS=Oryza nivara OX=4536 PE=4 SV=1	14.07249467	6	6	21000000	0.501	6
A0A0E0GQ14	CobW C-terminal domain-containing protein OS=Oryza nivara OX=4536 PE=3 SV=1	17.7383592	6	6	29000000	0.823	6
Q6Z411	Chlorophyll a-b binding protein, chloroplastic OS=Oryza sativa subsp. japonica OX=39947 GN=RCABP69 PE=2 SV=1	30.68965517	6	8	88000000	2.162	6
A3A460	Chlorophyll a-b binding protein, chloroplastic OS=Oryza sativa subsp. japonica OX=39947 GN=OJ1524_D08.28-1 PE=3 SV=1	27.13754647	6	9	380000000	3.924	6
B8AME2	Catalase isozyme C OS=Oryza sativa subsp. indica OX=39946 GN=OsI_09857 PE=3 SV=1	18.49593496	6	6	29000000	0.638	6
A0A0E0HHW0	ATP synthase subunit beta OS=Oryza nivara OX=4536 PE=3 SV=1	8	6	7	19000000	0.251	6
A1YQJ8	ADP/ATP translocase OS=Oryza sativa subsp. japonica OX=39947 GN=Os02g0718900 PE=3 SV=1	14.13612565	6	9	60000000	1.081	6
O65103	23 kDa polypeptide of photosystem II OS=Oryza sativa OX=4530 PE=2 SV=2	31.88976378	6	8	33000000	1.783	6
A3ATE4	Uncharacterized protein OS=Oryza sativa subsp. japonica OX=39947 GN=OsJ_14634 PE=3 SV=1	8.131655373	5	5	11000000	0.155	5
B8AWN8	Uncharacterized protein OS=Oryza sativa subsp. indica OX=39946 GN=OsI_19498 PE=3 SV=1	9.514925373	5	5	24000000	0.354	5
A2XYG6	Uncharacterized protein OS=Oryza sativa subsp. indica OX=39946 GN=OsI_17742 PE=3 SV=1	12.77641278	5	5	18000000	0.688	5
A2XM46	Uncharacterized protein OS=Oryza sativa subsp. indica OX=39946 GN=OsI_13588 PE=3 SV=1	20.87378641	5	5	63000000	1.154	5
A0A0E0FYT1	Uncharacterized protein OS=Oryza nivara OX=4536 PE=4 SV=1	12.58741259	5	48	12000000	0.778	5
A0A0E0FJF4	Uncharacterized protein OS=Oryza nivara OX=4536 PE=4 SV=1	19.22005571	5	6	44000000	0.995	5
A0A0E0FZU7	Uncharacterized protein OS=Oryza nivara OX=4536 PE=3 SV=1	8.351893096	5	10	26000000	0.567	5
A0A0E0GY30	Uncharacterized protein OS=Oryza nivara OX=4536 PE=3 SV=1	15.30612245	5	5	47000000	0.585	5
A0A0E0IYF9	Uncharacterized protein OS=Oryza nivara OX=4536 PE=3 SV=1	6.060606061	5	5	8900000	0.112	5
A0A0E0IMV7	Uncharacterized protein OS=Oryza nivara OX=4536 PE=3 SV=1	20.39473684	5	5	12000000	0.896	5
A0A0E0IVN8	UDP-arabinopyranose mutase OS=Oryza nivara OX=4536 PE=3 SV=1	17.85714286	5	5	9600000	0.616	5
Q0D584	Tubulin alpha chain OS=Oryza sativa subsp. japonica OX=39947 GN=Os07g0574800 PE=3 SV=1	16.44444444	5	8		1.31	5
A0A0E0GES8	S-(hydroxymethyl)glutathione dehydrogenase OS=Oryza nivara OX=4536 PE=3 SV=1	14.63963964	5	5	22000000	0.468	5
A2ZCZ7	Rieske domain-containing protein OS=Oryza sativa subsp. indica OX=39946 GN=OsI_35659 PE=4 SV=1	26.35379061	5	5	40000000	0.896	5
Q259K4	Pyruvate kinase OS=Oryza sativa OX=4530 GN=H0402C08.9 PE=3 SV=1	12.32876712	5	5	12000000	0.417	5
B8ABM9	Pyr_redox_2 domain-containing protein OS=Oryza sativa subsp. indica OX=39946 GN=OsI_04297 PE=4 SV=1	10.46277666	5	5	36000000	0.433	5
Q9SNL7	Putative magnesium-protoporphyrin IX methyltransferase OS=Oryza sativa subsp. japonica OX=39947 GN=134P10.4 PE=4 SV=1	15.64417178	5	5	19000000	0.688	5
B8BCJ0	Protein disulfide-isomerase OS=Oryza sativa subsp. indica OX=39946 GN=OsI_31602 PE=3 SV=1	13.84615385	5	5	12000000	0.532	5
A0A0E0J851	PKS_ER domain-containing protein OS=Oryza nivara OX=4536 PE=4 SV=1	13.39285714	5	5	27000000	0.688	5
A0A1W5HL37	Photosystem II CP43 reaction center protein OS=Oryza nivara OX=4536 GN=psbC PE=3 SV=1	13.10782241	5	8	130000000	2.162	5
A0A0E0FUH1	Phosphoglycerate kinase OS=Oryza nivara OX=4536 PE=3 SV=1	15.14476615	5	14	42000000	1.326	5
A0A0E0IEF7	Phenylalanyl-tRNA synthetase OS=Oryza nivara OX=4536 PE=3 SV=1	13.61607143	5	6	8200000	0.562	5

A0A0E0JBN1	Peptidase A1 domain-containing protein OS=Oryza nivara OX=4536 PE=3 SV=1	14.46540881	5	7	45000000	0.823	5
A0A5S6R9E3	Os09g0327100 protein OS=Oryza sativa subsp. japonica OX=39947 GN=Os09g0327100 PE=4 SV=1	24.53703704	5	5	3100000	1.054	5
Q0DAL0	Os06g0646500 protein (Fragment) OS=Oryza sativa subsp. japonica OX=39947 GN=Os06g0646500 PE=3 SV=1	19.18819188	5	6	15000000	0.931	5
Q0DHJ8	Os05g0461300 protein OS=Oryza sativa subsp. japonica OX=39947 GN=Os05g0461300 PE=4 SV=1	28.7037037	5	5	17000000	0.896	5
B9FJH8	Os05g0460000 protein OS=Oryza sativa subsp. japonica OX=39947 GN=Os05g0460000 PE=3 SV=1	10.0619195	5	5	4300000	0.365	5
Q0DZW0	Os02g0595500 protein OS=Oryza sativa subsp. japonica OX=39947 GN=Os02g0595500 PE=3 SV=1	21.42857143	5	6	18000000	0.896	5
Q0E446	Os02g0137200 protein (Fragment) OS=Oryza sativa subsp. japonica OX=39947 GN=Os02g0137200 PE=3 SV=1	23.35766423	5	5	48000000	0.968	5
A2ZWC8	Os01g0667600 protein OS=Oryza sativa subsp. japonica OX=39947 GN=Os01g0667600 PE=4 SV=1	23.66071429	5	5	19000000	1.054	5
A0A0E0GUE4	Malate dehydrogenase OS=Oryza nivara OX=4536 PE=3 SV=1	16.84665227	5	7	40000000	0.562	5
A0A0E0FK09	Isocitrate dehydrogenase [NAD] subunit, mitochondrial OS=Oryza nivara OX=4536 PE=3 SV=1	12.31884058	5	7	43000000	0.738	5
A0A0E0I8K6	Glutamyl-tRNA(Gln) amidotransferase subunit A, chloroplastic/mitochondrial OS=Oryza nivara OX=4536 GN=GATA PE=3 SV=1	13.79310345	5	5	22000000	0.45	5
A2XMW9	ENT domain-containing protein OS=Oryza sativa subsp. indica OX=39946 GN=OsI_13894 PE=3 SV=1	9.302325581	5	6	16000000	0.259	5
A2XYX9	Dihydroorotate dehydrogenase (quinone), mitochondrial OS=Oryza sativa subsp. indica OX=39946 GN=OsI_17913 PE=3 SV=1	11.94029851	5	5	8200000	0.557	5
A0A0E0IN07	DHQ_synthase domain-containing protein OS=Oryza nivara OX=4536 PE=3 SV=1	12.64108352	5	5	12000000	0.532	5
A0A0E0IHY0	Cysteine desulfurase OS=Oryza nivara OX=4536 PE=3 SV=1	12.21995927	5	5	7900000	0.487	5
A0A7S7YAC0	Clp protease proteolytic subunit OS=Oryza sativa temperate japonica subgroup OX=1736657 GN=clpP PE=4 SV=1	25.92592593	5	6	44000000	3.642	5
B9EXM2	Carbamoyl-phosphate synthase large chain, chloroplastic OS=Oryza sativa subsp. japonica OX=39947 GN=CARB PE=2 SV=1	6.399317406	5	5	12000000	0.179	5
A0A0E0FZW8	40S ribosomal protein S4 OS=Oryza nivara OX=4536 PE=3 SV=1	21.50943396	5	5	12000000	0.73	5
A0A0E0J9J1	40S ribosomal protein S3a OS=Oryza nivara OX=4536 PE=3 SV=1	6.093189964	5	6	25000000	0.318	5
A2XTX6	3-oxoacyl-[acyl-carrier-protein] synthase OS=Oryza sativa subsp. indica OX=39946 GN=OsI_16057 PE=3 SV=1	9.090909091	5	5	17000000	0.688	5
A2Z9J0	Uncharacterized protein OS=Oryza sativa subsp. indica OX=39946 GN=OsI_34389 PE=3 SV=1	18.03278689	4	4	32000000	0.54	4
A2Y7K1	Uncharacterized protein OS=Oryza sativa subsp. indica OX=39946 GN=OsI_21018 PE=3 SV=1	9.929078014	4	4	3800000	0.346	4
B8AVV2	Uncharacterized protein OS=Oryza sativa subsp. indica OX=39946 GN=OsI_16423 PE=4 SV=1	18.35616438	4	4	14000000	0.624	4
B8A7L7	Uncharacterized protein OS=Oryza sativa subsp. indica OX=39946 GN=OsI_03288 PE=4 SV=1	13.15789474	4	4	12000000	0.445	4
A0A0E0IVE4	Uncharacterized protein OS=Oryza nivara OX=4536 PE=4 SV=1	18.18181818	4	4	15000000	0.848	4
A0A0E0IGE4	Uncharacterized protein OS=Oryza nivara OX=4536 PE=4 SV=1	7.601351351	4	4	3300000	0.301	4
A0A0E0GWA8	Uncharacterized protein OS=Oryza nivara OX=4536 PE=4 SV=1	21.52466368	4	4	5000000	0.778	4
A0A0E0HIB5	Uncharacterized protein OS=Oryza nivara OX=4536 PE=4 SV=1	19.23076923	4	4	140000000	0.719	4
A0A0E0FS05	Uncharacterized protein OS=Oryza nivara OX=4536 PE=3 SV=1	10.12658228	4	4	9100000	0.334	4
A0A0E0FW90	Uncharacterized protein OS=Oryza nivara OX=4536 PE=3 SV=1	4	4	4	5000000	0.172	4
A0A218KL52	Triosephosphate isomerase OS=Oryza sativa OX=4530 PE=2 SV=1	20.55335968	4	4	15000000	0.931	4
Q6WSC3	Tau class GST protein 4 OS=Oryza sativa subsp. indica OX=39946 GN=OsI_34420 PE=2 SV=1	20.08368201	4	4	13000000	0.719	4
B9F0A5	Quinol--cytochrome-c reductase OS=Oryza sativa subsp. japonica OX=39947 GN=OsJ_06940 PE=3 SV=1	14.66346154	4	4	39000000	0.585	4
A3B9A1	Putative chaperonin 21 OS=Oryza sativa subsp. japonica OX=39947 GN=P0528E04.36-1 PE=3 SV=1	28.17460317	4	4	19000000	0.668	4
B8AA22	Protein-serine/threonine phosphatase OS=Oryza sativa subsp. indica OX=39946 GN=OsI_02395 PE=3 SV=1	11.79487179	4	5	21000000	0.73	4
Q10SY1	Protein tyrosine phosphatase, putative, expressed OS=Oryza sativa subsp. japonica OX=39947 GN=LOC_Os03g01750 PE=4 SV=1	13.58695652	4	4	14000000	0.492	4
B9FVN3	Prolyl-tRNA synthetase OS=Oryza sativa subsp. japonica OX=39947 GN=OsJ_23204 PE=3 SV=1	10.64220183	4	4	17000000	0.311	4
A2XMN2	Probable glutathione S-transferase GSTU1 OS=Oryza sativa subsp. indica OX=39946 GN=GSTU1 PE=1 SV=1	18.18181818	4	10	3900000000	0.833	4
E9KIM8	Photosystem II D2 protein OS=Oryza sativa subsp. japonica OX=39947 GN=psbD PE=3 SV=1	17.54874652	4	5	160000000	1.31	4
A0A0E0H2J6	PDZ domain-containing protein OS=Oryza nivara OX=4536 PE=3 SV=1	12.25961538	4	4	7200000	0.624	4
A0A0E0GWL3	PCI domain-containing protein OS=Oryza nivara OX=4536 PE=3 SV=1	8.66807611	4	5	13000000	0.417	4
P83646	Oxygen-evolving enhancer protein 3, chloroplastic OS=Oryza sativa subsp. indica OX=39946 GN=OsI_025465 PE=1 SV=2	29.03225806	4	7	26000000	2.455	4
A0A0P0Y3G3	Os11g0544800 protein (Fragment) OS=Oryza sativa subsp. japonica OX=39947 GN=Os11g0544800 PE=3 SV=1	10.3074141	4	4	6700000	0.274	4
A0A0N7KS21	Os10g0509200 protein OS=Oryza sativa subsp. japonica OX=39947 GN=Os10g0509200 PE=4 SV=1	16.84210526	4	5	45000000	0.624	4
Q6YUA7	Os08g0464000 protein OS=Oryza sativa subsp. japonica OX=39947 GN=Os08g0464000 PE=2 SV=1	16.01123596	4	4	19000000	0.425	4
A0A0P0X3G1	Os07g0195100 protein (Fragment) OS=Oryza sativa subsp. japonica OX=39947 GN=Os07g0195100 PE=4 SV=1	22.34432234	4	4	20000000	0.624	4
Q6K919	Os02g0596000 protein OS=Oryza sativa subsp. japonica OX=39947 GN=Os02g0596000 PE=4 SV=1	17.12328767	4	4	20000000	0.624	4
A0A0E0ITA8	Obg-like ATPase 1 OS=Oryza nivara OX=4536 PE=3 SV=1	9.836065574	4	5	9500000	0.407	4
B8AMJ8	N-acetyl-glutamate semialdehyde dehydrogenase OS=Oryza sativa subsp. indica OX=39946 GN=OsI_12624 PE=3 SV=1	11.08433735	4	4	4700000	0.389	4
J3SBY5	Mannose-1-phosphate guanyltransferase 3 OS=Oryza sativa subsp. indica OX=39946 PE=2 SV=1	14.95844875	4	4	12000000	0.624	4
A0A0E0HFK5	GTP cyclohydrolase II OS=Oryza nivara OX=4536 PE=3 SV=1	8.768656716	4	4	6100000	0.374	4
A0A0E0FT61	Glyco_hydro_18 domain-containing protein OS=Oryza nivara OX=4536 PE=4 SV=1	16.16161616	4	4	31000000	0.848	4
A0A0E0H7V8	Glucose-1-phosphate adenylyltransferase OS=Oryza nivara OX=4536 PE=3 SV=1	12.02090592	4	4	13000000	0.259	4
A0A1L2JKJ5	Fructose-bisphosphate aldolase OS=Oryza sativa OX=4530 PE=2 SV=1	16.20111732	4	4	19000000	0.551	4

A0A0E0HJQ3	Ferredoxin--NADP reductase, chloroplastic OS=Oryza nivara OX=4536 PE=3 SV=1	8.208955224	4	4	22000000	0.389	4
A2ZMY2	Cysteine synthase OS=Oryza sativa subsp. indica OX=39946 GN=OsI_39190 PE=3 SV=1	14.46078431	4	4	15000000	0.407	4
A0A0E0FZP7	Cysteine synthase OS=Oryza nivara OX=4536 PE=3 SV=1	13.93643032	4	4	11000000	0.492	4
A0A0E0IXJ3	CTP:phosphoethanolamine cytidylyltransferase OS=Oryza nivara OX=4536 PE=3 SV=1	14.97695853	4	5	11000000	0.557	4
Q10HD0	Chlorophyll a-b binding protein, chloroplastic OS=Oryza sativa subsp. japonica OX=39947 GN=RCABP89 PE=2 SV=1	15.96958175	4	7	130000000	1.154	4
Q5NBF5	ATP-dependent Clp protease proteolytic subunit OS=Oryza sativa subsp. japonica OX=39947 GN=OsJ_01248 PE=3 SV=1	16.33986928	4	4	21000000	0.668	4
A0A0E0HJ81	ATP-dependent Clp protease proteolytic subunit OS=Oryza nivara OX=4536 PE=3 SV=1	14.76683938	4	4	5900000	0.492	4
A2ZH35	ATPase_AAA_core domain-containing protein OS=Oryza sativa subsp. indica OX=39946 GN=OsI_37097 PE=4 SV=1	15.27777778	4	24	380000000	1.424	4
B8BMJ6	ATP citrate synthase OS=Oryza sativa subsp. indica OX=39946 GN=OsI_38756 PE=3 SV=1	10.50328228	4	4	7100000	0.52	4
A0A0E0GE32	Aspartate aminotransferase OS=Oryza nivara OX=4536 PE=3 SV=1	10.69868996	4	4	4500000	0.346	4
A2X6N1	60S ribosomal protein L6 OS=Oryza sativa subsp. indica OX=39946 GN=OsI_07870 PE=3 SV=1	15.52511416	4	5	12000000	0.896	4
A0A0K0LRP3	30S ribosomal protein S3, chloroplastic OS=Oryza nivara OX=4536 GN=rps3 PE=3 SV=1	17.9916318	4	5	25000000	1.154	4
B8BLZ1	2-isopropylmalate synthase OS=Oryza sativa subsp. indica OX=39946 GN=OsI_37410 PE=3 SV=1	8.661417323	4	4	8300000	0.252	4
A0A0E0FR17	Zeta-carotene desaturase OS=Oryza nivara OX=4536 PE=3 SV=1	5.047748977	3	3	9700000	0.17	3
A0A0E0H1H7	Zeaxanthin epoxidase, chloroplastic OS=Oryza nivara OX=4536 PE=4 SV=1	5.007587253	3	3	7200000	0.189	3
B9FMC0	Uncharacterized protein OS=Oryza sativa subsp. japonica OX=39947 GN=OsJ_17039 PE=3 SV=1	7.984031936	3	3	73000000	0.318	3
A2Z9J5	Uncharacterized protein OS=Oryza sativa subsp. indica OX=39946 GN=OsI_34395 PE=4 SV=1	15.56603774	3	3	15000000	0.638	3
A2YKQ6	Uncharacterized protein OS=Oryza sativa subsp. indica OX=39946 GN=OsI_25804 PE=3 SV=1	41.89189189	3	3	3700000	1.371	3
B8ASM5	Uncharacterized protein OS=Oryza sativa subsp. indica OX=39946 GN=OsI_16848 PE=4 SV=1	3.692307692	3	4	32000000	0.112	3
A0A0E0GKP6	Uncharacterized protein OS=Oryza nivara OX=4536 PE=4 SV=1	15.69230769	3	3	13000000	0.413	3
A0A0E0GR18	Uncharacterized protein OS=Oryza nivara OX=4536 PE=3 SV=1	8.048289738	3	4	4100000	0.407	3
A0A0E0HZY4	Uncharacterized protein OS=Oryza nivara OX=4536 PE=3 SV=1	12.06896552	3	3	13000000	0.874	3
A0A0E0H9D0	Uncharacterized protein OS=Oryza nivara OX=4536 PE=3 SV=1	6.91588785	3	3	4600000	0.233	3
A0A0B4U1W3	UDP-glucose 6-dehydrogenase (Fragment) OS=Oryza sativa OX=4530 GN=LOC_Os03g55070.1 PE=2 SV=1	7.916666667	3	3	16000000	0.212	3
A0A0P0X6U8	Ubiquitin OS=Oryza sativa subsp. japonica OX=39947 GN=Os07g0489500 PE=3 SV=1	14.96062992	3	5	32000000	0.585	3
A2XPF9	Thioredoxin-like_fold domain-containing protein OS=Oryza sativa subsp. indica OX=39946 GN=OsI_14471 PE=4 SV=1	18.14159292	3	3	11000000	0.585	3
A0A0E0G7U2	Thioredoxin-dependent peroxiredoxin OS=Oryza nivara OX=4536 PE=4 SV=1	18.25095057	3	3	29000000	0.438	3
B9F2N9	Sucrose-phosphatase OS=Oryza sativa subsp. japonica OX=39947 GN=OsJ_05345 PE=3 SV=1	6.776180698	3	3	11000000	0.292	3
A2YNN3	Str_synth domain-containing protein OS=Oryza sativa subsp. indica OX=39946 GN=OsI_26852 PE=3 SV=1	12.23404255	3	3	24000000	0.438	3
B9F6V9	S5 DRBM domain-containing protein OS=Oryza sativa subsp. japonica OX=39947 GN=OsJ_13042 PE=3 SV=1	13.38028169	3	3	20000000	0.438	3
B8APA6	Ribulose-phosphate 3-epimerase OS=Oryza sativa subsp. indica OX=39946 GN=OsI_10180 PE=3 SV=1	22.62773723	3	5	49000000	1.276	3
A0A0E0ICN1	Ribosomal_L2_C domain-containing protein OS=Oryza nivara OX=4536 PE=3 SV=1	21.83908046	3	4	3200000	0.668	3
B8A7P8	Ribosomal_L18_c domain-containing protein OS=Oryza sativa subsp. indica OX=39946 GN=OsI_04778 PE=3 SV=1	17.10526316	3	5	13000000	0.73	3
A0A0E0HXB4	Ribose-5-phosphate isomerase OS=Oryza nivara OX=4536 PE=3 SV=1	5.786618445	3	4	21000000	0.346	3
B8AI07	Rhodanese domain-containing protein OS=Oryza sativa subsp. indica OX=39946 GN=OsI_08794 PE=4 SV=1	4.825737265	3	3	5800000	0.166	3
A0A0E0J535	RF_PROK_I domain-containing protein OS=Oryza nivara OX=4536 PE=3 SV=1	12.74038462	3	3	13000000	0.304	3
Q84SS7	Putative ribosomal protein S5 OS=Oryza sativa subsp. japonica OX=39947 GN=OSJNBb0047D08.4 PE=3 SV=1	9.785932722	3	3	16000000	0.438	3
A0A0P0VFV0	Putative lycopene beta-cyclase OS=Oryza sativa subsp. japonica OX=39947 GN=OSJNBb0031B09.22 PE=3 SV=1	8.588957055	3	3	5600000	0.269	3
A0A0P0X2Y5	Putative glutathione S-transferase GST27 OS=Oryza sativa subsp. japonica OX=39947 GN=OSJNBa0050F10.6 PE=4 SV=1	15.74468085	3	3	21000000	0.468	3
A0A0E0IT94	PSI-F OS=Oryza nivara OX=4536 PE=3 SV=1	19.91525424	3	3	27000000	0.701	3
A2XAD8	Protein kinase domain-containing protein OS=Oryza sativa subsp. indica OX=39946 GN=OsI_09218 PE=3 SV=1	10.29810298	3	3	5500000	0.413	3
Q6ZJJ1	Probable L-ascorbate peroxidase 4, peroxisomal OS=Oryza sativa subsp. japonica OX=39947 GN=APX4 PE=2 SV=1	14.43298969	3	3	5000000	0.369	3
A0A0E0IM07	PKS_ER domain-containing protein OS=Oryza nivara OX=4536 PE=4 SV=1	12.35955056	3	3	6100000	0.369	3
A0A0E0IB67	PKS_ER domain-containing protein OS=Oryza nivara OX=4536 PE=3 SV=1	9.929078014	3	3	6900000	0.28	3
J7EYL8	Photosystem II protein D1 OS=Oryza sativa subsp. indica OX=39946 GN=psbA PE=3 SV=1	11.898017	3	4	160000000	0.778	3
B8B7H0	Peptidylprolyl isomerase OS=Oryza sativa subsp. indica OX=39946 GN=OsI_26503 PE=4 SV=1	8.101851852	3	3	9500000	0.318	3
A0A0E0GSK4	Peptidylprolyl isomerase OS=Oryza nivara OX=4536 PE=4 SV=1	16.26984127	3	3	10000000	0.995	3
A0A0E0HIM5	PDZ domain-containing protein OS=Oryza nivara OX=4536 PE=3 SV=1	13.27231121	3	3	4800000	0.222	3
Q75IQ4	Outer envelope pore protein 24, chloroplastic OS=Oryza sativa subsp. japonica OX=39947 GN=OEP24 PE=2 SV=1	18.75	3	3	11000000	0.585	3
Q0JCD0	Os04g0479200 protein (Fragment) OS=Oryza sativa subsp. japonica OX=39947 GN=Os04g0479200 PE=3 SV=1	10.60240964	3	4		0.413	3
A0A0E0FYU2	OMPdecase OS=Oryza nivara OX=4536 PE=3 SV=1	3.215434084	3	3	12000000	0.166	3
Q6Z1J6	Obg-like ATPase 1 OS=Oryza sativa subsp. japonica OX=39947 GN=YCHF1 PE=1 SV=1	8.629441624	3	3	2800000	0.304	3
B9FF00	NAD(P)-bd_dom domain-containing protein OS=Oryza sativa subsp. japonica OX=39947 GN=OsJ_14672 PE=4 SV=1	11.98830409	3	4	7500000	0.668	3
Q53PA7	Mitochondrial carrier protein, expressed OS=Oryza sativa subsp. japonica OX=39947 GN=LOC_Os11g24450 PE=2 SV=1	13.26860841	3	3	35000000	0.438	3

A0A0E0GPP9	Mg-protoporphyrin IX chelatase OS=Oryza nivara OX=4536 PE=3 SV=1	9.879518072	3	3	4400000	0.304	3
A2YMD1	Isopentenyl-diphosphate Delta-isomerase OS=Oryza sativa subsp. indica OX=39946 GN=OsI_26387 PE=3 SV=1	6.648199446	3	3	7200000	0.148	3
A3ABH0	Histidine--tRNA ligase OS=Oryza sativa subsp. japonica OX=39947 GN=Os02g0754700 PE=3 SV=1	9.034907598	3	3	9200000	0.292	3
Q259H2	H0103C06.7 protein OS=Oryza sativa OX=4530 GN=H0103C06.7 PE=4 SV=1	8.865979381	3	3	16000000	0.259	3
A0A0E0G4W1	Glyoxalase I OS=Oryza nivara OX=4536 PE=3 SV=1	10	3	3	4400000	0.269	3
A0A0E0HP64	Glycosyltransferase OS=Oryza nivara OX=4536 PE=3 SV=1	8.074534161	3	4	10000000	0.407	3
A0A0E0G300	Glycosyltransferase OS=Oryza nivara OX=4536 PE=3 SV=1	9.224318658	3	3	8400000	0.501	3
Q0E3J3	Glyceraldehyde-3-phosphate dehydrogenase OS=Oryza sativa subsp. japonica OX=39947 GN=Os02g0171100 PE=3 SV=1	11.92214112	3	3	7000000	0.269	3
A2X8S4	Glutamine amidotransferase type-1 domain-containing protein OS=Oryza sativa subsp. indica OX=39946 GN=OsI_08636 PE=3 SV=1	9.417040359	3	3	10000000	0.389	3
Q8LLP2	Glutamate decarboxylase OS=Oryza sativa subsp. japonica OX=39947 GN=OSJNBa0031O09.06 PE=3 SV=1	6.627680312	3	3	6500000	0.28	3
A0A0E0ICZ8	Germin-like protein OS=Oryza nivara OX=4536 PE=3 SV=1	15.96244131	3	8	91000000	5.813	3
A0A0E0HE79	Fructose-bisphosphate aldolase OS=Oryza nivara OX=4536 PE=3 SV=1	12.5698324	3	3		0.369	3
A0A0E0HY40	Epimerase domain-containing protein OS=Oryza nivara OX=4536 PE=4 SV=1	13.74045802	3	4	22000000	0.468	3
A0A542V9L0	Elongation factor Tu OS=Pseudomonas sp. SLBN-26 OX=2768443 GN=tuf PE=3 SV=1	9.068010076	3	3	16000000	0.304	3
A0A0P0V236	Dihydrolipoyl dehydrogenase OS=Oryza sativa subsp. japonica OX=39947 GN=Os01g0328700 PE=3 SV=1	9.343936382	3	3	4800000	0.25	3
A0A0E0H6Q4	D-3-phosphoglycerate dehydrogenase OS=Oryza nivara OX=4536 PE=3 SV=1	7.830342577	3	3	5200000	0.233	3
Q7XLG4	Cytochrome b-c1 complex subunit Rieske, mitochondrial OS=Oryza sativa subsp. japonica OX=39947 GN=Os04g0398500 PE=2 SV=2	12.94964029	3	3	16000000	0.501	3
B9G3G3	Cinnamyl-alcohol dehydrogenase OS=Oryza sativa subsp. japonica OX=39947 GN=OsJ_29281 PE=3 SV=1	15.75342466	3	4	9300000	0.492	3
A2YMN1	Chlorophyll a-b binding protein, chloroplastic OS=Oryza sativa subsp. indica OX=39946 GN=OsI_26482 PE=3 SV=1	20.30075188	3	4	150000000	1.031	3
A2WUJ5	Chlorophyll a-b binding protein, chloroplastic OS=Oryza sativa subsp. indica OX=39946 GN=OsI_03546 PE=3 SV=1	16.98113208	3	9	25000000	1.154	3
A0A0E0INE9	C2 NT-type domain-containing protein OS=Oryza nivara OX=4536 PE=4 SV=1	5.322763307	3	3	14000000	0.124	3
Q0DRY8	ATP-dependent Clp protease proteolytic subunit OS=Oryza sativa subsp. japonica OX=39947 GN=Os03g0344900 PE=3 SV=1	12.03438395	3	3	17000000	0.389	3
A2X7K2	ATP-dependent Clp protease proteolytic subunit OS=Oryza sativa subsp. indica OX=39946 GN=OsI_08189 PE=3 SV=1	21.69811321	3	5	150000000	1.424	3
A0A0E0J5G3	ATP citrate synthase OS=Oryza nivara OX=4536 GN=BBa0102J12.23-1 PE=3 SV=1	8.274231678	3	3	4000000	0.389	3
Q8RUM9	Amidophosphoribosyltransferase OS=Oryza sativa subsp. japonica OX=39947 GN=OSJNBb0008G24.26 PE=3 SV=1	7.259528131	3	5	6600000	0.346	3
B8BKT7	Alpha-mannosidase OS=Oryza sativa subsp. indica OX=39946 GN=OsI_36309 PE=3 SV=1	3.522504892	3	3	6100000	0.126	3
A3C1M3	Aldo_ket_red domain-containing protein OS=Oryza sativa subsp. japonica OX=39947 GN=OsJ_30388 PE=4 SV=1	10.33755274	3	4	19000000	0.346	3
A0A0E0HC71	ADP/ATP translocase OS=Oryza nivara OX=4536 PE=3 SV=1	7.623318386	3	5	3200000	0.374	3
A0A0N7KFQ3	Adenosine kinase (Fragment) OS=Oryza sativa subsp. japonica OX=39947 GN=Os02g0625500 PE=3 SV=1	10.37234043	3	3	8600000	0.35	3
Q0DFA4	Acetyltransferase component of pyruvate dehydrogenase complex OS=Oryza sativa subsp. japonica OX=39947 GN=P0644B06.24-2 PE=3 SV=	8.181818182	3	3	3100000	0.233	3
A0A0E0FZW3	Acetyltransferase component of pyruvate dehydrogenase complex OS=Oryza nivara OX=4536 PE=3 SV=1	7.846715328	3	3	4300000	0.25	3
A0A0E0H8X8	Acetylornithine transaminase OS=Oryza nivara OX=4536 PE=3 SV=1	9.406952965	3	3	6400000	0.292	3
B9FIP1	AB hydrolase-1 domain-containing protein OS=Oryza sativa subsp. japonica OX=39947 GN=OsJ_18574 PE=4 SV=1	10	3	3	15000000	0.413	3
B8AM18	AAA domain-containing protein OS=Oryza sativa subsp. indica OX=39946 GN=OsI_11173 PE=3 SV=1	6.739130435	3	3	9500000	0.166	3
A0A0E0HVR0	AAA domain-containing protein OS=Oryza nivara OX=4536 PE=3 SV=1	5.393586006	3	4	8200000	0.359	3
Q7F4T2	50S ribosomal protein L14, chloroplastic OS=Oryza sativa subsp. japonica OX=39947 GN=rpl14 PE=3 SV=1	30.08130081	3	3	12000000	1.154	3
A0A5S6RDN1	40S ribosomal protein S8 OS=Oryza sativa subsp. japonica OX=39947 GN=Os04g0349500 PE=3 SV=1	19.00452489	3	3	5500000	0.778	3
B9F279	3-isopropylmalate dehydratase OS=Oryza sativa subsp. japonica OX=39947 GN=Os02g0125100 PE=4 SV=1	7.782101167	3	4	6400000	0.346	3
Q2QM70	37 kDa inner envelope membrane protein, chloroplast, putative, expressed OS=Oryza sativa subsp. japonica OX=39947 GN=LOC_Os12g4209	11.68091168	3	3	16000000	0.438	3
B8ANT6	30S ribosomal protein S4, chloroplastic OS=Oryza sativa subsp. indica OX=39946 GN=OsI_10067 PE=3 SV=1	12.67123288	3	4	8300000	0.778	3
E9KIN4	30S ribosomal protein S2, chloroplastic OS=Oryza sativa subsp. japonica OX=39947 GN=rps2 PE=3 SV=1	12.85140562	3	3	29000000	0.501	3
A2WJR2	1-deoxy-D-xylulose-5-phosphate reductoisomerase OS=Oryza sativa subsp. indica OX=39946 GN=OsI_00059 PE=3 SV=1	6.976744186	3	3	8900000	0.269	3
A0A0E0HIU0	Very-long-chain 3-oxoacyl-CoA synthase OS=Oryza nivara OX=4536 PE=3 SV=1	3.149606299	2	3	19000000	0.199	2
A0A0E0G252	V-ATPase 69 kDa subunit OS=Oryza nivara OX=4536 PE=3 SV=1	5.152979066	2	2	9000000	0.105	2
A3QQQ3	UTP--glucose-1-phosphate uridylyltransferase OS=Oryza sativa subsp. indica OX=39946 GN=UGP PE=2 SV=1	5.117270789	2	2	3400000	0.155	2
Q84Q70	Uncharacterized protein OSJNBa0071M09.9 OS=Oryza sativa subsp. japonica OX=39947 GN=OSJNBa0071M09.9 PE=3 SV=1	7.395498392	2	2	8600000	0.259	2
B9G1W3	Uncharacterized protein OS=Oryza sativa subsp. japonica OX=39947 GN=OsJ_28011 PE=4 SV=1	6.586826347	2	2	27000000	0.389	2
B9FWP5	Uncharacterized protein OS=Oryza sativa subsp. japonica OX=39947 GN=OsJ_23874 PE=4 SV=1	6.946983547	2	2	4500000	0.15	2
B9FS66	Uncharacterized protein OS=Oryza sativa subsp. japonica OX=39947 GN=OsJ_20585 PE=4 SV=1	2.166476625	2	2	6000000	0.091	2
B9FF36	Uncharacterized protein OS=Oryza sativa subsp. japonica OX=39947 GN=OsJ_14740 PE=3 SV=1	12.76595745	2	2	18000000	0.468	2
A2Z9J2	Uncharacterized protein OS=Oryza sativa subsp. indica OX=39946 GN=OsI_34391 PE=3 SV=1	9.053497942	2	3	4900000	0.501	2
A2YL42	Uncharacterized protein OS=Oryza sativa subsp. indica OX=39946 GN=OsI_25933 PE=4 SV=1	8.050847458	2	6	480000000	0.389	2
B8B879	Uncharacterized protein OS=Oryza sativa subsp. indica OX=39946 GN=OsI_25294 PE=4 SV=1	4.381846635	2	2	3000000	0.141	2
B8AYT9	Uncharacterized protein OS=Oryza sativa subsp. indica OX=39946 GN=OsI_20192 PE=4 SV=1	11.93548387	2	2	2200000	0.245	2

A2XY57	Uncharacterized protein OS=Oryza sativa subsp. indica OX=39946 GN=OsI_17640 PE=3 SV=1	7.434944238	2	2	2300000	0.259	2
B8AQH5	Uncharacterized protein OS=Oryza sativa subsp. indica OX=39946 GN=OsI_10423 PE=3 SV=1	7.581227437	2	2	2800000	0.274	2
B8AF05	Uncharacterized protein OS=Oryza sativa subsp. indica OX=39946 GN=OsI_07935 PE=4 SV=1	10.33333333	2	2	12000000	0.245	2
A2WLL0	Uncharacterized protein OS=Oryza sativa subsp. indica OX=39946 GN=OsI_00725 PE=4 SV=1	9.240924092	2	2	4900000	0.292	2
A0A0E0HDS0	Uncharacterized protein OS=Oryza nivara OX=4536 PE=4 SV=1	6.547619048	2	3	7300000	0.35	2
A0A0E0FY40	Uncharacterized protein OS=Oryza nivara OX=4536 PE=4 SV=1	6.233062331	2	2	39000000	0.334	2
A0A0E0I922	Uncharacterized protein OS=Oryza nivara OX=4536 PE=4 SV=1	10.34482759	2	2	28000000	0.311	2
A0A0E0FZD2	Uncharacterized protein OS=Oryza nivara OX=4536 PE=4 SV=1	4.149377593	2	2	3100000	0.155	2
A0A0E0G9R0	Uncharacterized protein OS=Oryza nivara OX=4536 PE=4 SV=1	7.397959184	2	2	2800000	0.222	2
A0A0E0J7K7	Uncharacterized protein OS=Oryza nivara OX=4536 PE=4 SV=1	5.540897098	2	2	6300000	0.233	2
A0A0E0IUD2	Uncharacterized protein OS=Oryza nivara OX=4536 PE=4 SV=1	2.069614299	2	2	800000	0.077	2
A0A0E0HC03	Uncharacterized protein OS=Oryza nivara OX=4536 PE=4 SV=1	16.96428571	2	2	6300000	0.778	2
A0A0E0HLZ8	Uncharacterized protein OS=Oryza nivara OX=4536 PE=4 SV=1	4.632152589	2	2	8100000	0.222	2
A0A0E0GCI2	Uncharacterized protein OS=Oryza nivara OX=4536 PE=4 SV=1	6.018518519	2	2	1900000	0.222	2
A0A0E0I640	Uncharacterized protein OS=Oryza nivara OX=4536 PE=3 SV=1	5.474452555	2	3	7000000	0.205	2
A0A0E0H6P6	Uncharacterized protein OS=Oryza nivara OX=4536 PE=3 SV=1	7.021276596	2	3	54000000	0.389	2
A0A0E0GL83	Uncharacterized protein OS=Oryza nivara OX=4536 PE=3 SV=1	9.365558912	2	3	17000000	0.413	2
A0A0E0GQ33	Uncharacterized protein OS=Oryza nivara OX=4536 PE=3 SV=1	11.01928375	2	2	10000000	0.292	2
Q8H8C2	Uncharacterized protein OJ1134F05.7 OS=Oryza sativa subsp. japonica OX=39947 GN=OJ1134F05.7 PE=4 SV=1	7.268170426	2	2	25000000	0.274	2
Q0DIU1	UDP-glucuronate decarboxylase OS=Oryza sativa subsp. japonica OX=39947 GN=Os05g0363200 PE=3 SV=1	6.263982103	2	2	5600000	0.179	2
A0A0E0FLT1	Tyrosine--tRNA ligase OS=Oryza nivara OX=4536 PE=3 SV=1	6.691449814	2	2	3600000	0.141	2
A2ZLB6	Tryptophanyl-tRNA synthetase OS=Oryza sativa subsp. indica OX=39946 GN=OsI_38615 PE=3 SV=1	6.862745098	2	2	7400000	0.179	2
A2YR01	Tryptophan synthase OS=Oryza sativa subsp. indica OX=39946 GN=OsI_27728 PE=3 SV=1	7.531380753	2	2	14000000	0.172	2
A0A0E0GX58	Sulfate adenylyltransferase OS=Oryza nivara OX=4536 PE=4 SV=1	8.193277311	2	2	23000000	0.15	2
B8BIW2	Sacchrp_dh_NADP domain-containing protein OS=Oryza sativa subsp. indica OX=39946 GN=OsI_35044 PE=4 SV=1	5.386416862	2	2	21000000	0.194	2
A0A0E0HQ12	RuvB-like helicase OS=Oryza nivara OX=4536 PE=3 SV=1	5.870020964	2	2	13000000	0.179	2
Q94I53	Putative hydrolase OS=Oryza sativa subsp. japonica OX=39947 GN=OSJNBa0084C09.19 PE=4 SV=1	6.527415144	2	2	16000000	0.222	2
B9G712	Protein-methionine-S-oxide reductase OS=Oryza sativa subsp. japonica OX=39947 GN=OsJ_32480 PE=3 SV=1	2.5	2	2	15000000	0.194	2
Q6ETQ7	Protein THYLAKOID RHODANESE-LIKE, chloroplastic OS=Oryza sativa subsp. japonica OX=39947 GN=TROL PE=1 SV=1	6.90376569	2	2	3500000	0.389	2
Q0JD42	Protein disulfide isomerase-like 5-2 OS=Oryza sativa subsp. japonica OX=39947 GN=PDIL5-2 PE=2 SV=2	6.146572104	2	2	27000000	0.233	2
Q6Z6M0	Protein CLP1 homolog OS=Oryza sativa subsp. japonica OX=39947 GN=Os02g0217500 PE=3 SV=1	7.175925926	2	2	7200000	0.245	2
Q651D5	Probable aquaporin PIP2-7 OS=Oryza sativa subsp. japonica OX=39947 GN=PIP2-7 PE=2 SV=2	12.06896552	2	2	16000000	0.52	2
A0A0E0HNE8	Probable alanine--tRNA ligase, chloroplastic OS=Oryza nivara OX=4536 PE=3 SV=1	3.012048193	2	2	7100000	0.075	2
Q69WE1	Plastid-lipid associated protein PAP/fibrillin family-like OS=Oryza sativa subsp. japonica OX=39947 GN=OJ1103_E04.105 PE=4 SV=1	5.429864253	2	2	11000000	0.16	2
Q7M1Y7	Photosystem II oxygen-evolving complex protein 2 (Fragment) OS=Oryza sativa OX=4530 PE=1 SV=1	72.97297297	2	2	43000000	9	2
D0EKL9	Photosystem II CP47 reaction center protein OS=Oryza sativa OX=4530 GN=psbB PE=3 SV=1	5.511811024	2	2	17000000	0.259	2
A0A0E0GPD6	Phosphopyruvate hydratase OS=Oryza nivara OX=4536 PE=3 SV=1	6.085192698	2	2	22000000	0.155	2
A0A0E0FS04	Phospholipase A1 OS=Oryza nivara OX=4536 PE=3 SV=1	7.484407484	2	3	15000000	0.369	2
A0A0P0XH73	Phospho-2-dehydro-3-deoxyheptonate aldolase OS=Oryza sativa subsp. japonica OX=39947 GN=Os08g0484500 PE=3 SV=1	4.356435644	2	2	3600000	0.141	2
Q10SM7	Peroxisomal membrane protein 11-1 OS=Oryza sativa subsp. japonica OX=39947 GN=PEX11-1 PE=2 SV=1	10.97046414	2	2	16000000	0.425	2
Q6ER49	Peroxidase OS=Oryza sativa subsp. japonica OX=39947 GN=prx29 PE=2 SV=1	9.345794393	2	2	10000000	0.334	2
A0A0E0FU51	Peptidase_M48 domain-containing protein OS=Oryza nivara OX=4536 PE=4 SV=1	6.52173913	2	3	12000000	0.28	2
A0A0E0H2B2	PCI domain-containing protein OS=Oryza nivara OX=4536 PE=4 SV=1	6.683804627	2	3	16000000	0.318	2
A0A0E0FM52	PCI domain-containing protein OS=Oryza nivara OX=4536 PE=3 SV=1	5.050505051	2	2	9100000	0.233	2
A2YFA8	PALP domain-containing protein OS=Oryza sativa subsp. indica OX=39946 GN=OsI_23796 PE=3 SV=1	6.981519507	2	2	5100000	0.212	2
Q0IRL0	Os11g0620100 protein OS=Oryza sativa subsp. japonica OX=39947 GN=Os11g0620100 PE=4 SV=1	4.988662132	2	2	5700000	0.233	2
Q0J3Q9	Os08g0558200 protein OS=Oryza sativa subsp. japonica OX=39947 GN=Os08g0558200 PE=4 SV=1	8.504398827	2	2	37000000	0.359	2
A3BJK2	Os07g0468100 protein OS=Oryza sativa subsp. japonica OX=39947 GN=Os07g0468100 PE=4 SV=1	9.051724138	2	2	16000000	0.259	2
A0A0N7KKH3	Os05g0299200 protein OS=Oryza sativa subsp. japonica OX=39947 GN=Os05g0299200 PE=4 SV=1	6.653225806	2	2	18000000	0.172	2
Q5WMY3	Os05g0155100 protein OS=Oryza sativa subsp. japonica OX=39947 GN=Os05g0155100 PE=2 SV=1	17.55319149	2	2	18000000	0.259	2
Q0JAF4	Os04g0602100 protein OS=Oryza sativa subsp. japonica OX=39947 GN=Os04g0602100 PE=2 SV=1	5.66572238	2	2	4700000	0.212	2
A0A0P0WCZ7	Os04g0542900 protein OS=Oryza sativa subsp. japonica OX=39947 GN=Os04g0542900 PE=3 SV=1	5.973025048	2	2	12000000	0.15	2
Q6H6L8	Os02g0662100 protein OS=Oryza sativa subsp. japonica OX=39947 GN=Os02g0662100 PE=2 SV=1	17.96875	2	2	7500000	1.154	2
Q6ETT3	Os02g0108400 protein OS=Oryza sativa subsp. japonica OX=39947 GN=Os02g0108400 PE=4 SV=1	6.201550388	2	2	8600000	0.274	2

A2ZXU2	Os01g0749200 protein OS=Oryza sativa subsp. japonica OX=39947 GN=Os01g0749200 PE=3 SV=1	15.02145923	2	4	18000000	0.719	2
Q0JL78	Os01g0617900 protein (Fragment) OS=Oryza sativa subsp. japonica OX=39947 GN=Os01g0617900 PE=4 SV=1	7.645259939	2	2	6800000	0.311	2
Q8LJ81	Os01g0581300 protein OS=Oryza sativa subsp. japonica OX=39947 GN=Os01g0581300 PE=2 SV=1	3.518518519	2	2	3200000	0.166	2
A0A0K0LL12	NAD(P)H-quinone oxidoreductase subunit K, chloroplastic OS=Oryza sativa OX=4530 GN=ndhK PE=3 SV=1	18.3745583	2	2	14000000	0.334	2
B9F1E6	MSP domain-containing protein OS=Oryza sativa subsp. japonica OX=39947 GN=OsJ_07762 PE=3 SV=1	3.249097473	2	2	87000000	0.16	2
A0A0E0IU06	Malate dehydrogenase OS=Oryza nivara OX=4536 PE=3 SV=1	6.297229219	2	2	2200000	0.222	2
A0A0E0IFV5	Malate dehydrogenase (NADP(+)) OS=Oryza nivara OX=4536 PE=3 SV=1	6.04288499	2	2	13000000	0.141	2
A0A0E0GDA6	M16C_associated domain-containing protein OS=Oryza nivara OX=4536 PE=3 SV=1	3.617810761	2	2	12000000	0.071	2
A0A0E0G8B3	Lipase_3 domain-containing protein OS=Oryza nivara OX=4536 PE=4 SV=1	5.844155844	2	2	7100000	0.16	2
B8B8V3	Jacalin-type lectin domain-containing protein OS=Oryza sativa subsp. indica OX=39946 GN=OsI_29960 PE=4 SV=1	5.405405405	2	2	8800000	0.16	2
Q8H3C9	IAA-amino acid hydrolase ILR1-like 7 OS=Oryza sativa subsp. japonica OX=39947 GN=ILL7 PE=2 SV=1	5.934065934	2	2	2100000	0.202	2
A0A0E0GFD0	Homoserine kinase OS=Oryza nivara OX=4536 PE=3 SV=1	9.319899244	2	2	4200000	0.292	2
A0A0E0JBK9	Histone H4 OS=Oryza nivara OX=4536 PE=3 SV=1	21.3592233	2	2	12000000	1.154	2
A0A5C8LKB7	GTP-binding protein (Fragment) OS=Rheinheimera tangshanensis OX=400153 GN=FU839_18715 PE=4 SV=1	21.9858156	2	3	44000000	1.371	2
Q6Z7F5	Glutathione transferase OS=Oryza sativa subsp. japonica OX=39947 GN=Os02g0564000 PE=2 SV=1	10.97046414	2	2	5300000	0.274	2
A0A0E0G0L2	Glutaminase OS=Oryza nivara OX=4536 PE=3 SV=1	3.676470588	2	2	5800000	0.096	2
A2YW09	Glutamate decarboxylase OS=Oryza sativa subsp. indica OX=39946 GN=OsI_29517 PE=3 SV=1	4.085603113	2	2	6900000	0.155	2
A0A0E0H6D1	Epimerase domain-containing protein OS=Oryza nivara OX=4536 PE=4 SV=1	7.803468208	2	2	12000000	0.259	2
A6N0I8	Elongation factor 1-alpha (Fragment) OS=Oryza sativa subsp. indica OX=39946 PE=2 SV=1	44.89795918	2	2	2800000	3.642	2
B8AMP4	Dolichol-phosphate mannosyltransferase subunit 1 OS=Oryza sativa subsp. indica OX=39946 GN=OsI_14131 PE=3 SV=1	13.99176955	2	2	12000000	0.311	2
A3ATC6	Dihydrolipoyllysine-residue succinyltransferase OS=Oryza sativa subsp. japonica OX=39947 GN=Os04g0394200 PE=3 SV=1	5	2	2	6800000	0.222	2
A0A5C8LNP2	Dihydrolipoyllysine-residue succinyltransferase component of 2-oxoglutarate dehydrogenase complex OS=Rheinheimera tangshanensis OX=	4.545454545	2	2	10000000	0.245	2
A0A0E0GE00	CYTOSOL_AP domain-containing protein OS=Oryza nivara OX=4536 PE=3 SV=1	5.351170569	2	2	11000000	0.15	2
B8B8S6	CpSecY OS=Oryza sativa subsp. indica OX=39946 GN=OsI_28473 PE=3 SV=1	2.857142857	2	2	7500000	0.086	2
A0A5C8LLI6	Citrate synthase OS=Rheinheimera tangshanensis OX=400153 GN=gltA PE=3 SV=1	3.529411765	2	2	11000000	0.212	2
B8B9C5	Cinnamyl-alcohol dehydrogenase OS=Oryza sativa subsp. indica OX=39946 GN=OsI_30129 PE=3 SV=1	5.434782609	2	3	14000000	0.389	2
A0A172MA97	Chloroplast 9-cis-epoxycarotenoid dioxygenase 1 OS=Oryza sativa subsp. indica OX=39946 PE=2 SV=1	4.07523511	2	2	15000000	0.141	2
A0A0E0I0Q4	Carboxypeptidase OS=Oryza nivara OX=4536 PE=3 SV=1	4.770992366	2	2	8500000	0.179	2
B8A8D2	Bifunctional nuclease 1 OS=Oryza sativa subsp. indica OX=39946 GN=BBD1 PE=3 SV=1	7.552870091	2	2	4800000	0.245	2
B8AK88	Aspartate-semialdehyde dehydrogenase OS=Oryza sativa subsp. indica OX=39946 GN=OsI_13624 PE=3 SV=1	10.93333333	2	3	5300000	0.438	2
B8BF46	Annexin OS=Oryza sativa subsp. indica OX=39946 GN=OsI_31267 PE=3 SV=1	6.59025788	2	2	7900000	0.194	2
A0A0E0I4C3	Amino_oxidase domain-containing protein OS=Oryza nivara OX=4536 PE=4 SV=1	6.342494715	2	2	4500000	0.186	2
A0A0E0HEW9	Amidophosphoribosyltransferase OS=Oryza nivara OX=4536 PE=3 SV=1	4.990757856	2	2	3100000	0.15	2
A0A0B4U1V7	Aldehyde dehydrogenase (Fragment) OS=Oryza sativa OX=4530 GN=LOC_Os06g15990.1 PE=2 SV=1	6.193078324	2	2	12000000	0.136	2
A0A0E0GVZ3	AIR synthase OS=Oryza nivara OX=4536 PE=3 SV=1	6.724511931	2	2	4000000	0.194	2
A0A0E0GAG0	AB hydrolase-1 domain-containing protein OS=Oryza nivara OX=4536 PE=4 SV=1	8.968609865	2	2	5100000	0.245	2
B8AGL8	AAA domain-containing protein OS=Oryza sativa subsp. indica OX=39946 GN=OsI_06998 PE=3 SV=1	4.977375566	2	2	11000000	0.212	2
A0A0E0GDS7	AAA domain-containing protein OS=Oryza nivara OX=4536 PE=3 SV=1	6.103286385	2	2	9200000	0.166	2
A0A0E0FT82	60S ribosomal protein L18a OS=Oryza nivara OX=4536 PE=3 SV=1	10.41666667	2	3	6300000	0.585	2
A0A0K0K9A6	4-hydroxy-tetrahydrodipicolinate synthase OS=Oryza sativa OX=4530 GN=DHDPS PE=2 SV=1	5.789473684	2	2	4200000	0.259	2
A2XHR6	40S ribosomal protein SA OS=Oryza sativa subsp. indica OX=39946 GN=OsI_11954 PE=3 SV=1	10	2	4	45000000	0.719	2
A2Y8X9	3-phosphoshikimate 1-carboxyvinyltransferase OS=Oryza sativa subsp. indica OX=39946 GN=EPSPS PE=2 SV=1	5.436893204	2	2	20000000	0.194	2
Q9FEB6	26S proteasome ATPase subunit Rpt6 OS=Oryza sativa OX=4530 GN=OsRpt6 PE=2 SV=1	6.839622642	2	2	7900000	0.172	2
A2YWB4	14_3_3 domain-containing protein OS=Oryza sativa subsp. indica OX=39946 GN=OsI_29626 PE=3 SV=1	10.60606061	2	2	6300000	0.259	2
A0A0E0GSM8	14_3_3 domain-containing protein OS=Oryza nivara OX=4536 PE=3 SV=1	7.954545455	2	2		0.233	2
A3BHC0	WD_REPEATS_REGION domain-containing protein OS=Oryza sativa subsp. japonica OX=39947 GN=OsJ_23379 PE=4 SV=1	2.81030445	1	1	5400000	0.089	1
A0A0E0FRZ9	WD_REPEATS_REGION domain-containing protein OS=Oryza nivara OX=4536 PE=3 SV=1	2.964426877	1	1	11000000	0.089	1
B8B782	V-type proton ATPase subunit H OS=Oryza sativa subsp. indica OX=39946 GN=OsI_26411 PE=3 SV=1	2.626262626	1	1		0.077	1
A0A0E0ITM5	Vesicle-fusing ATPase OS=Oryza nivara OX=4536 PE=3 SV=1	1.314060447	1	1		0.055	1
A0A0E0GM62	Uroporphyrinogen_deCOase domain-containing protein OS=Oryza nivara OX=4536 PE=4 SV=1	3.703703704	1	1	10000000	0.072	1
A0A542P617	Uncharacterized protein OS=Streptomyces sp. SLBN-109 OX=2768451 GN=FBY32_1181 PE=4 SV=1	0.67834935	1	1	3600000	0.022	1
A0A7W7XW70	Uncharacterized protein OS=Streptomyces scabiei OX=1930 GN=FHR26_006661 PE=4 SV=1	3.529411765	1	1	6500000	0.101	1
B9FVD6	Uncharacterized protein OS=Oryza sativa subsp. japonica OX=39947 GN=OsJ_23002 PE=3 SV=1	1.966717095	1	1		0.062	1
B9FPB7	Uncharacterized protein OS=Oryza sativa subsp. japonica OX=39947 GN=OsJ_18363 PE=4 SV=1	3.434343434	1	1	8400000	0.08	1

A3AMD8	Uncharacterized protein OS=Oryza sativa subsp. japonica OX=39947 GN=OsJ_12459 PE=3 SV=1	2.504173623	1	1	2600000	0.066	1
B9F9W5	Uncharacterized protein OS=Oryza sativa subsp. japonica OX=39947 GN=OsJ_11852 PE=3 SV=1	2.981651376	1	1		0.08	1
A2ZQ33	Uncharacterized protein OS=Oryza sativa subsp. japonica OX=39947 GN=OsJ_00667 PE=3 SV=1	2.625298329	1	1	9000000	0.086	1
Q2QP89	Uncharacterized protein OS=Oryza sativa subsp. japonica OX=39947 GN=LOC_Os12g35310 PE=4 SV=1	6.299212598	1	1	4600000	0.155	1
B8BP98	Uncharacterized protein OS=Oryza sativa subsp. indica OX=39946 GN=OsI_38079 PE=3 SV=1	3.641456583	1	1	1400000	0.116	1
B8BGS7	Uncharacterized protein OS=Oryza sativa subsp. indica OX=39946 GN=OsI_33537 PE=3 SV=1	3	1	1		0.096	1
A2YYA0	Uncharacterized protein OS=Oryza sativa subsp. indica OX=39946 GN=OsI_30326 PE=4 SV=1	8.8	1	1	6100000	0.389	1
A2YML2	Uncharacterized protein OS=Oryza sativa subsp. indica OX=39946 GN=OsI_26464 PE=3 SV=1	4.87804878	1	1	18000000	0.155	1
B8AWA2	Uncharacterized protein OS=Oryza sativa subsp. indica OX=39946 GN=OsI_20868 PE=4 SV=1	3.174603175	1	1	12000000	0.068	1
B8B098	Uncharacterized protein OS=Oryza sativa subsp. indica OX=39946 GN=OsI_20774 PE=3 SV=1	3.928571429	1	1	12000000	0.179	1
A2XRE0	Uncharacterized protein OS=Oryza sativa subsp. indica OX=39946 GN=OsI_15197 PE=3 SV=1	16.10169492	1	1	3600000	0.259	1
A2XNL2	Uncharacterized protein OS=Oryza sativa subsp. indica OX=39946 GN=OsI_14156 PE=3 SV=1	8.823529412	1	1	15000000	0.212	1
A2XN99	Uncharacterized protein OS=Oryza sativa subsp. indica OX=39946 GN=OsI_14032 PE=4 SV=1	4.237288136	1	1	4900000	0.11	1
B8AMI1	Uncharacterized protein OS=Oryza sativa subsp. indica OX=39946 GN=OsI_11265 PE=4 SV=1	3.06122449	1	1	1600000	0.058	1
A2XEZ1	Uncharacterized protein OS=Oryza sativa subsp. indica OX=39946 GN=OsI_10906 PE=4 SV=1	4.545454545	1	1	4300000	0.145	1
B8AAM5	Uncharacterized protein OS=Oryza sativa subsp. indica OX=39946 GN=OsI_00945 PE=3 SV=1	3.463203463	1	1	7100000	0.136	1
A0A0E0FN63	Uncharacterized protein OS=Oryza nivara OX=4536 PE=4 SV=1	7.920792079	1	1	13000000	0.136	1
A0A0E0H354	Uncharacterized protein OS=Oryza nivara OX=4536 PE=4 SV=1	0.954032958	1	1	7000000	0.032	1
A0A0E0IKK6	Uncharacterized protein OS=Oryza nivara OX=4536 PE=4 SV=1	8.59375	1	1	7300000	0.468	1
A0A0E0GWD1	Uncharacterized protein OS=Oryza nivara OX=4536 PE=4 SV=1	3.166226913	1	1	6600000	0.129	1
A0A0E0IV04	Uncharacterized protein OS=Oryza nivara OX=4536 PE=4 SV=1	2.177554439	1	1	8100000	0.07	1
A0A0E0G0C5	Uncharacterized protein OS=Oryza nivara OX=4536 PE=4 SV=1	3.90070922	1	1	6600000	0.166	1
A0A0E0I3T2	Uncharacterized protein OS=Oryza nivara OX=4536 PE=4 SV=1	10.625	1	1	7600000	0.166	1
A0A0E0GZV4	Uncharacterized protein OS=Oryza nivara OX=4536 PE=4 SV=1	3.084832905	1	1	2700000	0.105	1
A0A0E0HE02	Uncharacterized protein OS=Oryza nivara OX=4536 PE=4 SV=1	0.92236741	1	1	16000000	0.039	1
A0A0E0I610	Uncharacterized protein OS=Oryza nivara OX=4536 PE=4 SV=1	1.106639839	1	1	2900000	0.044	1
A0A0E0FY52	Uncharacterized protein OS=Oryza nivara OX=4536 PE=4 SV=1	1.992753623	1	1		0.077	1
A0A0E0FLY5	Uncharacterized protein OS=Oryza nivara OX=4536 PE=4 SV=1	2.828618968	1	1	5200000	0.07	1
A0A0E0FRJ9	Uncharacterized protein OS=Oryza nivara OX=4536 PE=4 SV=1	4.225352113	1	1	7400000	0.155	1
A0A0E0HN25	Uncharacterized protein OS=Oryza nivara OX=4536 PE=4 SV=1	2.122641509	1	1	1700000	0.083	1
A0A0E0I5T3	Uncharacterized protein OS=Oryza nivara OX=4536 PE=4 SV=1	3.20855615	1	1	2500000	0.089	1
A0A0E0J795	Uncharacterized protein OS=Oryza nivara OX=4536 PE=4 SV=1	2.811950791	1	1	6800000	0.086	1
A0A0E0FG22	Uncharacterized protein OS=Oryza nivara OX=4536 PE=4 SV=1	1.792114695	1	1	6100000	0.042	1
A0A0E0FV30	Uncharacterized protein OS=Oryza nivara OX=4536 PE=4 SV=1	2.93398533	1	1	3700000	0.089	1
A0A0E0H393	Uncharacterized protein OS=Oryza nivara OX=4536 PE=3 SV=1	4.827586207	1	1	9700000	0.233	1
A0A0E0IQI1	Uncharacterized protein OS=Oryza nivara OX=4536 PE=3 SV=1	4.021447721	1	1	14000000	0.11	1
A0A0E0I0Z1	Uncharacterized protein OS=Oryza nivara OX=4536 PE=3 SV=1	6.213017751	1	1	9700000	0.136	1
A0A0E0IUY0	Uncharacterized protein OS=Oryza nivara OX=4536 PE=3 SV=1	4.672897196	1	1	13000000	0.07	1
A0A0E0IFU5	Uncharacterized protein OS=Oryza nivara OX=4536 PE=3 SV=1	14.77832512	1	1	31000000	0.166	1
A0A0E0FVA1	Uncharacterized protein OS=Oryza nivara OX=4536 PE=3 SV=1	3.25732899	1	1	2100000	0.07	1
A0A0E0GM85	Uncharacterized protein OS=Oryza nivara OX=4536 PE=3 SV=1	9.160305344	1	1	7600000	0.334	1
A0A0E0GB32	Uncharacterized protein OS=Oryza nivara OX=4536 PE=3 SV=1	1.643192488	1	1	3300000	0.053	1
A0A0E0GAW1	Uncharacterized protein OS=Oryza nivara OX=4536 PE=3 SV=1	5.044510386	1	1	2500000	0.194	1
A0A0E0IXN2	Uncharacterized protein OS=Oryza nivara OX=4536 PE=3 SV=1	6.711409396	1	1	5900000	0.334	1
A0A0E0FL87	Uncharacterized protein OS=Oryza nivara OX=4536 PE=3 SV=1	6.25	1	1	9700000	0.212	1
A0A0E0HC73	Uncharacterized protein OS=Oryza nivara OX=4536 PE=3 SV=1	1.886792453	1	1	2300000	0.064	1
A0A0E0FY60	Uncharacterized protein OS=Oryza nivara OX=4536 PE=3 SV=1	6.172839506	1	1	3500000	0.194	1
A0A0E0FPJ8	Uncharacterized protein OS=Oryza nivara OX=4536 PE=3 SV=1	2.830188679	1	1	1300000	0.096	1
A0A0E0G4K8	Uncharacterized protein OS=Oryza nivara OX=4536 PE=3 SV=1	3.271028037	1	1		0.233	1
A0A0E0J925	Uncharacterized protein OS=Oryza nivara OX=4536 PE=3 SV=1	1.698113208	1	1	560000	0.07	1
A0A0E0FQJ9	Uncharacterized protein OS=Oryza nivara OX=4536 PE=3 SV=1	9.189189189	1	1	1600000	0.233	1
Q84QA8	Uncharacterized protein OJ1012B02.13 OS=Oryza sativa subsp. japonica OX=39947 GN=OJ1012B02.13 PE=3 SV=1	2.582159624	1	1	6600000	0.096	1
A2Y538	U-box domain-containing protein OS=Oryza sativa subsp. indica OX=39946 GN=OsI_20111 PE=4 SV=1	2.654867257	1	1	1400000	0.093	1

A0A0E0FSZ9	Tryptophanyl-tRNA synthetase OS=Oryza nivara OX=4536 PE=3 SV=1	4.187192118	1	1	3400000	0.11	1
A0A0E0GX73	Tr-type G domain-containing protein OS=Oryza nivara OX=4536 PE=3 SV=1	1.423487544	1	1		0.046	1
Q2RAH8	Transposon protein, putative, unclassified OS=Oryza sativa subsp. japonica OX=39947 GN=LOC_Os11g05340 PE=4 SV=1	2.278481013	1	1	5500000	0.032	1
Q2R3R6	Transposon protein, putative, Pong sub-class OS=Oryza sativa subsp. japonica OX=39947 GN=LOC_Os11g31020 PE=4 SV=1	1.677852349	1	1	11000000	0.086	1
P93435	Transmembrane protein OS=Oryza sativa OX=4530 PE=2 SV=1	6.529209622	1	1	39000000	0.233	1
B8AJF3	Threonyl-tRNA synthetase OS=Oryza sativa subsp. indica OX=39946 GN=OsI_07557 PE=3 SV=1	1.78041543	1	1	1700000	0.051	1
B9GC77	Thioredoxin domain-containing protein OS=Oryza sativa subsp. japonica OX=39947 GN=OsJ_35463 PE=4 SV=1	3.603603604	1	1	3200000	0.083	1
A0A0E0GZS1	SWIB domain-containing protein OS=Oryza nivara OX=4536 PE=4 SV=1	1.672240803	1	1	2200000	0.075	1
B8AWM4	Superoxide dismutase OS=Oryza sativa subsp. indica OX=39946 GN=OsI_19472 PE=3 SV=1	6.060606061	1	1	14000000	0.212	1
A2XLW5	Sulfate adenylyltransferase OS=Oryza sativa subsp. indica OX=39946 GN=OsI_13470 PE=4 SV=1	2.935010482	1	1	2200000	0.08	1
A2YH94	SOR_SNZ domain-containing protein OS=Oryza sativa subsp. indica OX=39946 GN=OsI_24560 PE=3 SV=1	4.098360656	1	1	4300000	0.101	1
Q10M68	Serologically defined breast cancer antigen NY-BR-84, putative, expressed OS=Oryza sativa subsp. japonica OX=39947 GN=LOC_Os03g20520	2.067183463	1	1	1200000	0.116	1
Q7Y0B9	Serine/threonine-protein kinase SAPK8 OS=Oryza sativa subsp. japonica OX=39947 GN=SAPK8 PE=1 SV=1	3.504043127	1	1	4100000	0.116	1
A0A542HVU7	S-DNA-T family DNA segregation ATPase FtsK/SpoIIIE OS=Streptomyces sp. SLBN-115 OX=2768453 GN=FBY34_3941 PE=4 SV=1	0.681302044	1	1	77000000	0.035	1
A0A0E0ISY9	SAP domain-containing protein OS=Oryza nivara OX=4536 PE=3 SV=1	0.847457627	1	1	4900000	0.028	1
A2WMG6	Salt stress root protein RS1 OS=Oryza sativa subsp. indica OX=39946 GN=OsI_001009 PE=1 SV=1	6.37254902	1	1	7500000	0.179	1
A3CG62	Rubis-subs-bind domain-containing protein OS=Oryza sativa subsp. japonica OX=39947 GN=OsJ_35675 PE=4 SV=1	2.052238806	1	1	3000000	0.089	1
B9FAQ8	RNase H domain-containing protein OS=Oryza sativa subsp. japonica OX=39947 GN=OsJ_12207 PE=4 SV=1	2.558635394	1	1	11000000	0.083	1
A0A0E0GJR0	Ribosomal_S10 domain-containing protein OS=Oryza nivara OX=4536 PE=3 SV=1	2.04778157	1	1	6500000	0.07	1
B9FXS4	RF_PROK_I domain-containing protein OS=Oryza sativa subsp. japonica OX=39947 GN=OsJ_24645 PE=3 SV=1	1.606425703	1	1	2600000	0.086	1
A0A0E0FST5	Reticulon-like protein OS=Oryza nivara OX=4536 PE=4 SV=1	4.347826087	1	1	6900000	0.212	1
A0A0E0IYE1	Pyruvate kinase OS=Oryza nivara OX=4536 PE=3 SV=1	2.277039848	1	2	7300000	0.066	1
A2Z2Z0	Pyruvate dehydrogenase E1 component subunit beta OS=Oryza sativa subsp. indica OX=39946 GN=OsI_31986 PE=4 SV=1	2.925531915	1	1	1300000	0.136	1
Q10CS5	Putative strictosidine synthase OS=Oryza sativa subsp. japonica OX=39947 GN=OSJNBa0047E24.22 PE=3 SV=1	3.958333333	1	1	26000000	0.077	1
Q84TZ5	Putative L-asparaginase OS=Oryza sativa subsp. japonica OX=39947 GN=OSJNBa0087M10.11 PE=4 SV=1	3.571428571	1	1	7600000	0.145	1
Q9AV27	Putative cytochrome P450 monooxygenase OS=Oryza sativa subsp. japonica OX=39947 GN=OSJNBa0001O14.16 PE=3 SV=1	2.739726027	1	1		0.068	1
Q6Z2T4	Putative ATPase OS=Oryza sativa subsp. japonica OX=39947 GN=OJ1118_G04.14 PE=4 SV=1	3.201970443	1	2	16000000	0.202	1
Q8GZY2	Putative 50S ribosomal protein L10 OS=Oryza sativa subsp. japonica OX=39947 GN=OSJNBa0013D02.20 PE=3 SV=1	4.506437768	1	1		0.086	1
A0A0E0JBR9	PSI-K OS=Oryza nivara OX=4536 PE=3 SV=1	6.766917293	1	1		0.334	1
A0A0P0XNM8	PSI-G (Fragment) OS=Oryza sativa subsp. japonica OX=39947 GN=Os09g0481200 PE=3 SV=1	4.90797546	1	1	43000000	0.259	1
Q10A77	Protein TIC 62, chloroplastic OS=Oryza sativa subsp. japonica OX=39947 GN=TIC62 PE=1 SV=1	3.420523139	1	1	5200000	0.122	1
A0A542VEZ4	Protein RecA OS=Pseudomonas sp. SLBN-26 OX=2768443 GN=recA PE=3 SV=1	3.142857143	1	1	15000000	0.11	1
A2XJY2	Protein kinase domain-containing protein OS=Oryza sativa subsp. indica OX=39946 GN=OsI_12749 PE=4 SV=1	2.644230769	1	1		0.089	1
A0A0E0HCN8	Protein kinase domain-containing protein OS=Oryza nivara OX=4536 PE=4 SV=1	0.948509485	1	1	1800000	0.051	1
A0A0E0IM33	Proteasome subunit beta OS=Oryza nivara OX=4536 PE=3 SV=1	3.623188406	1	1	2900000	0.136	1
A0A0E0FI12	Proteasome subunit alpha type OS=Oryza nivara OX=4536 PE=3 SV=1	6.4	1	1		0.212	1
A0A5S6RAD1	Prolyl-tRNA synthetase OS=Oryza sativa subsp. japonica OX=39947 GN=Os12g0443700 PE=3 SV=1	2.352941176	1	1	3000000	0.072	1
A0A0P0XIH5	Polyadenylate-binding protein OS=Oryza sativa subsp. japonica OX=39947 GN=Os09g0115400 PE=3 SV=1	1.812688822	1	1	1200000	0.072	1
A0A0E0HJH9	Plastocyanin OS=Oryza nivara OX=4536 PE=3 SV=1	15.58441558	1	1	30000000	0.468	1
A0A0E0GFI5	PKS_ER domain-containing protein OS=Oryza nivara OX=4536 PE=4 SV=1	4	1	1	3400000	0.105	1
B9FZJ0	Photosystem II 10 kDa polypeptide, chloroplastic OS=Oryza sativa subsp. japonica OX=39947 GN=OsJ_26372 PE=3 SV=1	13.42281879	1	1		0.468	1
A2Y7D9	Photosystem I reaction center subunit VI, chloroplastic OS=Oryza sativa subsp. indica OX=39946 GN=PSAH PE=2 SV=1	7.746478873	1	1	1100000	0.334	1
E9KIP0	Photosystem I P700 chlorophyll a apoprotein A2 OS=Oryza sativa subsp. japonica OX=39947 GN=psaB PE=3 SV=1	1.62601626	1	1	9400000	0.116	1
Q7M1U9	Photosystem I 9K protein (Fragment) OS=Oryza sativa OX=4530 PE=1 SV=1	18	1	1	28000000	1.154	1
A0A0E0IMS9	Photolyase/cryptochrome alpha/beta domain-containing protein OS=Oryza nivara OX=4536 PE=4 SV=1	1.945525292	1	1	5000000	0.062	1
A2WUX9	Phosphotransferase OS=Oryza sativa subsp. indica OX=39946 GN=OsI_03691 PE=3 SV=1	3.162055336	1	1	3200000	0.086	1
B9FRD6	Phosphopyruvate hydratase OS=Oryza sativa subsp. japonica OX=39947 GN=OsJ_20044 PE=3 SV=1	3.246753247	1	1	5100000	0.077	1
A0A543GM41	Phospholipase/carboxylesterase OS=Rhizobium sp. SLBN-4 OX=2768441 GN=FBY19_4185 PE=4 SV=1	2.13592233	1	2	2900000	0.089	1
Q2QTC2	Phosphoglucan, water dikinase, chloroplastic OS=Oryza sativa subsp. japonica OX=39947 GN=GWD3 PE=3 SV=2	1.575456053	1	1	2500000	0.03	1
A0A0E0I7F4	PHB domain-containing protein OS=Oryza nivara OX=4536 PE=3 SV=1	4.24403183	1	1	5600000	0.096	1
A0A0E0H918	Peroxidase OS=Oryza nivara OX=4536 PE=3 SV=1	1.231060606	1	1	10000000	0.035	1
B9G0N8	Peptidylprolyl isomerase OS=Oryza sativa subsp. japonica OX=39947 GN=OsJ_27139 PE=4 SV=1	2.52293578	1	1	2200000	0.089	1
Q0DHL6	Peptidylprolyl isomerase OS=Oryza sativa subsp. japonica OX=39947 GN=Os05g0458100 PE=4 SV=1	5.913978495	1	1	5900000	0.389	1
B8ACB8	Peptide deformylase OS=Oryza sativa subsp. indica OX=39946 GN=OsI_02992 PE=3 SV=1	1.003009027	1	1	1100000	0.037	1

A0A0E0GCA6	Peptidase A1 domain-containing protein OS=Oryza nivara OX=4536 PE=3 SV=1	2.401746725	1	1	4700000	0.136	1
A0A0E0IWX1	PAP_fibrillin domain-containing protein OS=Oryza nivara OX=4536 PE=4 SV=1	4.545454545	1	1	16000000	0.179	1
Q7F2H9	p0432B10.9 protein OS=Oryza sativa subsp. japonica OX=39947 GN=P0432B10.9 PE=3 SV=1	4.239401496	1	1	4900000	0.122	1
Q9FP37	p0035H10.3 protein OS=Oryza sativa subsp. japonica OX=39947 GN=P0035H10.3 PE=4 SV=1	2.330097087	1	1	14000000	0.089	1
Q7XM88	OSJNBb0060E08.11 protein OS=Oryza sativa subsp. japonica OX=39947 GN=OSJNBb0060E08.11 PE=3 SV=1	2.100840336	1	1	930000	0.093	1
A0A0P0WBQ3	OSJNBb0011N17.9 protein OS=Oryza sativa subsp. japonica OX=39947 GN=Os04g0481300 PE=4 SV=1	4.850746269	1	1	10000000	0.136	1
Q7XXC5	OSJNBa0027O01.13 protein OS=Oryza sativa subsp. japonica OX=39947 GN=OSJNBa0027O01.13 PE=4 SV=2	0.566572238	1	3	45000000	0.039	1
Q2QXS4	Os12g0145100 protein OS=Oryza sativa subsp. japonica OX=39947 GN=LOC_Os12g05050 PE=2 SV=1	5.462184874	1	1	13000000	0.194	1
A0A0P0XIH3	Os09g0110400 protein OS=Oryza sativa subsp. japonica OX=39947 GN=Os09g0110400 PE=3 SV=1	4.848484848	1	1	5200000	0.155	1
Q6Z1P3	Os08g0566400 protein OS=Oryza sativa subsp. japonica OX=39947 GN=Os08g0566400 PE=2 SV=1	3.015075377	1	1		0.122	1
A0A0P0XBM6	Os08g0109200 protein (Fragment) OS=Oryza sativa subsp. japonica OX=39947 GN=Os08g0109200 PE=4 SV=1	3.738317757	1	1	13000000	0.096	1
A0A0P0X9B9	Os07g0628700 protein OS=Oryza sativa subsp. japonica OX=39947 GN=Os07g0628700 PE=4 SV=1	1.920236337	1	1	4900000	0.064	1
Q0D6M4	Os07g0464200 protein (Fragment) OS=Oryza sativa subsp. japonica OX=39947 GN=Os07g0464200 PE=3 SV=2	4.571428571	1	1	11000000	0.129	1
A0A0N7KN57	Os07g0227800 protein (Fragment) OS=Oryza sativa subsp. japonica OX=39947 GN=Os07g0227800 PE=4 SV=1	1.877133106	1	1	4700000	0.068	1
Q653V9	Os06g0683200 protein OS=Oryza sativa subsp. japonica OX=39947 GN=Os06g0683200 PE=2 SV=1	5.789473684	1	1		0.292	1
Q0DB81	Os06g0594100 protein OS=Oryza sativa subsp. japonica OX=39947 GN=P0652A05.9-1 PE=3 SV=1	2.995391705	1	1	5400000	0.08	1
Q0DF30	Os06g0118700 protein OS=Oryza sativa subsp. japonica OX=39947 GN=Os06g0118700 PE=4 SV=1	1.821862348	1	1		0.129	1
A0A0P0WM00	Os05g0387200 protein OS=Oryza sativa subsp. japonica OX=39947 GN=Os05g0387200 PE=4 SV=1	2.505219207	1	1	2900000	0.077	1
A0A0P0WKZ0	Os05g0333200 protein OS=Oryza sativa subsp. japonica OX=39947 GN=Os05g0333200 PE=3 SV=1	2.820512821	1	1	9400000	0.077	1
Q7XQV1	Os04g0640500 protein OS=Oryza sativa subsp. japonica OX=39947 GN=Os04g0640500 PE=2 SV=2	1.666666667	1	1	3800000	0.062	1
A0A0N7KIF2	Os03g0856500 protein OS=Oryza sativa subsp. japonica OX=39947 GN=Os03g0856500 PE=4 SV=1	5.501618123	1	1		0.129	1
Q0DU42	Os03g0208900 protein OS=Oryza sativa subsp. japonica OX=39947 GN=Os03g0208900 PE=4 SV=1	2.891566265	1	1	7100000	0.105	1
Q6YVH6	Os02g0705100 protein OS=Oryza sativa subsp. japonica OX=39947 GN=Os02g0705100 PE=2 SV=1	8.048780488	1	1	18000000	0.145	1
B9F297	Os02g0129900 protein OS=Oryza sativa subsp. japonica OX=39947 GN=Os02g0129900 PE=4 SV=1	5.863192182	1	1	3800000	0.129	1
Q0JGY3	Os01g0896500 protein OS=Oryza sativa subsp. japonica OX=39947 GN=Os01g0896500 PE=4 SV=1	1.605136437	1	1	4200000	0.086	1
A0A0P0VI36	Nicotianamine aminotransferase 1 OS=Oryza sativa subsp. japonica OX=39947 GN=NAAT1 PE=1 SV=1	2.834008097	1	1	9100000	0.086	1
Q259D2	NADPH-protochlorophyllide oxidoreductase OS=Oryza sativa OX=4530 GN=H0801D08.7 PE=3 SV=1	4.909560724	1	1	1900000	0.086	1
A0A543HP09	NADH-quinone oxidoreductase subunit D OS=Rhizobium sp. SLBN-4 OX=2768441 GN=nuoD PE=3 SV=1	2.777777778	1	1	3500000	0.101	1
A0A0E0FYK0	NAC-A/B domain-containing protein OS=Oryza nivara OX=4536 PE=4 SV=1	4.985337243	1	1	6000000	0.122	1
A0A0E0IXV2	MFS domain-containing protein OS=Oryza nivara OX=4536 PE=3 SV=1	0.803957947	1	1	8300000	0.032	1
A0A0E0H3A0	M20_dimer domain-containing protein OS=Oryza nivara OX=4536 PE=3 SV=1	2.34741784	1	1	2700000	0.096	1
A0A0E0I8J6	Lipoyl synthase, mitochondrial OS=Oryza nivara OX=4536 GN=LIP1 PE=3 SV=1	5.497382199	1	1	3000000	0.11	1
A0A5C8LNV5	Leucine--tRNA ligase OS=Rheinheimera tangshanensis OX=400153 GN=leuS PE=3 SV=1	0.928074246	1	1	97000000	0.047	1
B8B2J4	HTH deoR-type domain-containing protein OS=Oryza sativa subsp. indica OX=39946 GN=OsI_24495 PE=4 SV=1	0.892857143	1	1	7000000	0.041	1
A0A0E0ILW1	Histone deacetylase OS=Oryza nivara OX=4536 PE=4 SV=1	5.417607223	1	1	15000000	0.105	1
A0A542W6S2	Helix-turn-helix protein OS=Streptomyces sp. SLBN-134 OX=2768456 GN=FBY37_1184 PE=4 SV=1	2.651515152	1	1	7100000	0.086	1
A0A0E0GES1	GST C-terminal domain-containing protein OS=Oryza nivara OX=4536 PE=4 SV=1	4.455445545	1	1	1900000	0.105	1
Q8VXC4	Glycine rich RNA binding protein OS=Oryza sativa OX=4530 GN=grp5 PE=2 SV=1	11.8556701	1	1	1700000	0.212	1
A2ZX46	Glycine cleavage system P protein OS=Oryza sativa subsp. japonica OX=39947 GN=OsJ_03218 PE=3 SV=1	1.54589372	1	1	9900000	0.044	1
B8AZE9	Glutathione synthetase OS=Oryza sativa subsp. indica OX=39946 GN=OsI_18978 PE=3 SV=1	2.742230347	1	1	4900000	0.075	1
Q5SMW6	Glutathione peroxidase OS=Oryza sativa subsp. japonica OX=39947 GN=P0568D10.7 PE=3 SV=1	4.979253112	1	1	6200000	0.166	1
A2X1X4	Glutaredoxin-dependent peroxiredoxin OS=Oryza sativa subsp. indica OX=39946 GN=OsI_06200 PE=3 SV=1	4.888888889	1	1	9500000	0.194	1
A0A0E0J7F4	Glutaredoxin domain-containing protein OS=Oryza nivara OX=4536 PE=3 SV=1	4.912280702	1	1	6100000	0.155	1
B9FUN5	Glutamine amidotransferase type-2 domain-containing protein OS=Oryza sativa subsp. japonica OX=39947 GN=OsJ_25434 PE=3 SV=1	0.490496628	1	1	4000000	0.025	1
A0A542RWL8	Glutamate dehydrogenase OS=Nocardioides sp. SLBN-35 OX=2768445 GN=FBY25_2670 PE=3 SV=1	2.684563758	1	1	11000000	0.072	1
A0A0E0GLM4	Glucose-6-phosphate 1-dehydrogenase OS=Oryza nivara OX=4536 PE=3 SV=1	1.597869507	1	1	9900000	0.051	1
Q01K11	Gamma-aminobutyrate transaminase 1, mitochondrial OS=Oryza sativa subsp. indica OX=39946 GN=OsI_17385 PE=3 SV=1	4.84496124	1	1	8700000	0.086	1
A0A0E0I6B3	Ferredoxin OS=Oryza nivara OX=4536 PE=3 SV=1	10.07194245	1	1	31000000	0.292	1
A2YSK1	Fe2OG dioxygenase domain-containing protein OS=Oryza sativa subsp. indica OX=39946 GN=OsI_28301 PE=3 SV=1	4.591836735	1	1	390000	0.093	1
A0A0E0GQJ6	Fe2OG dioxygenase domain-containing protein OS=Oryza nivara OX=4536 PE=3 SV=1	4.261363636	1	1	620000	0.101	1
A0A0E0FTH7	FCP1 homology domain-containing protein OS=Oryza nivara OX=4536 PE=4 SV=1	2.367941712	1	1	5900000	0.08	1
A0A0E0G5U6	FAD-binding FR-type domain-containing protein OS=Oryza nivara OX=4536 PE=4 SV=1	4.081632653	1	1		0.166	1
A0A542RVD4	Exopolyphosphatase/guanosine-5'-triphosphate, 3'-diphosphate pyrophosphatase OS=Nocardioides sp. SLBN-35 OX=2768445 GN=FBY25_24	3.861788618	1	1	14000000	0.064	1
B8B801	eRF1_1 domain-containing protein OS=Oryza sativa subsp. indica OX=39946 GN=OsI_26672 PE=3 SV=1	2.123552124	1	1	4900000	0.077	1

A0A0E0ILW8	E1_dh domain-containing protein OS=Oryza nivara OX=4536 PE=4 SV=1	3.269230769	1	1	4200000	0.077	1
Q851Z9	Dolichyl-phosphate beta-glucosyltransferase OS=Oryza sativa subsp. japonica OX=39947 GN=OSJNBb0081B07.26 PE=3 SV=1	3.157894737	1	1	4300000	0.096	1
B9FW46	Dolichyl-diphosphooligosaccharide--protein glycosyltransferase 48 kDa subunit OS=Oryza sativa subsp. japonica OX=39947 GN=OsJ_23522 P	2.491103203	1	1	9300000	0.08	1
A0A0E0GME8	Divinyl chlorophyllide a 8-vinyl-reductase, chloroplastic OS=Oryza nivara OX=4536 PE=4 SV=1	3.209876543	1	1	7600000	0.116	1
A0A0E0ILV6	Dihydrolipoamide acetyltransferase component of pyruvate dehydrogenase complex OS=Oryza nivara OX=4536 PE=3 SV=1	2.374301676	1	1	3900000	0.062	1
A0A0E0FKX9	Dihydrolipoamide acetyltransferase component of pyruvate dehydrogenase complex OS=Oryza nivara OX=4536 PE=3 SV=1	2.485659656	1	1		0.08	1
A0A0E0GWU8	D-fructose-1,6-bisphosphate 1-phosphohydrolase OS=Oryza nivara OX=4536 PE=3 SV=1	3.740648379	1	1	1300000	0.122	1
A3AQX8	Cysteine synthase OS=Oryza sativa subsp. japonica OX=39947 GN=OsJ_13780 PE=3 SV=1	2.849740933	1	1	7800000	0.11	1
A0A0E0H5U1	Coproporphyrinogen oxidase OS=Oryza nivara OX=4536 PE=3 SV=1	3.25	1	1	20000000	0.083	1
Q6YUR8	Cold shock domain protein 1 OS=Oryza sativa subsp. japonica OX=39947 GN=CSP1 PE=2 SV=1	7.883817427	1	1	5900000	0.155	1
A0A0E0H5V1	Coatomer subunit epsilon OS=Oryza nivara OX=4536 PE=3 SV=1	5.226480836	1	1	2100000	0.129	1
Q01JQ3	Chlorophyll a-b binding protein, chloroplastic OS=Oryza sativa OX=4530 GN=H0523F07.10 PE=3 SV=1	7.142857143	1	1	24000000	0.179	1
A0A0E0ICB1	Chlorophyll a-b binding protein, chloroplastic OS=Oryza nivara OX=4536 PE=3 SV=1	4.508196721	1	1	4200000	0.194	1
A0A0E0G4Q9	Chlorophyll a-b binding protein, chloroplastic OS=Oryza nivara OX=4536 PE=3 SV=1	3.93258427	1	1	13000000	0.166	1
A0A0E0GSA0	Chloride channel protein OS=Oryza nivara OX=4536 PE=3 SV=1	1.167728238	1	1		0.049	1
A0A0E0HNQ9	CAAD domain-containing protein OS=Oryza nivara OX=4536 PE=4 SV=1	7.051282051	1	1	2300000	0.292	1
B9F8X2	Beta-ketoacyl-[acyl-carrier-protein] synthase I OS=Oryza sativa subsp. japonica OX=39947 GN=OsJ_11186 PE=3 SV=1	3.086419753	1	1	7500000	0.101	1
A0A0E0I4B0	ATP-dependent Clp protease proteolytic subunit OS=Oryza nivara OX=4536 PE=3 SV=1	6.376811594	1	2	14000000	0.334	1
A0A0A8E1Q2	ATP synthase subunit beta OS=Xanthomonas sacchari OX=56458 GN=atpD PE=3 SV=1	2.35042735	1	1	10000000	0.093	1
Q8HCR5	ATP synthase protein MI25 OS=Oryza sativa subsp. japonica OX=39947 GN=orf25 PE=3 SV=2	5.583756345	1	1		0.194	1
A0A0E0FYA1	Aspartate kinase OS=Oryza nivara OX=4536 PE=4 SV=1	1.724137931	1	1	6500000	0.062	1
A0A7I0LNJ3	Aspartate carbamoyltransferase OS=Bacillus sp. SLBN-174 OX=2768463 GN=pyrB PE=3 SV=1	3.25732899	1	1		0.136	1
A0A542P235	Aspartate carbamoyltransferase OS=Arthrobacter sp. SLBN-53 OX=2768412 GN=pyrB PE=3 SV=1	3.144654088	1	1	270000000	0.145	1
A0A542JAG2	Arginine--tRNA ligase OS=Acidovorax sp. SLBN-42 OX=2768435 GN=argS PE=3 SV=1	3.590664273	1	1	41000000	0.072	1
A0A0E0GNB1	Aminotran_1_2 domain-containing protein OS=Oryza nivara OX=4536 PE=4 SV=1	2.53411306	1	1	2900000	0.089	1
A0A0E0GED2	Aminotran_1_2 domain-containing protein OS=Oryza nivara OX=4536 PE=3 SV=1	2.586206897	1	1	4700000	0.083	1
A0A5C8LRB6	Amidohydrolase family protein OS=Rheinheimera tangshanensis OX=400153 GN=FU839_12685 PE=4 SV=1	3.879310345	1	1	3200000	0.055	1
A0A542KTK7	Alpha-L-fucosidase OS=Streptomyces sp. SLBN-31 OX=2768444 GN=FBY22_3049 PE=4 SV=1	0.87804878	1	1		0.048	1
B9FVG3	Aldo_ket_red domain-containing protein OS=Oryza sativa subsp. japonica OX=39947 GN=OsJ_23062 PE=4 SV=1	4.166666667	1	1		0.11	1
A0A0E0HWE6	Aldo_ket_red domain-containing protein OS=Oryza nivara OX=4536 PE=4 SV=1	2.925531915	1	1	11000000	0.116	1
B9FHE3	AIG1-type G domain-containing protein OS=Oryza sativa subsp. japonica OX=39947 GN=OsJ_17149 PE=3 SV=1	1.055011304	1	1	6800000	0.036	1
Q7X723	Adenine phosphoribosyltransferase OS=Oryza sativa subsp. japonica OX=39947 GN=OSJNBa0014K14.11 PE=3 SV=3	2.380952381	1	1	2200000	0.116	1
Q0DLA3	Actin-depolymerizing factor 7 OS=Oryza sativa subsp. japonica OX=39947 GN=ADF7 PE=3 SV=2	8.633093525	1	1	650000	0	1
B8B9G9	Acid phosphatase OS=Oryza sativa subsp. indica OX=39946 GN=OsI_30242 PE=3 SV=1	3.728070175	1	1	890000	0.093	1
A0A0E0HLK3	AB hydrolase-1 domain-containing protein OS=Oryza nivara OX=4536 PE=3 SV=1	3.063063063	1	1	5400000	0.068	1
A0A0P0XPX5	9 kDa polypeptide OS=Oryza sativa subsp. japonica OX=39947 GN=Os09g0485201 PE=3 SV=1	11.11111111	1	1	9000000	0.334	1
A0A0E0IYY2	6-phosphogluconate dehydrogenase, decarboxylating OS=Oryza nivara OX=4536 PE=3 SV=1	1.968503937	1	1	3100000	0.086	1
B9FU88	6-hydroxymethyl-7,8-dihydropterin pyrophosphokinase OS=Oryza sativa subsp. japonica OX=39947 GN=OsJ_25143 PE=3 SV=1	2.367688022	1	1	8600000	0.049	1
A0A0K0LKH4	50S ribosomal protein L33, chloroplastic OS=Oryza sativa OX=4530 GN=rpl33 PE=3 SV=1	15.15151515	1	1	5500000	0.778	1
A0A0N9E0Z0	50S ribosomal protein L22, chloroplastic OS=Oryza sativa tropical japonica subgroup OX=1736656 GN=rpl22 PE=3 SV=1	8.724832215	1	1	11000000	0.334	1
P0DKK9	40S ribosomal protein S10-2 OS=Oryza sativa subsp. japonica OX=39947 GN=RPS10-2 PE=2 SV=1	10.38251366	1	1	700000	0.212	1
Q7Y096	3-isopropylmalate dehydrogenase OS=Oryza sativa subsp. japonica OX=39947 GN=LOC_Os03g45320 PE=3 SV=1	2.941176471	1	1	2900000	0.105	1
A6N079	30S ribosomal protein S8, chloroplastic OS=Oryza sativa subsp. indica OX=39946 GN=OsI_07208 PE=2 SV=1	10.76923077	1	1	7700000	0.233	1
A0A1W5HXK7	30S ribosomal protein S18, chloroplastic OS=Oryza grandiglumis OX=29690 GN=rps18 PE=3 SV=1	4.90797546	1	1	2300000	0.259	1
A0A0E0I3N5	(S)-2-hydroxy-acid oxidase OS=Oryza nivara OX=4536 PE=3 SV=1	3.916449086	1	1	4900000	0.075	1
