# Supplementary figures and images for: Glutathione S-transferase activity facilitates rice tolerance to the barnyard grass root exudate DIMBOA
Source: BMC Plant Biol. 2024 Feb 17;24:117. doi: 10.1186/s12870-024-04802-5 (PMC10874003; doi:10.1186/s12870-024-04802-5)

PI312777

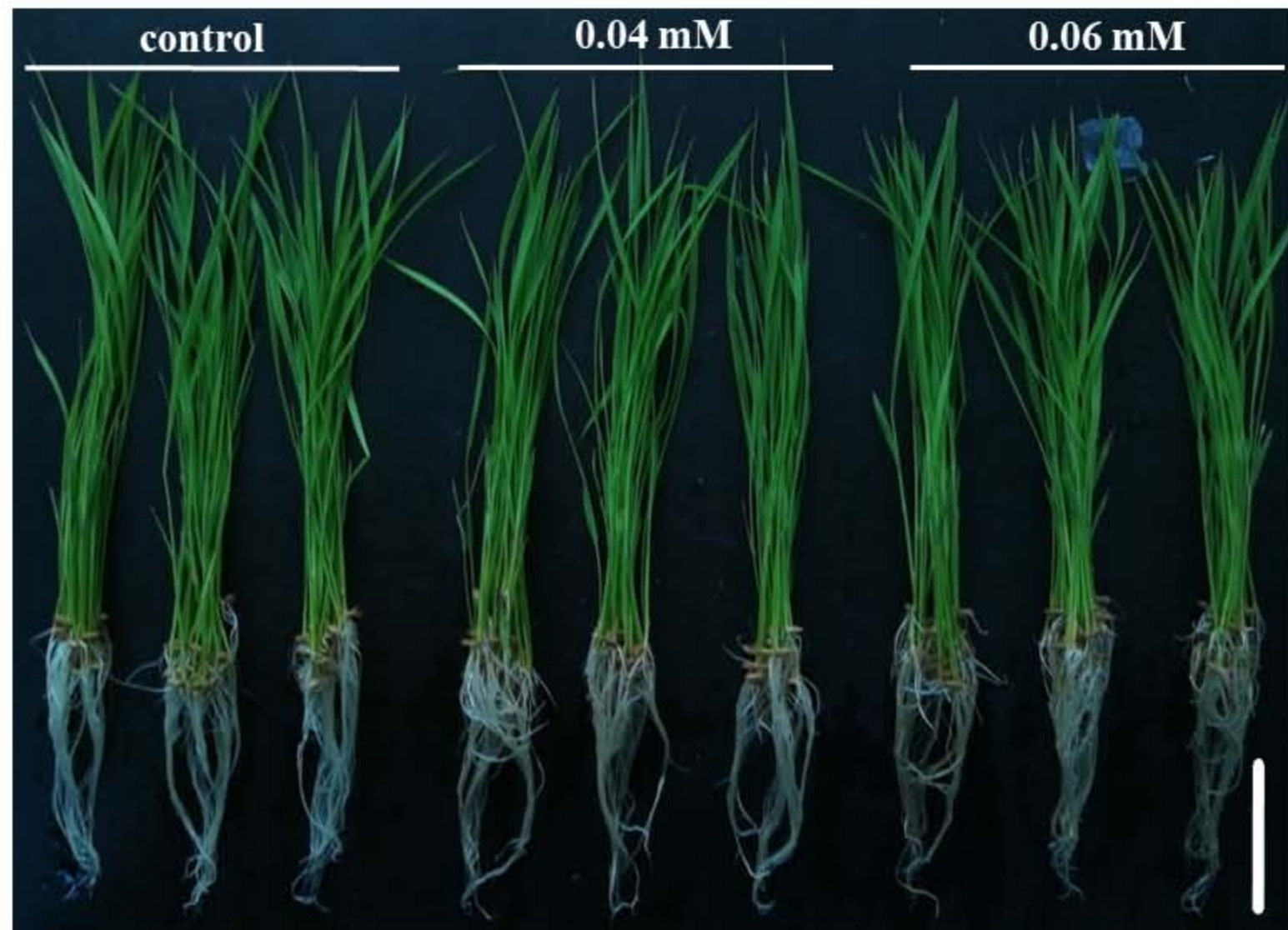

Lemont

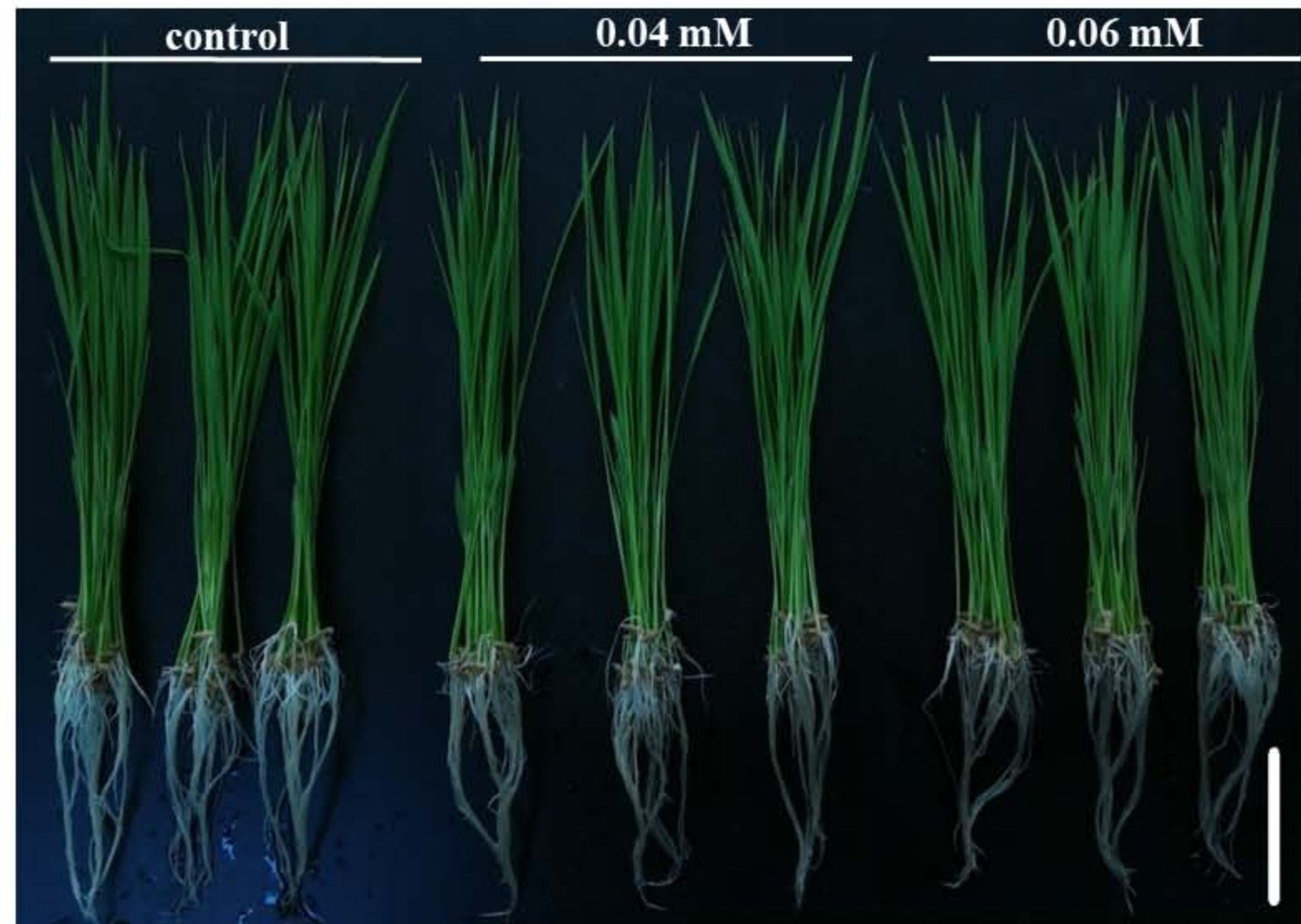

Supplement: Supplementary file 4 — Supplementary Material 4: Fig. S4. PCR amplification of the DNA fragment of GST-eYFP fused gene from Os09g0367700-OX and Os01g0949800-OX line for positive transgenic lines identification [file 12870_2024_4802_MOESM4_ESM.pdf]

PI312777

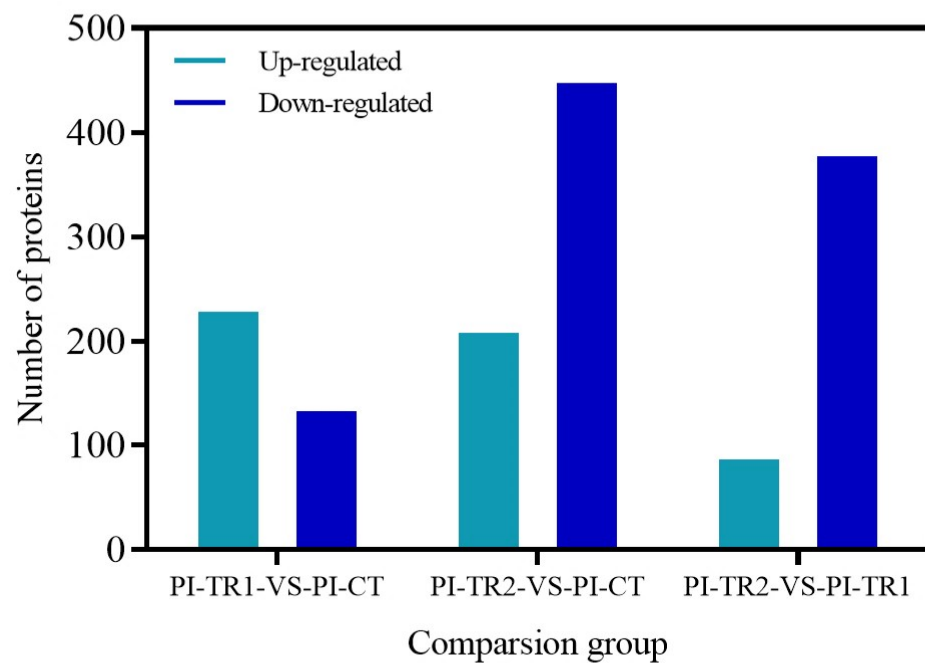

Lemont

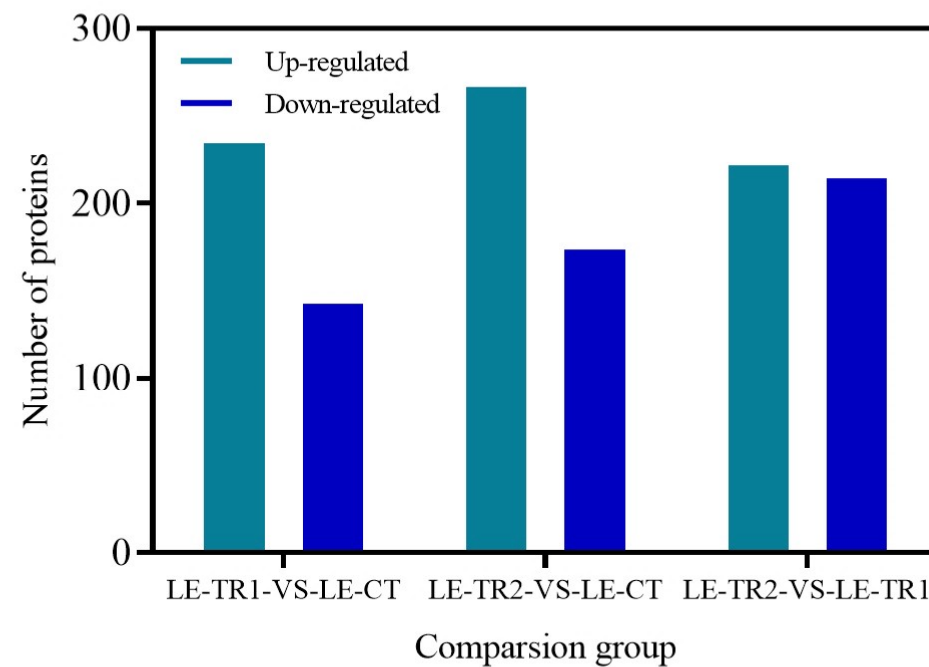

Supplement: Supplementary file 5 — Supplementary Material 5: Fig. S5. KEGG enrichment of the proteins interacting with Os09g0367700 and Os01g0949800 [file 12870_2024_4802_MOESM5_ESM.pdf]

# PI-TR1 vs. PI-CT

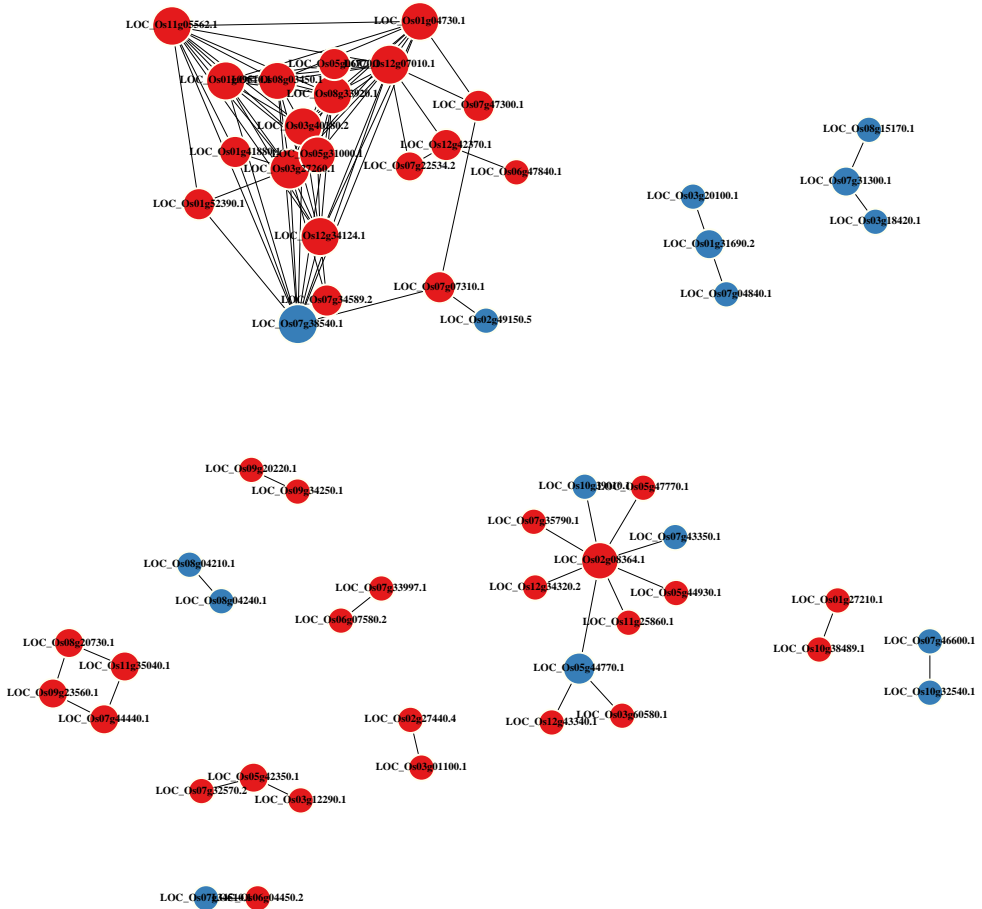

## PI-TR2 vs. PI-CT

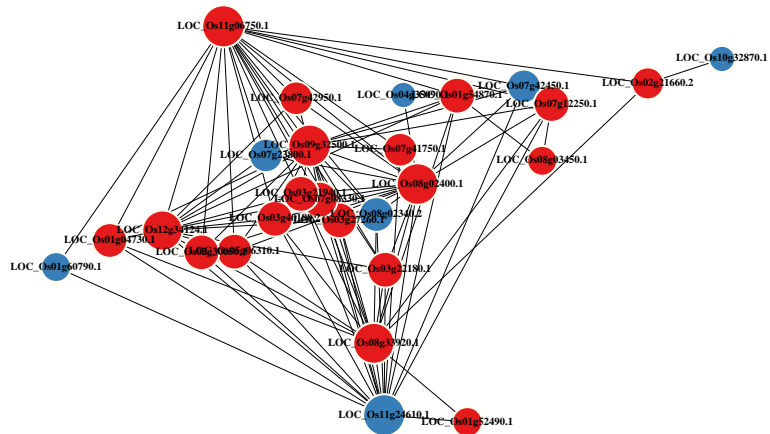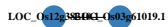

PI-TR3 vs. PI-CT

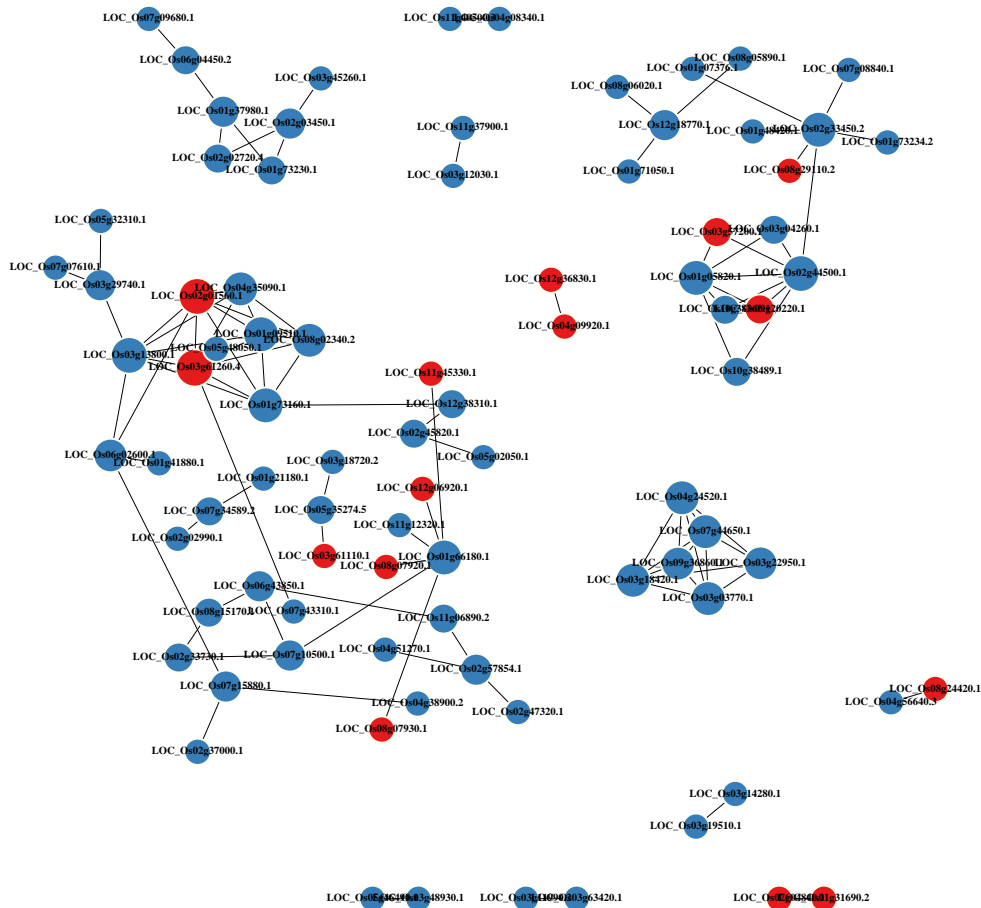

## Le-TR1 vs Le-CT

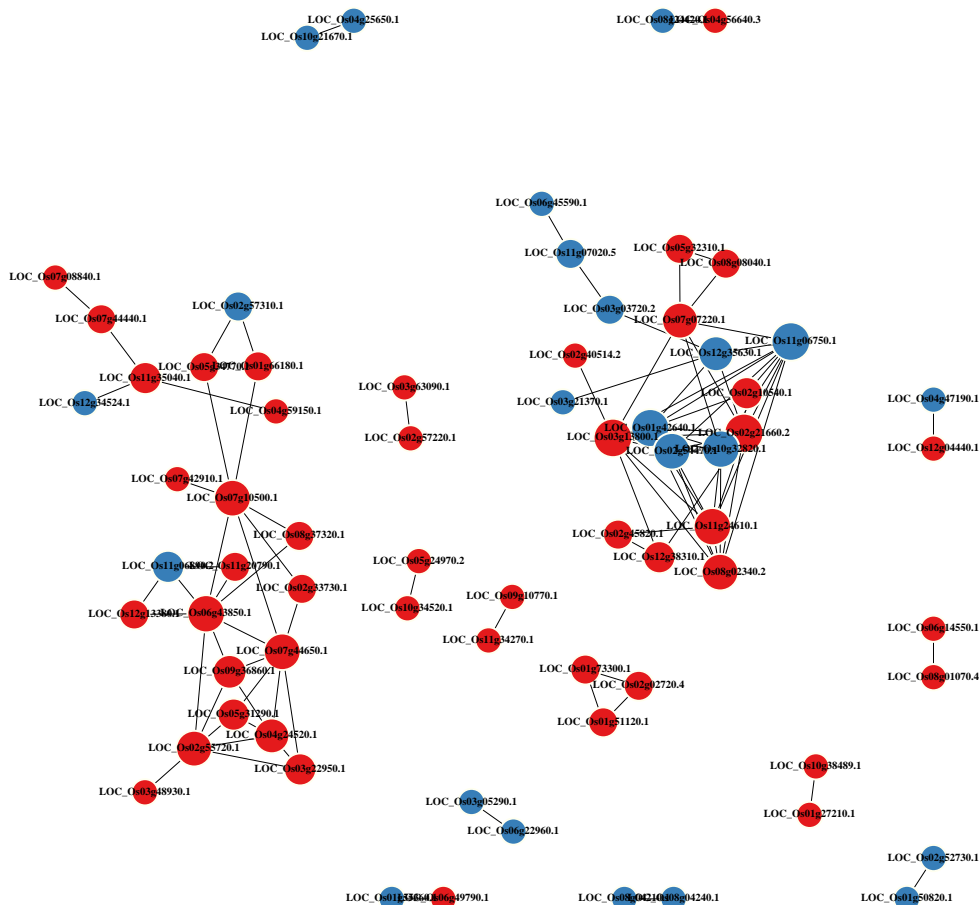

## Le-TR2 vs Le-CT

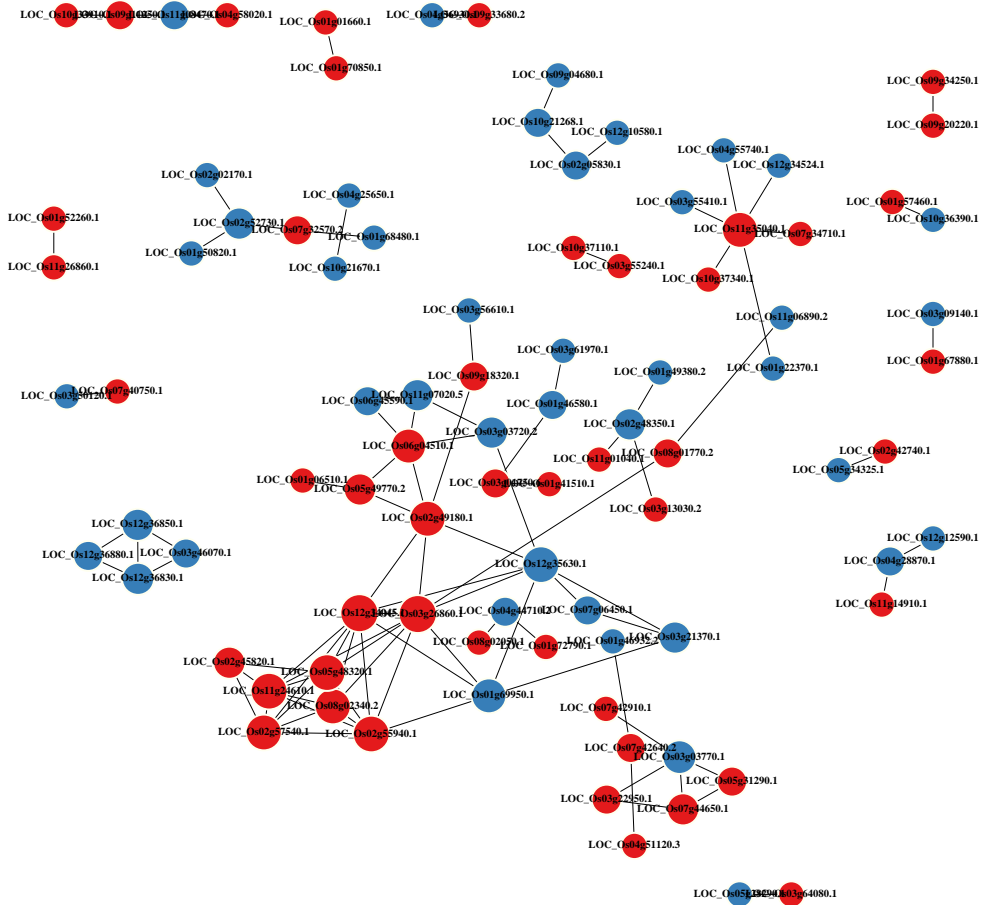

## Le-TR2 vs Le-TR1

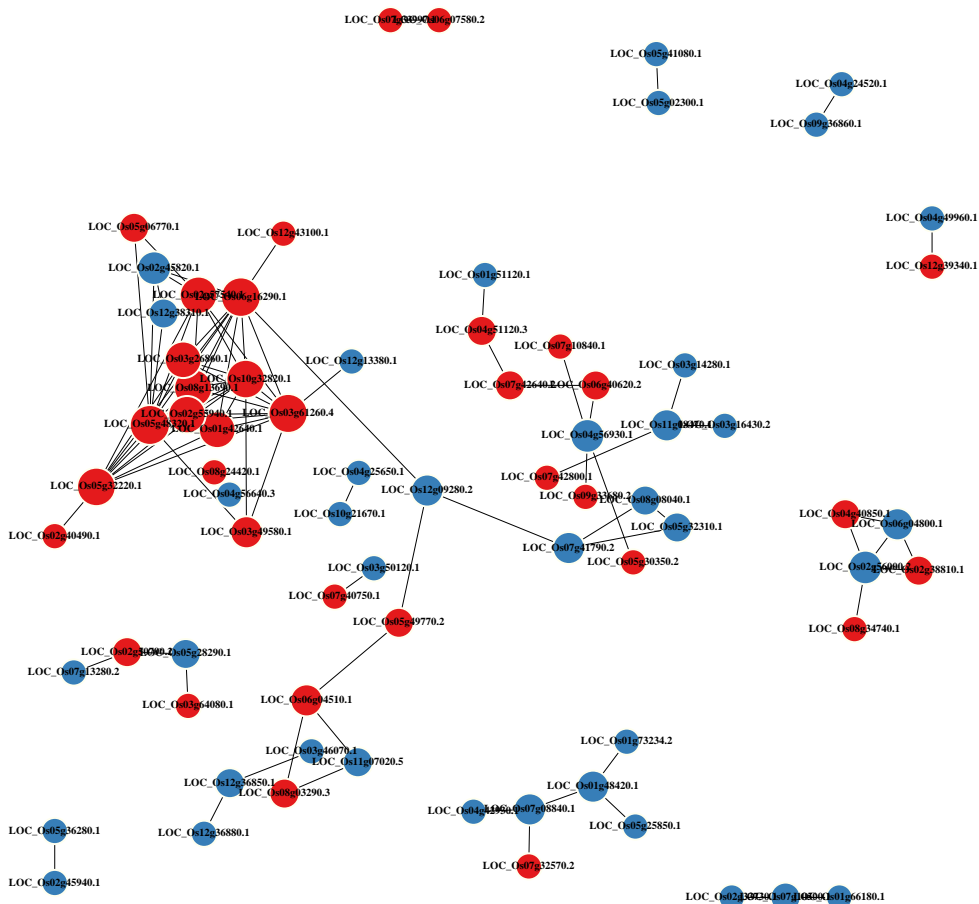

Supplement: Supplementary file 6 — Supplementary Material 6: Dataset S1. Protocol details of iTRAQ proteomics and Co-IP [file 12870_2024_4802_MOESM6_ESM.pdf]

PI312777

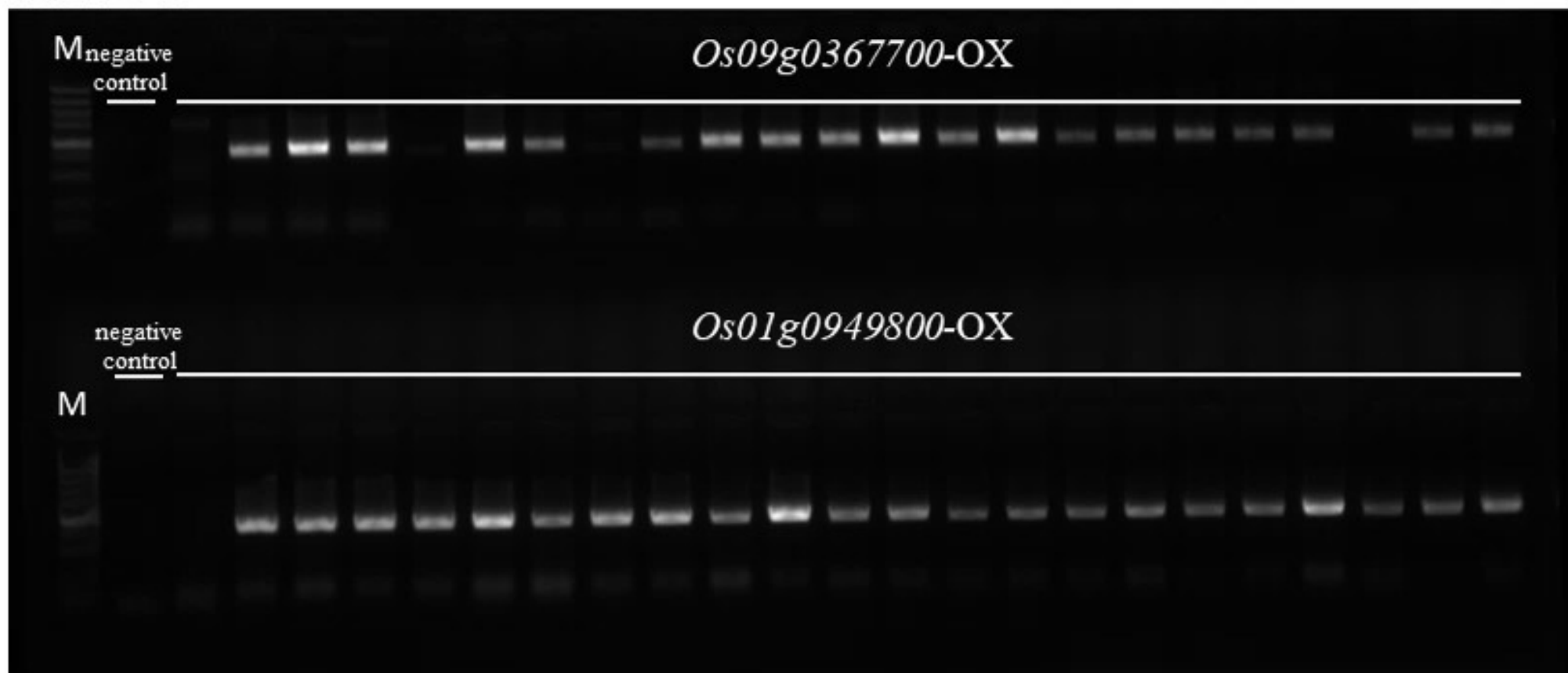

Lemont

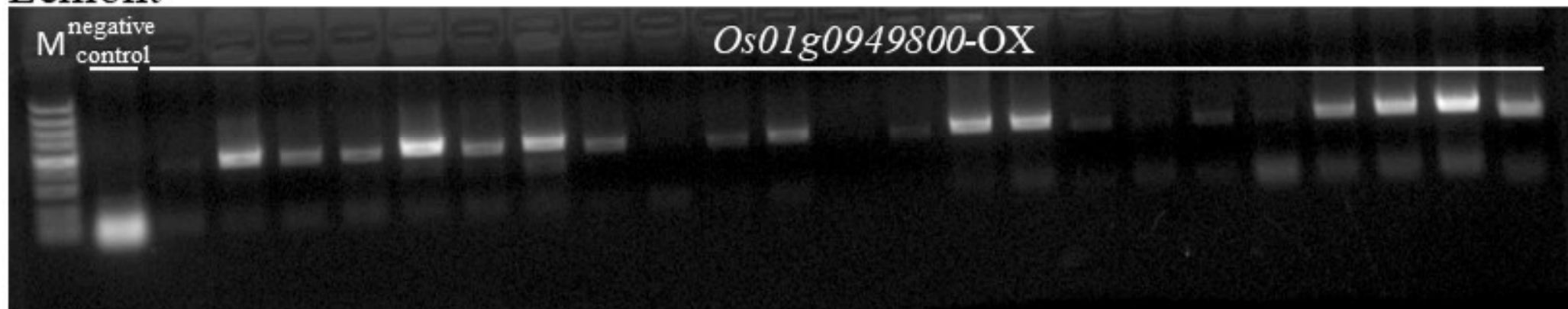

Supplement: Supplementary file 7 — Supplementary Material 7: Dataset S2. Mass spectrum identification of proteins interacting with Os09g0367700 in PI312777 [file 12870_2024_4802_MOESM7_ESM.pdf]

**(A)**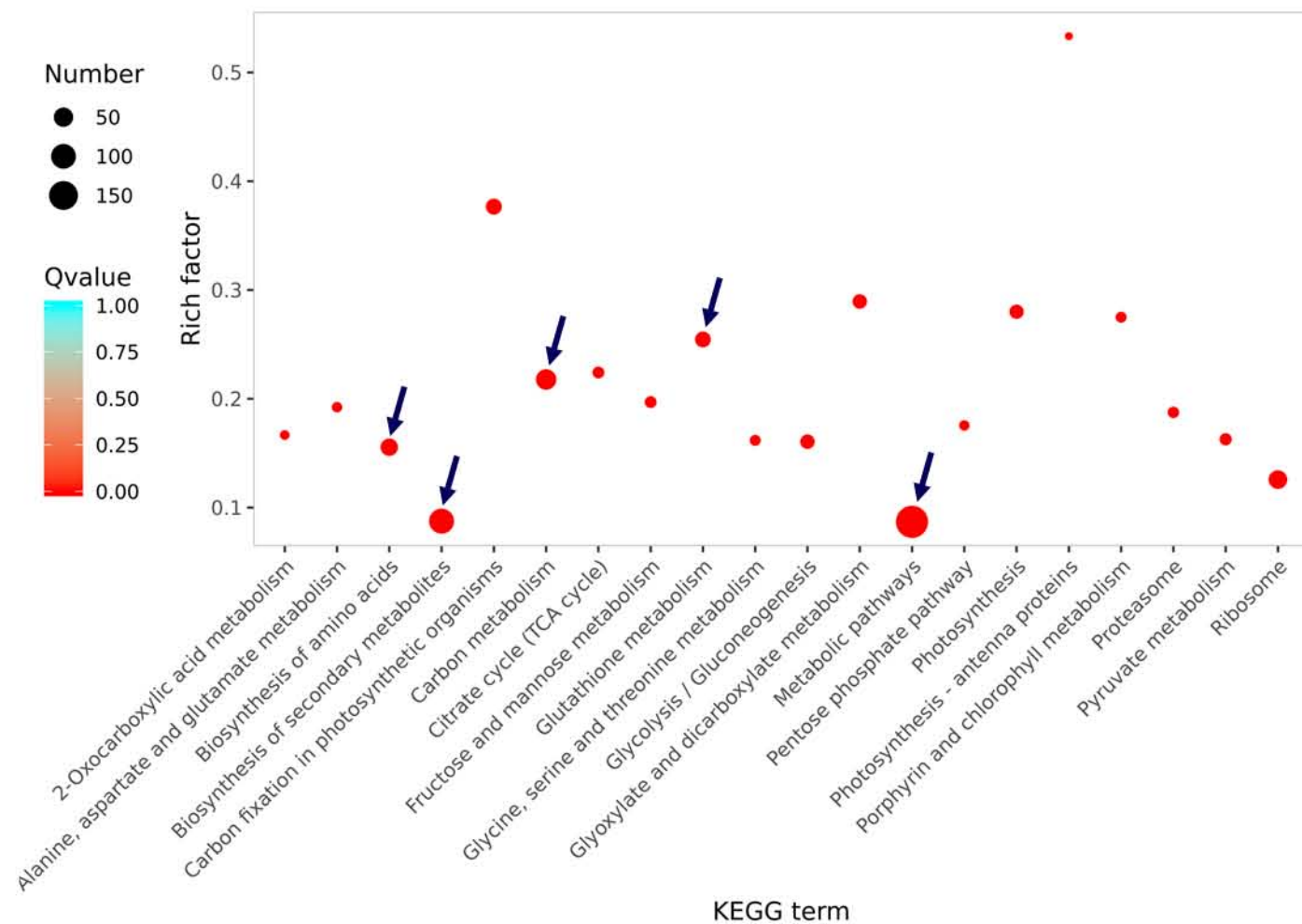**(B)**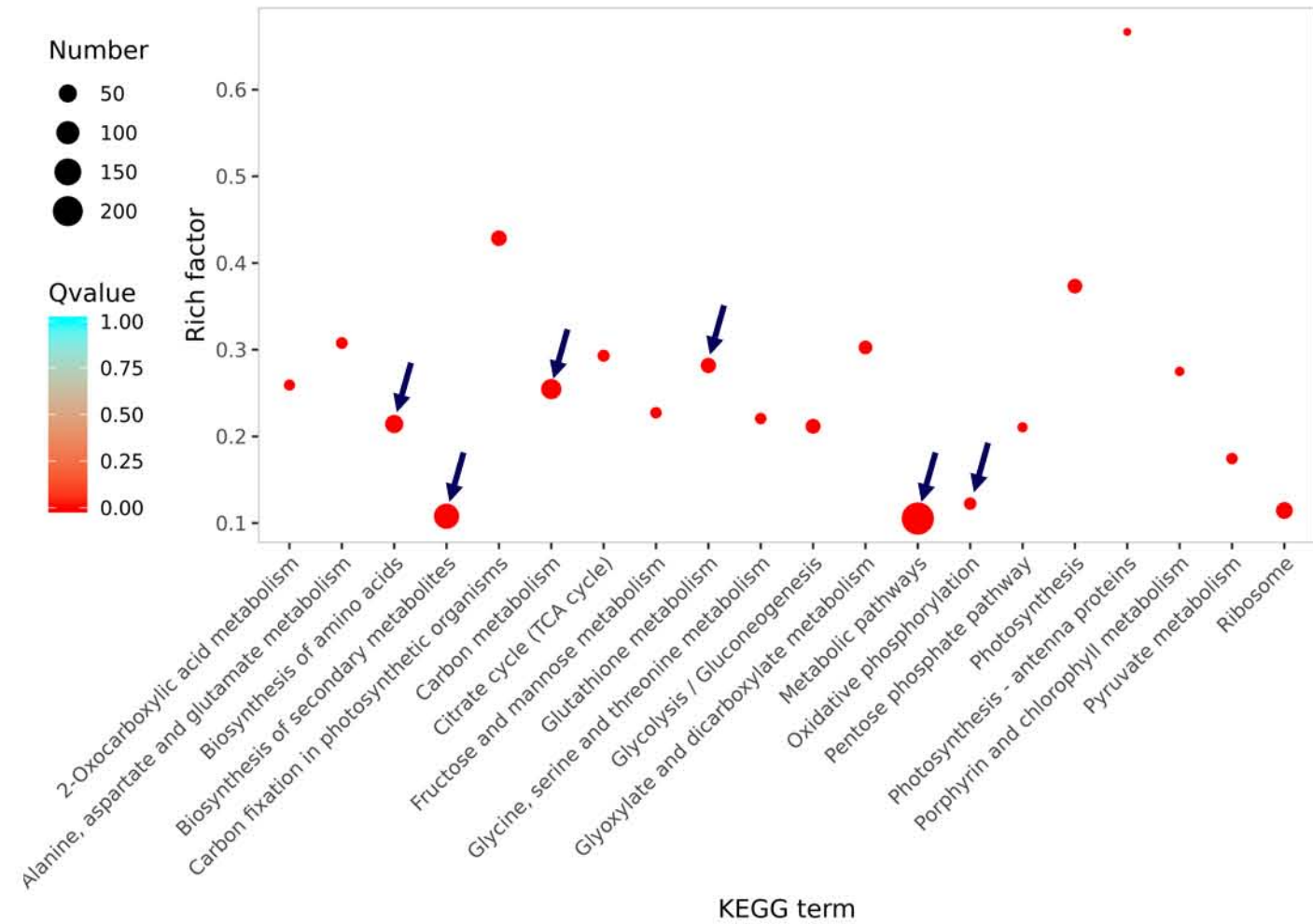

Supplement: Supplementary file 8 — Supplementary Material 8: Dataset S3. Mass spectrum identification of proteins interacting with Os01g0949800 in Lemont [file 12870_2024_4802_MOESM8_ESM.pdf]

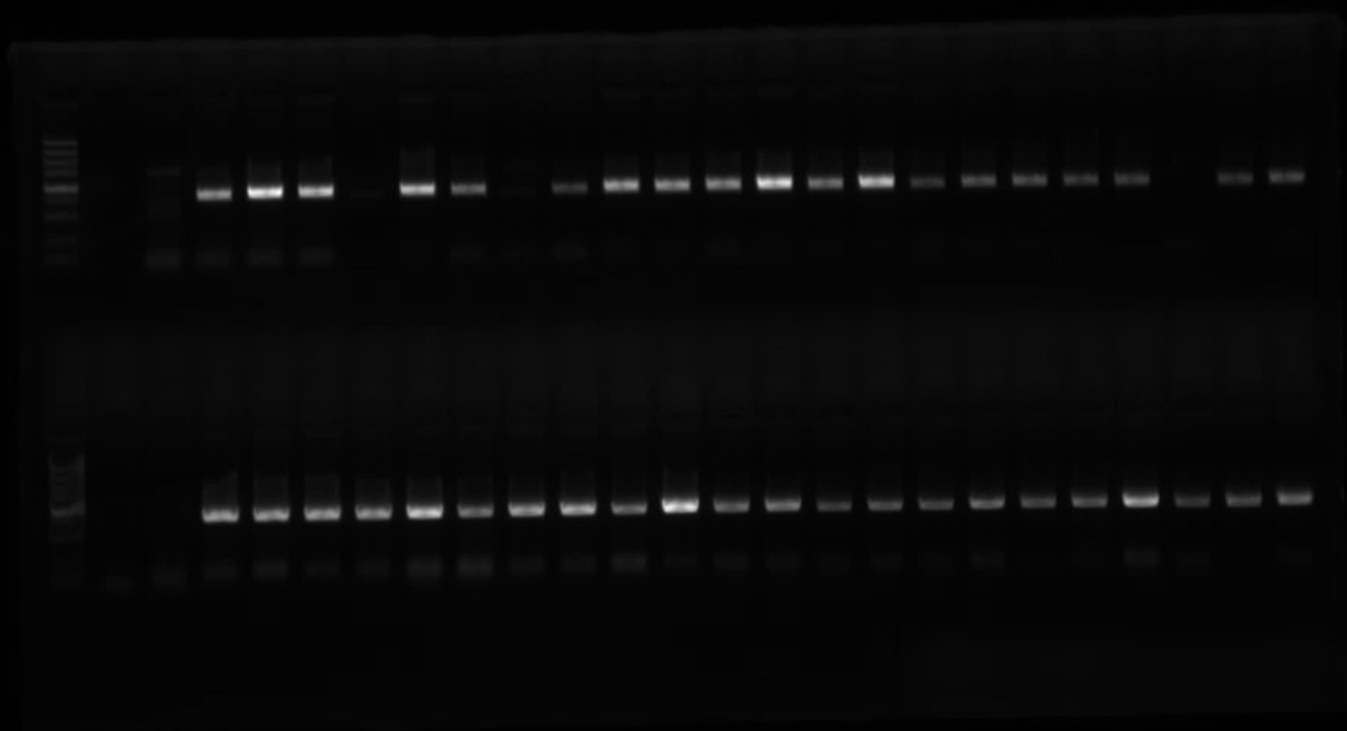

Supplement: Supplementary file 9 — Supplementary Material 9: Electronic Supplementary Material 1. Full length gel presents PCR amplification of the DNA fragment of GST-eYFP fused gene from Os09g0367700-OX and Os01g0949800-OX transgenic PI312777 lines [file 12870_2024_4802_MOESM9_ESM.pdf]

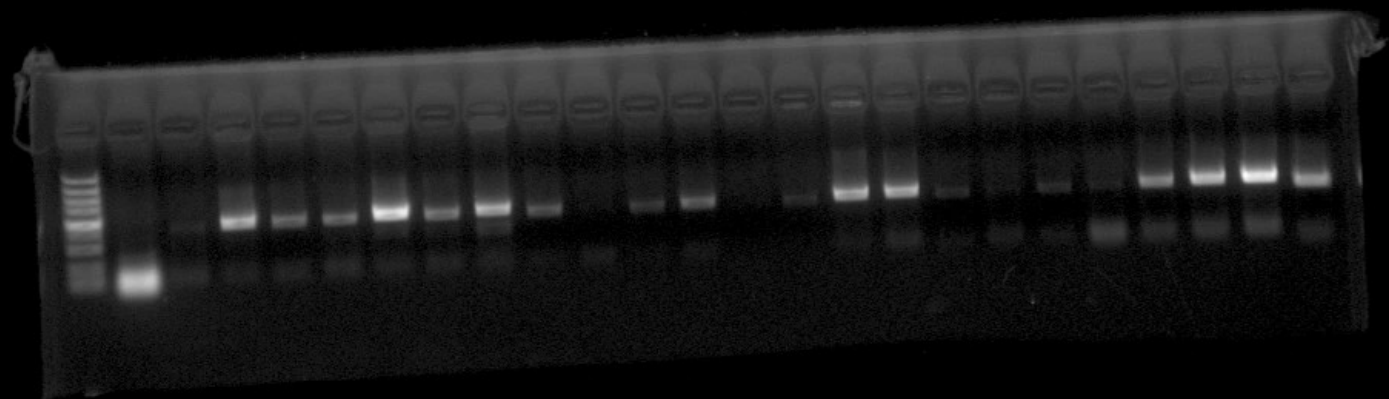

Supplement: Supplementary file 10 — Supplementary Material 10: Electronic Supplementary Material 2. Full length gel presents PCR amplification of the DNA fragment of GST-eYFP fused gene from Os01g0949800-OX transgenic Lemont line [file 12870_2024_4802_MOESM10_ESM.pdf]

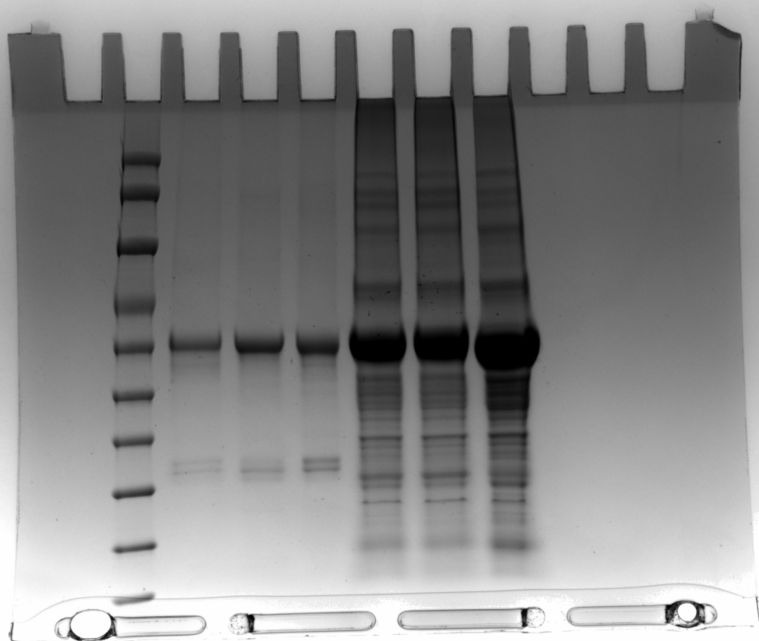

Supplement: Supplementary file 11 — Supplementary Material 11: Electronic Supplementary Material 3. Full length gel presents GST-interacting proteins from GST-OX transgenic rice without DIMBOA treatment [file 12870_2024_4802_MOESM11_ESM.pdf]

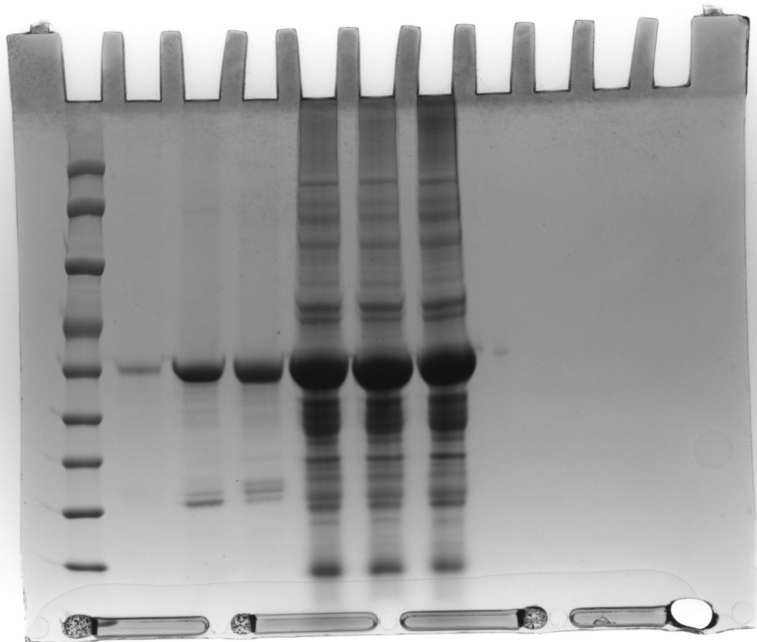

Supplement: Supplementary file 12 — Supplementary Material 12: Electronic Supplementary Material 4. Full length gel presents GST-interacting proteins from GST-OX transgenic rice with DIMBOA treatment [file 12870_2024_4802_MOESM12_ESM.pdf]
