# Supplementary material for: Glutathione S-transferase activity facilitates rice tolerance to the barnyard grass root exudate DIMBOA
Source: BMC Plant Biol. 2024 Feb 17;24:117. doi: 10.1186/s12870-024-04802-5 (PMC10874003; doi:10.1186/s12870-024-04802-5)
Supplement: Supplementary file 13 — Supplementary Material 13 [file 12870_2024_4802_MOESM13_ESM.docx]

**Supplementary Information**

**Fig. S1.** Phenotype of PI312777 and Lemont seedlings after treatment with DIMBOA. Bar, 5cm

**Fig. S2.** Amounts of differentially expressed proteins from the roots of PI312777 and Lemont after treatment with DIMBOA

**Fig. S3.** Predicted protein‒protein interactions among differentially expressed proteins from DIMBOA-treated PI312777 and the control group. Red nodes represent upregulated proteins; blue nodes represent downregulated proteins.

**Fig. S4. PCR amplification of the DNA fragment of GST-eYFP fused gene from** *Os09g0367700*-OX and *Os01g0949800*-OX line for positive transgenic lines identification

**Fig. S5.** KEGG enrichment of the proteins interacting with Os09g0367700 and Os01g0949800

**Dataset S1.** Protocol details of iTRAQ proteomics and Co-IP

**Dataset S2.** Mass spectrum identification of proteins interacting with Os09g0367700 in PI312777

**Dataset S3.** Mass spectrum identification of proteins interacting with Os01g0949800 in Lemont

**Electronic Supplementary Material** **1**

Full length gel presents PCR amplification of the DNA fragment of GST-eYFP fused gene from *Os09g0367700*-OX and *Os01g0949800*-OX transgenic PI312777 lines

**Electronic Supplementary Material** **2**

Full length gel presents PCR amplification of the DNA fragment of GST-eYFP fused gene from *Os01g0949800*-OX transgenic Lemont line

**Electronic Supplementary Material** **3**

Full length gel presents GST-interacting proteins from GST-OX transgenic rice without DIMBOA treatment

**Electronic Supplementary Material** **4**

Full length gel presents GST-interacting proteins from GST-OX transgenic rice with DIMBOA treatment
